# Cellular metabolism changes in atherosclerosis and the impact of comorbidities

**DOI:** 10.3389/fcell.2024.1446964

**Published:** 2024-08-12

**Authors:** Yusang Dai, Carolina Victoria Cruz Junho, Luisa Schieren, Julia Wollenhaupt, Judith C. Sluimer, Emiel P. C. van der Vorst, Heidi Noels

**Affiliations:** ^1^ Institute for Molecular Cardiovascular Research (IMCAR), University Hospital, RWTH Aachen University, Aachen, Germany; ^2^ Physical Examination Center, The Affiliated Hospital of Guizhou Medical University, Guiyang, China; ^3^ Department of Nephrology and Clinical Immunology, University Hospital RWTH Aachen, Aachen, Germany; ^4^ Department of Pathology, Cardiovascular Research Institute Maastricht (CARIM), Maastricht University, Maastricht, Netherlands; ^5^ Aachen-Maastricht Institute for Cardiorenal Disease (AMICARE), RWTH Aachen Campus, Aachen, Germany; ^6^ Interdisciplinary Centre for Clinical Research (IZKF), RWTH Aachen University, Aachen, Germany; ^7^ Institute for Cardiovascular Prevention (IPEK), Ludwig-Maximilians-University Munich, Munich, Germany; ^8^ Department of Biochemistry, Cardiovascular Research Institute Maastricht (CARIM), Maastricht University, Maastricht, Netherlands

**Keywords:** atherosclerosis, cell metabolism, glycolysis, fatty acids, diabetes, comorbidity

## Abstract

Cell activation and nutrient dysregulation are common consequences of atherosclerosis and its preceding risk factors, such as hypertension, dyslipidemia, and diabetes. These diseases may also impact cellular metabolism and consequently cell function, and the other way around, altered cellular metabolism can impact disease development and progression through altered cell function. Understanding the contribution of altered cellular metabolism to atherosclerosis and how cellular metabolism may be altered by co-morbidities and atherosclerosis risk factors could support the development of novel strategies to lower the risk of CVD. Therefore, we briefly review disease pathogenesis and the principles of cell metabolic pathways, before detailing changes in cellular metabolism in the context of atherosclerosis and comorbidities. In the hypoxic, inflammatory and hyperlipidemic milieu of the atherosclerotic plaque riddled with oxidative stress, metabolism shifts to increase anaerobic glycolysis, the pentose-phosphate pathway and amino acid use. We elaborate on metabolic changes for macrophages, neutrophils, vascular endothelial cells, vascular smooth muscle cells and lymphocytes in the context of atherosclerosis and its co-morbidities hypertension, dyslipidemia, and diabetes. Since causal relationships of specific key genes in a metabolic pathway can be cell type-specific and comorbidity-dependent, the impact of cell-specific metabolic changes must be thoroughly explored *in vivo*, with a focus on also systemic effects. When cell-specific treatments become feasible, this information will be crucial for determining the best metabolic intervention to improve atherosclerosis and its interplay with co-morbidities.

## 1 Introduction

Atherosclerosis describes the accumulation of immune cell-rich, lipid-rich plaques known as atheroma in large and medium-sized arteries (Roy et al., 2022). Around the world, atherosclerosis is a major contributor to cardiovascular diseases (CVDs), which include myocardial infarction and stroke, amongst others (Libby, 2021). CVDs are extremely common and have been steadily rising for decades to become the world’s top cause of death (Roth et al., 2020). Currently, CVD accounts for almost four million deaths in Europe annually, or almost 44% of all deaths, with ischemic heart disease accounting for 44% of these CVD deaths and stroke accounting for 25% (Townsend et al., 2022). In 2016, China had around 2.4 million deaths from atherosclerotic CVD (ASCVD), accounting for 61% of CVD deaths and 25% of all deaths. This roughly represents a doubling of the absolute and relative number of deaths (1.4 million deaths from ASCVD, 11% of all deaths) compared to 1990 (Zhao et al., 2019).

Atherosclerosis is the narrowing of arteries caused by endothelial dysfunction, the accumulation of excess lipids in the vessel intima and the associated recruitment of inflammatory cells such as monocytes/macrophages. These undergo foam cell formation by taking up lipids and drive inflammatory processes. Atherosclerotic plaque formation occurs preferentially at artery branch points, which show a disturbed, non-laminar blood flow and are particularly vulnerable to inflammation and oxidative stress caused by hyperlipidemia. During all stages of atherogenesis, endothelial dysfunction, oxidative stress and inflammation contribute to disease development and progression (Hurtubise et al., 2016; Baaten et al., 2023). Also, neutrophils, B- and T-lymphocytes contribute to atherosclerosis (Nus and Mallat, 2016). Furthermore, vascular smooth muscle cells (VSMCs) in the tunica media can migrate into the intima, where they produce extracellular matrix molecules, forming a protective fibrous cap over the developing lesion (Sano et al., 2001; Bennett et al., 2016; Basatemur et al., 2019). On the other hand, VSMCs can also support lesion progression via SMC apoptosis or through differentiation into fibrochondrocyte-like or into macrophage-like cells that contribute to foam cell formation (Bennett et al., 2016; Basatemur et al., 2019). Plaque rupture is a main process leading to thrombus formation. Furthermore, superficial erosion is a rising contributor to the formation of thrombi in the coronary arteries and involves a shedding of the endothelial cell (EC) layer, mostly at plaques that show less lipid and macrophage accumulation, but a higher extracellular matrix content compared to plaques prone to rupture (Franck et al., 2017; Libby et al., 2019).

Patients with hypertension, dyslipidemia, type 2 diabetes mellitus (T2DM) or chronic kidney disease (CKD) have an increased risk of atherosclerosis (Selvin et al., 2005; Speer et al., 2022; Noels and Jankowski, 2023). Increased low-density lipoprotein-cholesterol (LDL-C), non-high-density lipoprotein cholesterol (non-HDL-C) and triglycerides, but a decline in HDL-C all independently increase the risk of ASCVD (Kopin and Lowenstein, 2017; Soppert et al., 2020). LDL and its oxidized form oxLDL play a key role in endothelial damage, macrophage foam cell formation and necrosis, as well as inflammatory signaling, whereas HDL plays an atheroprotective role by driving reverse cholesterol transport and by its anti-inflammatory properties (Soppert et al., 2020). T2DM is characterized by chronic hyperglycemia. Furthermore, diabetes can lead to hyperlipidemia and atherosclerosis due to an increased hepatic synthesis of triglyceride-rich very low-density lipoprotein (VLDL) (Poznyak et al., 2020). Hyperinsulinemia, insulin resistance, hyperglycemia and lipotoxicity all contribute to the creation of advanced glycation end products (AGEs), elevated free fatty acids and oxLDL, and can contribute to endothelial dysfunction, macrophage foam cell formation, inflammation and phenotypic switching of VSMCs, thereby supporting atherosclerosis (Zhao et al., 2024). Hypertension contributes to atherosclerosis through endothelial dysfunction as well as a hyperactivation of the renin-angiotensin-aldosterone system (RAAS) and the sympathetic nervous system (SNS) (Zaheer et al., 2016). CKD - defined as kidney damage or a glomerular filtration rate below 60 mL/min/1.73 m^2^ for three or more months - is an independent risk factor for the development of CVD, including atherosclerosis, with CKD patients displaying a higher prevalence and progression of atherosclerotic lesions (Valdivielso et al., 2019; Noels and Jankowski, 2023). CKD contributes to atherosclerosis not only by increasing traditional risk factors like hypertension (Valdivielso et al., 2019), dyslipidemia and inflammation, but also through CKD-specific alterations like albuminuria and the accumulation of uremic retention solutes (Harlacher et al., 2022; Baaten et al., 2023; Vondenhoff et al., 2024).

In the past decade, a dysregulation of cellular metabolism has come into focus, leading to the identification of cell metabolism alterations in atherosclerotic lesions (Tomas et al., 2018) as well as in different cell types involved in atherogenesis in hyperlipidemic or/and pro-inflammatory context (Tomas et al., 2018). Understanding atherosclerosis and the contribution of altered cellular metabolism, as well as the impact of comorbidities on this, could support the design of novel strategies to lower the risk of CVD. Consequently, in this review, we will discuss cellular metabolism changes in the context of atherosclerosis and elaborate on how comorbidities may impact on atherosclerosis through effects on cellular metabolism.

## 2 Metabolic changes in atherosclerotic plaques

In the last decade, many efforts have been invested in exploring cell metabolism changes in relation to disease, especially in relation to immune cells and inflammatory diseases. Immune cells can make use of different substrates and energy metabolic pathways to produce energy in the form of ATP through processes of glycolysis, fatty acid oxidation (FAO) and amino acid metabolism, coupled to the tricarboxylic acid (TCA) cycle and oxidative phosphorylation (OXPHOS) cycle. Such breakdown of substrates to produce energy is referred to as “cellular respiration.” Furthermore, metabolites generated during these processes are used to support cellular functions and survival and can modulate cell signaling pathways to influence both pro-inflammatory and anti-inflammatory outcomes (O’Neill et al., 2016; Voss et al., 2021). Depending on the context, alterations in cell metabolism can be observed. For example, upon infection or inflammation, macrophages from the innate immune system, as well as B-cells as cells from the adaptive immune system increase glycolysis and downregulate FAO, which has been linked with cellular activation profiles required to fight inflammation (Rhoads et al., 2017). However, also in context of diseases, cell metabolism alterations have been observed and over the past years, multiple studies focused on understanding cell metabolism changes in various pathophysiological conditions, including inflammation and atherosclerosis - with the aim to develop new therapeutic strategies.

In 2002, it was shown that symptomatic, unstable plaques demonstrated increased uptake of the glucose analogue [18F]-fluorodeoxyglucose compared to asymptomatic plaques, whereas healthy carotid arteries did not show glucose uptake (Rudd et al., 2002). Furthermore, in an elegant study addressing the relation between the metabolic profile and vulnerability of human atherosclerotic lesions, Tomas et al. described in 2018 two distinct clusters of carotid artery plaques based on metabolite profiling and revealed that the metabolic profile was indicative for both the vulnerability of the lesions as well as future cardiovascular risk (Tomas et al., 2018). Plaques from symptomatic patients and with high vulnerability – characterized by a high lipid and macrophage content, low SMC and collagen content and signs of hemorrhages – showed metabolic alterations in terms of metabolite quantities and transcription levels of metabolic enzymes compared to plaques from asymptomatic patients and with low vulnerability (Tomas et al., 2018). More specifically, the high-risk plaques showed signs of reduced FAO (with a decrease in short-chain acylcarnitines) but increased glycolysis (with reduced levels of glucose but increased lactate) and amino acid use (with reduced substrates of glutamine and serine) (Tomas et al., 2018). In parallel, high-risk plaques showed increased mRNA levels of genes involved in glycolysis and the pentose-phosphate pathway as well as a stronger pro-inflammatory profile and were also associated with a higher risk of cardiovascular events over a seven-year follow-up (Tomas et al., 2018).

Recently, this was confirmed by Seeley et al., which in addition showed the spatial location of metabolites revealing the differential presence of lactic acid in the necrotic core of stable plaques, while pyruvic acid was more prevalent in the fibrous cap. In the fibrous cap of unstable plaques, 5-hydroxyindoleacetic acid was more prevalent (Seeley et al., 2023). Combined, this supported the notion that cellular metabolism may play a crucial role in supporting inflammation and the high-risk phenotype of atherosclerotic plaques (Tomas et al., 2018). Nevertheless, how metabolism of individual cell types changes, remains to be discovered.

Before elaborating on metabolic alterations in different cell types involved in atherosclerosis, important metabolic pathways are first introduced in the next section.

## 3 Brief description of important metabolic pathways

### 3.1 The tricarboxylic acid (TCA) cycle (also called Krebs cycle or citric acid cycle)

The TCA cycle takes place within the mitochondrial matrix and is a central regulator of energy production by oxidation of acetyl-coenzyme A (acetyl-CoA) derived from the metabolism of carbohydrates (and more specifically from glucose-derived pyruvate), fatty acids as well as proteins (Alabduladhem and Bordoni, 2024). Acetyl-CoA is used to produce citrate from oxaloacetate in the first step of the TCA cycle. Then, citrate is subsequently converted to other metabolites in eight successive steps, ultimately resulting in the regeneration of citrate. Each complete turn of the TCA cycle generates one GTP molecule as well as three molecules of NADH - the reduced form of nicotinamide adenine diphosphate (NAD)- and one molecule of FADH_2_ – the reduced form of flavin adenine dinucleotide (FAD) -, which will enter the oxidative phosphorylation pathway to further generate energy in the form of ATP (Arnold and Finley, 2023). Metabolites generated during the TCA cycle - such as acetyl-CoA, citrate, aconitate, succinate and fumarate - can alter the response of both the innate and adaptive immune systems (Martínez-Reyes and Chandel, 2020).

### 3.2 “Oxidative phosphorylation” (OXPHOS) or “electron transport chain-linked phosphorylation”

OXPHOS follows the TCA cycle and is the final step of cellular respiration. It takes place at the inner mitochondrial membrane. In this process, the molecules NADH and FADH_2_ - derived from the TCA cycle or from the OXPHOS cycle of fatty acid metabolism - are oxidized and thereby function as electron donors in a series of redox reactions (also referred to as the electron transport chain) with oxygen as ultimate electron acceptor (Mehta et al., 2017; Judge and Dodd, 2020). During this process, electrons are pumped over the inner mitochondrial membrane, generating a proton gradient that in a subsequent step can trigger energy release to power the enzyme ATPase for the production of ATP from ADP.

### 3.3 The glycolytic pathway or glycolysis

Glucose is an important substrate for cellular energy production and is metabolized by a process called glycolysis, which occurs in the cytoplasm. With the help of the enzymes hexokinase, phosphofructokinase, 6-phosphofructo-2-kinase/fructose-2,6-bisphosphatase-3 (PFKFB3) and pyruvate kinase, glycolysis rapidly converts a single molecule of glucose into two molecules of pyruvate with a net gain of two ATP molecules and two molecules of NADH. Pyruvate can subsequently be transformed to lactate with the help of the enzyme lactate dehydrogenase and is then secreted from the cell. Alternatively, in the presence of sufficient oxygen, pyruvate can be transported into the mitochondria, where it will be converted to acetyl-CoA to fuel the TCA cycle for additional ATP production in the mitochondria (Chandel, 2021a). Overall, this mitochondrial respiration via the TCA cycle and OXPHOS can generate up to 36 ATP molecules per glucose molecule, making it much more efficient in terms of energy production compared to solely glycolysis taking place in the cytoplasm. However, glycolysis also provides the cells with different substrate intermediates that are required for the synthesis of nucleotides (glucose-6-phosphate, for nucleotide production via the pentose phosphate pathway), amino acids (3-phosphoglycerate for serine biosynthesis) and fatty acids (via glyceraldehyde 3-phospate) (Chandel, 2021a). This explains why also in aerobic conditions, glycolysis is heavily used by cells with a high anabolic need (i.e., a need of building blocks for the biosynthesis of nucleotides, lipids or proteins), even despite that such “aerobic glycolysis” (i.e., the conversion of glucose into pyruvate and subsequently lactate in the cytoplasm) is much less efficient in ATP generation compared to shuttling glucose-derived pyruvate to mitochondrial respiration (i.e., into the TCA/OXPHOS cycle). For example, glycolysis has a crucial role in the metabolism of rapidly proliferating cells (O’Neill et al., 2016) and many proliferation-inducing signaling pathways - including the phosphatidylinositol 3-kinase (PI3K) and mitogen-activated protein kinase (MAPK) pathways - enhance the cellular usage of glycolytic metabolism (Papa et al., 2019). In oncology, this has been referred to as the “Warburg effect” displayed by highly proliferative cancer cells. Similarly, the inflammatory activation of macrophages has been shown to induce increased glucose uptake and a shift from OXPHOS to aerobic glycolysis with enhanced pyruvate to lactate conversion (O’Neill and Hardie, 2013), as discussed in more detail below.

### 3.4 The pentose phosphate pathway (PPP)

The pentose phosphate pathway (PPP) is another glucose-metabolizing pathway that takes place within the cytoplasm and crucially contributes to *de novo* nucleotide synthesis, amino acid production, as well as the biosynthesis of fatty acids and triacylglycerol, but not to the formation of ATP (Dionisio et al., 2023; TeSlaa et al., 2023). Two branches can be distinguished, dependent on the initial glycolytic substrate that is being used.

The non-oxidative branch of the PPP uses fructose 6-phosphate and glyceraldehyde 3-phosphate as initial substrates; it allows intermediates from the glycolytic pathway to be channeled into the production of nucleotides through the production of ribose 5-phosphate, and can thereby contribute to cell growth and proliferation (O’Neill et al., 2016). The other way around, this branch of the PPP can also convert pentoses back to intermediates of the glycolysis pathway. The oxidative branch of the PPP metabolizes glucose 6-phosphate and is required for the generation of NADPH (the reduced form of nicotinamide adenine dinucleotide phosphate). NADPH plays an important role in maintaining a favorable cellular redox environment, producing reactive oxygen species (ROS) via NADPH-dependent enzymes but also in preventing cellular oxidative stress (TeSlaa et al., 2023). Furthermore, NADPH is required for several cellular biochemical reactions such as the synthesis of fatty acids and triacylglycerol, nucleotides as well as amino acids (O’Neill et al., 2016; Dionisio et al., 2023).

### 3.5 Fatty acid metabolism/fatty acid β-oxidation (FAO)

Beyond glucose, cells can use fatty acids for energy production, which are metabolized through FAO in the mitochondria. FAO converts fatty acids into the energy-generating products acetyl-CoA, NADH and FADH_2_, which are subsequently used in the TCA cycle and electron transport chain to generate ATP (Soppert et al., 2020). To be able to enter FAO metabolism, fatty acids are first converted to fatty acid-acyl-CoA in the cytosol (O’Neill et al., 2016). Then, they are transported to the mitochondria by either passive diffusion (for short-chain fatty acids) or with the help of carnitine palmitoyl transferase I (CPT1) and CPT2 (for medium- and long-chain fatty acids) to initiate the FAO process (Mehta et al., 2017). For each step of FAO, fatty acids are gradually shortened (with two carbons per step) with a parallel production of acetyl-CoA and the electron donors NADH and FADH_2_. Fatty acid oxidation is a strong energy producer and can generate >100 ATP molecules per fatty acid molecule as palmitate (O’Neill et al., 2016).

### 3.6 Fatty acid synthesis (FAS)

Opposite from FAO, fatty acid synthesis (FAS) is an anabolic process which uses products derived from several other metabolic pathways - being glycolysis, the TCA cycle and the PPP - to synthesize fatty acids in the cytoplasm (O’Neill et al., 2016). The activity of the FAS pathway is intimately linked to mammalian target of rapamycin (mTOR) signaling, which promotes FAS by inducing sterol regulatory element binding protein (SREBP), which in turn activates fatty acid synthase (FASN) and CoA carboxylase (ACC) (Jones and Pearce, 2017). During FAS, acetyl-CoA is converted with the help of malonyl-CoA to a growing fatty acid chain, such as palmitic acid as the most abundant saturated fatty acid in the human body. Other fatty acids can be generated through processes of fatty acid elongation and desaturation. The resulting fatty acids can be esterified to glycerol-3-phosphate or cholesterol to form triglycerides or cholesterol esters, respectively (Vassiliou and Farias-Pereira, 2023). Finally, these fatty acids are packaged into VLDL to enter the bloodstream to be delivered to tissues in our body (Judge and Dodd, 2020). VLDL can be converted to the atherogenic LDL (Soppert et al., 2020).

### 3.7 Amino acid metabolism

Amino acids (AAs) are the individual monomers that make up proteins. They can be classified into essential AAs (Histidine, Isoleucine, Leucine, Lysine, Methionine, Phenylalanine, Threonine, Tryptophan, Valine), conditionally essential AAs (Arginine, Cysteine, Glutamic Acid, Tyrosine, Glycine, Ornithine, Proline, Serine) and non-essential AAs (Alanine, Asparagine, Aspartic Acid, Glutamine) (Judge and Dodd, 2020). Furthermore, AAs can play some specific roles in metabolism. For example, glutamate can function as a nitrogen donor and acceptor and thereby can facilitate nitrogen movement among amino acids (Chandel, 2021b). Higher levels of glutamate in blood are linked to elevated estimates of both total and visceral adiposity, along with dyslipidemia and insulin resistance. Additionally, glutamate plasma levels are increased with the occurrence of subclinical atherosclerosis (Lehn-Stefan et al., 2021). Tyrosine serves as the building compound for the synthesis of the catecholamines norepinephrine, epinephrine and dopamine (Chandel, 2021b), hormones that have a closely relationship with hypertension and CVD. Furthermore, tryptophan serves as a precursor for the production of the neurotransmitter serotonin (Chandel, 2021b), which exerts pro-atherosclerotic effects (Shimabukuro, 2022). Moreover, methionine can have a proatherogenic effect by increasing the production of homocysteine, which has been identified as a risk factor for atherosclerosis (Škovierová et al., 2016). On the other hand, cysteine, glutamate, and glycine contribute to the production of the antioxidant glutathione, which is atheroprotective (Chandel, 2021b; Rom et al., 2022). Also, arginine is required for the synthesis of NO, which is a key protective regulator of vascular homeostasis and immune cell function (Nitz et al., 2019). Furthermore, amino acids are closely linked to key anabolic cell signaling pathways, like the mTOR pathway (O’Neill et al., 2016). mTOR-containing complexes have a function in sensing amino acid levels, and mTOR-driven anabolic growth requires sufficient amino acid availability (Saxton and Sabatini, 2017).

## 4 Macrophage metabolic alterations in the context of inflammation and atherosclerosis

Macrophages contribute to the development, expansion and rupture of atherosclerotic plaques but also support processes of plaque regression (Tabas and Bornfeldt, 2020; Xue S. et al., 2023). Macrophages are heterogeneous and plastic, and dependent on the micro-environment, can change their phenotype. Although a plethora of macrophage phenotypes has been identified by the advent of single cell biology - amongst others resident macrophages, TNF^+^ pro-inflammatory macrophages, IL1β^+^ pro-inflammatory macrophages, Trem2^+^ macrophages, lipid-associated Trem2 hi-perilipin^+^ macrophages, and IL10^+^TNFAIP3^+^ macrophages (de Winther et al., 2023; Dib et al., 2023), little is known about the metabolism of these newly identified subsets. Traditionally, two main categories of macrophage polarization were distinguished, being the classically activated, pro-inflammatory M1 macrophages (which are stimulated by lipopolysaccharide (LPS) and interferon (IFN)-γ and trigger a pro-inflammatory response) and alternatively-activated, anti-inflammatory M2 macrophages (which are stimulated by IL-4 and IL-13 and inhibit inflammatory responses) (Xue S. et al., 2023; O’Rourke et al., 2022; Batista-Gonzalez et al., 2019). Macrophages of the M1 pro-inflammatory phenotype, resembling several of the pro-inflammatory transcriptional macrophage subsets, are increasingly accumulating in atherosclerotic lesions upon progression towards more inflammatory lesions (Khallou-Laschet et al., 2010). Instead, macrophages of the M2 phenotype do not have a clear counterpart in the transcriptional subsets, but its protein markers arginase and mannose receptor are found more in the shoulder regions covering the lipid core region and are less prone to develop into foam cells (Chinetti-Gbaguidi et al., 2011).

The metabolic pathways of glycolysis, the TCA and OXPHOS cycle, the PPP, lipid as well as amino acid metabolism play an important role in macrophage polarization and subsequently their inflammatory phenotype and contribution to atherosclerotic plaques (Koelwyn et al., 2018; Tabas and Bornfeldt, 2020; Xue S. et al., 2023).

**FIGURE 1:**
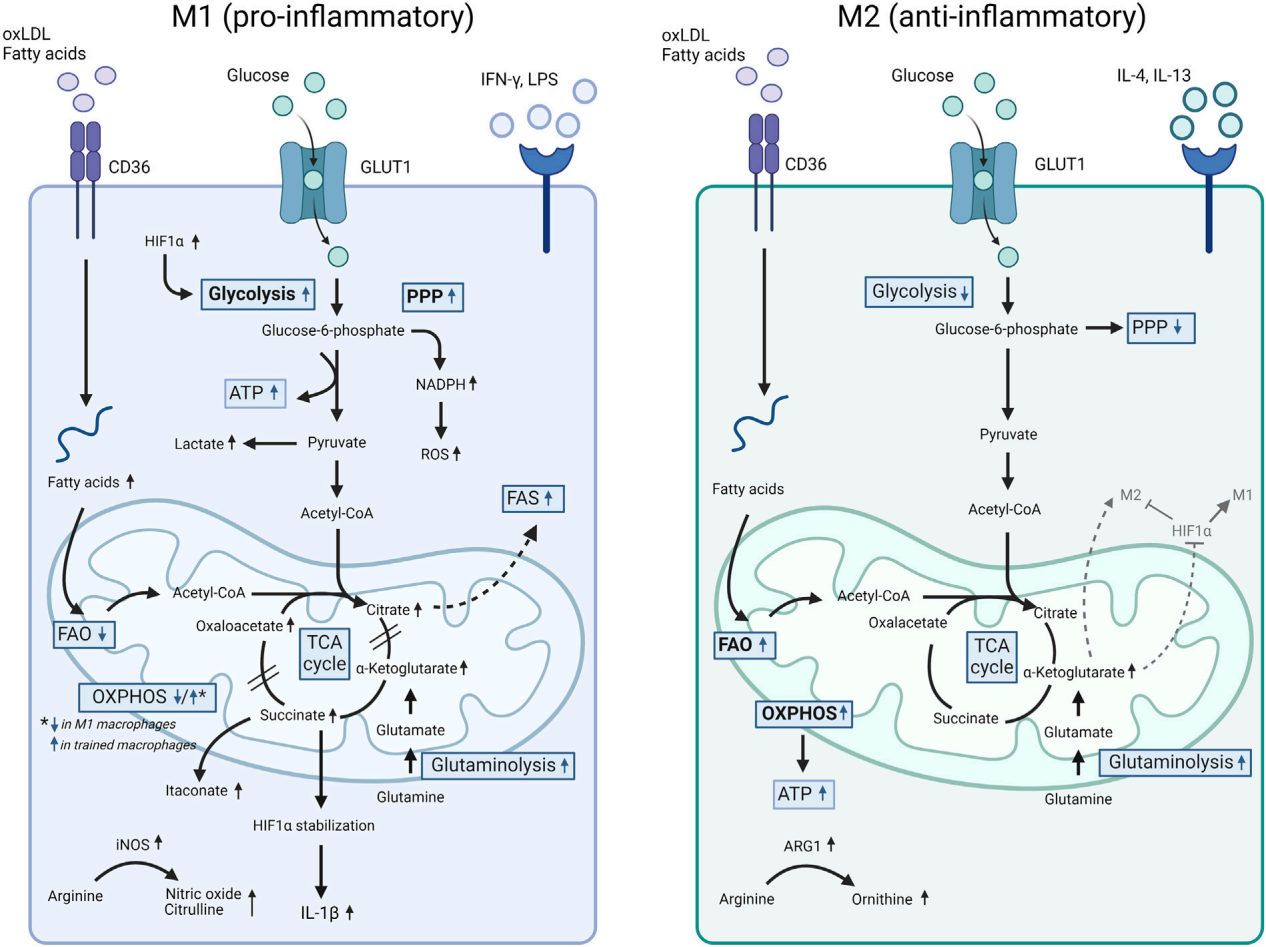
Comparison of cell metabolism in M1 versus M2 macrophages. Pro-inflammatory M1 macrophages (e.g., induced by LPS and oxLDL) show increased glycolysis with enhanced pyruvate to lactate conversion, triggered by ROS production and HIF1α accumulation, as well as an activated PPP. Furthermore, LPS downregulates the OXPHOSP cycle and triggers an accumulation of TCA intermediates. Increased glycolysis and an accumulation of TCA intermediates were also observed in trained monocytes induced by oxLDL and LPS. In these conditions, a simultaneous increase in OXPHOSP as well as increased glutaminolysis was also detected. Finally, fatty acid synthesis (FAS) supports the M1 pro-inflammatory phenotype. In comparison to M1, M2 macrophages downregulate glycolysis and PPP whereas upregulate the FAO and OXPHOSP pathways and show an intact TCA cycle. For more details, we refer to the text. ARG1, arginase 1; ATP, adenosine triphosphate; FAO, fatty acid oxidation; FAS, fatty acid synthesis; GLUT1, glucose transporter 1; HIF1α, hypoxia inducible factor 1-alpha; IFN-γ, interferon gamma; IL, interleukin; iNOS, inducible nitric oxide synthase; LPS, lipopolysaccharide; NADPH, nicotinamide adenine dinucleotide phosphate; oxLDL, oxidized LDL; OXPHOS, oxidative phosphorylation; PPP, pentose-phosphate pathway; ROS, reactive oxygen species; TCA, tricarboxylic acid cycle.

In general, M1-type macrophages rely on glucose metabolism through aerobic glycolysis and the anabolic PPP, whereas M2-type macrophages have increased FAO, rely extensively on the mitochondrial TCA/OXPHOS cycle and show a decreased dependency on the PPP (Rhoads et al., 2017; Koelwyn et al., 2018) (Table 1) (Figure 1).

**TABLE 1:** Metabolic profiles of M1 vs. M2-polarized macrophages.

	M1 (pro-inflammatory)	M2 (anti-inflammatory)
Triggered by	IFN-γ, LPS	IL-4, IL-13
Cytokine production	TNF, IL-6, IL-1β, IL-18, IL-1α	TGF-β, IL-10
Glycolysis	High (with high pyruvate to lactate conversion)	Low
Pentose-phosphate pathway	High	Low
TCA cycle	Interrupted, with accumulation of TCA cycle substrates (e.g., succinate, citrate) and byproducts (e.g., itaconate)	Intact
OXPHOS	Low (but high in trained monocytes/macrophages)	High
Lipid metabolism	Lipotoxicity is due to increased fatty acid uptake but reduced FAO. Increased fatty acid synthesis	Increased FAO
Amino acid metabolism	Arginine to citrulline and NO Glutamine fuels succinate acid production Tryptophan, Serine, Methionine and Aspartate metabolism stimulate M1 polarization Phenylalanine attenuates M1 polarization	Arginine to ornithine Glutamine conversion to α-KG, with high α-KG/succinate ratio facilitating M2 polarization

PPP, pentose-phosphate pathway; OXPHOSP, oxidative phosphorylation; TCA, tricarboxylic acid cycle; IFN-γ, interferon gamma; α-KG, α-ketoglutarate; IL-1β, interleukin-1β; TNF, tumor necrosis factor; IL-4, interleukin-4; IL-6, interleukin-6; IL-1α, interleukin-1α; IL-18, interleukin-18; IL-10, interleukin-10; IL-13, interleukin-13; NO, nitric oxide; LPS, lipopolysaccharide; TGF-β, transforming growth factor-β; FAO, fatty acid oxidation.

### 4.1 Glycolysis, oxidative phosphorylation, and the pentose-phosphate pathway

In general, pro-inflammatory activation of macrophages – for example by pathogen associated molecular patterns (PAMPs) as Toll-like receptor (TLR) stimulation - induces a profound metabolic reorganization that is characterized by an increase in glucose uptake and glycolysis, a disrupted TCA cycle and (depending on the stimulus) a reduction in OXPHOS (Rodríguez-Prados et al., 2010; Jha et al., 2015).

#### 4.1.1 Increased glycolysis in hypoxia, pro-inflammatory conditions and monocyte/macrophage trained immunity

In hypoxic conditions - such as in atherosclerotic lesions with a high content of proliferating and metabolically active cells -, glycolysis is upregulated through increased hypoxia-inducing factor (HIF)1α mediated expression of glycolysis-regulating genes, such as PFKFB3 (Folco et al., 2011; Folco et al., 2014; Tawakol et al., 2015; van der Valk et al., 2015; Dib et al., 2023). However, while acute, systemic silencing of PFKFB3 ameliorated macrophage inflammation and atherosclerosis, chronic myeloid-specific inhibition of PFKFB3 did not have any effects on atherosclerosis (Tawakol et al., 2015; Tillie et al., 2021). In addition to regulating glycolysis, mild hypoxia increases the levels of the NLRP3 inflammasome and – associated with this – drives the production and secretion of pro-inflammatory IL-1β (Folco et al., 2011; Folco et al., 2014), with proven causality of systemic inhibition on human atherosclerosis (Ridker et al., 2017).

However, also in aerobic conditions, activation of the HIF1α pathway upon pro-inflammatory stimulation of macrophages can drive the upregulation of aerobic glycolysis with increased glucose consumption and a glycolytic flux of pyruvate towards lactate production rather than fueling the TCA cycle and mitochondrial respiration, as for example observed upon stimulation with oxLDL (Lee et al., 2014), LPS (Mills et al., 2016) or β-glucan (Cheng et al., 2014).

OxLDL not only promotes the transformation of macrophages to M1, but also inhibits the polarization into M2 macrophages (He and Liu, 2023). Mechanistically, oxLDL triggered the generation of ROS via NADPH-dependent oxidase (NOX)-2, which supported increased glucose uptake through the accumulation of HIF1α triggered by hypoxia (Lee et al., 2014). Also, LPS increased glycolysis in macrophages, coinciding with an increased GLUT1 and hexokinase expression and increased absorption of F-fluorodeoxyglucose (Mills et al., 2016). In parallel, LPS downregulated the OXPHOS cycle and triggered the accumulation of succinate, which contributed to the stabilization of HIF1α and–via succinate oxidation by mitochondrial succinate dehydrogenase–to the production of mitochondrial ROS and subsequent pro-inflammatory gene expression with enhanced production of IL-1β (Mills et al., 2016).

Such metabolic rewiring towards increased aerobic glycolysis also formed the basis for β-glucan-induced trained immunity of monocytes and was associated with epigenetic histone modifications and increased expression of genes involved in glycolysis triggered by the AKT-mTOR-HIF1α pathway (Cheng et al., 2014; Arts et al., 2016). β-Glucan-trained monocytes also showed an upregulation of glutamine utilization and – with glutamine metabolism able to replenish the TCA cycle via α-ketoglutarate (α-KG) – an accumulation of different intermediates of the TCA cycle (succinate, fumarate, malate) (Arts et al., 2016). Fumarate itself was able to trigger HIF1α stabilization and the expression of HIF1α-dependent genes, and in parallel increased histone methylation by inhibiting the activity of the histone demethylase KDM5, thereby triggering increased expression of TNF and IL-6 (Arts et al., 2016). In a comparable way, also oxLDL induces a trained innate immunity in monocytes, i.e., an increased secondary response following an initial first stimulus (Sohrabi et al., 2018; Di Gioia et al., 2020; Keating et al., 2020). Mechanistically, this was found to be triggered by mTOR-mediated ROS production, which increased HIF1α-mediated gene expression and – in line with HIF1α known to drive glycolysis (Cheng et al., 2014) - upregulated glycolysis and a glycolytic flux towards lactate production (Sohrabi et al., 2018). A role for both cytoplasmic and mitochondrial ROS was revealed in the regulation of mTOR-HIF1α, metabolic reprogramming and trained immunity (Sohrabi et al., 2018). Furthermore, oxLDL-induced metabolic rewiring complemented epigenetic histone modifications in oxLDL-primed monocytes, which also contributed to a trained immunity and increased pro-inflammatory cytokine production upon secondary stimulation (Bekkering et al., 2014). In parallel to increased glycolysis, also the OXPHOS cycle was found to be upregulated and required for the trained phenotype in oxLDL-trained monocytes with secondary stimulation with LPS (Keating et al., 2020; Groh et al., 2021). A role for both glutamine conversion to glutamate – which can then feed the TCA cycle – and CPT-1 in oxLDL-trained pro-inflammatory cytokine production was shown, suggesting that glutamine and/or fatty acids could serve as initial substrates towards the OXPHOS cycle (Groh et al., 2021). In line, the antihyperglycemic drug metformin – which activates AMP-activated protein kinase (AMPK) but blocks mTOR signaling (and thereby glycolysis) and which also inhibits the OXPHOS cycle by interfering with the electron transport chain – interfered with both β-glucan- and oxLDL-induced trained immunity of monocytes (Arts et al., 2016; Keating et al., 2020). A simultaneous activation of glycolysis and OXPHOS, together with a dependency on glutaminolysis, an accumulation of TCA intermediates and a hyperactive state was also observed in LPS-primed macrophages with secondary stimulation with oxidized phospholipids (Di Gioia et al., 2020) as well as in oxLDL-primed monocytes with secondary LPS stimulation (Keating et al., 2020). Among substrates that feed the TCA cycle preceding the OXPHOS cycle, α-KG (as end-product of glutamine utilization) and oxaloacetate accumulation enabled a strong IL-1β production (Di Gioia et al., 2020). The other way around, inhibiting glutamine utilization or oxaloacetate production protected macrophages from hyperactivation and reduced atherosclerotic lesion formation *in vivo* (Di Gioia et al., 2020).

Of note, while a central role for HIF1 in macrophage glycolysis can be appreciated from aforementioned, silencing of HIF1α or HIF2α, or exaggerated HIF signaling by knockdown of its upstream regulators has many other cellular effects that complicate its use as intervention in atherosclerosis (Marsch et al., 2014; van Kuijk et al., 2022).

#### 4.1.2 Impact of pyruvate kinase and lactate on macrophage phenotype

Pyruvate kinase is a key enzyme in the glycolysis pathway: it catalyzes the dephosphorylation of phosphoenolpyruvate into pyruvate and is responsible for the production of ATP during glycolysis. Increased glucose absorption and glycolytic flux was observed in monocytes and macrophages from patients with coronary artery disease (CAD), along with a high production of mitochondrial ROS. This altered the conformation state of pyruvate kinase M2 (PKM2) from tetrameric to dimeric and thereby favored its nuclear translocation, where PKM2 phosphorylated the transcription factor signal transducer and activator of transcription 3 (STAT3) to increase pro-inflammatory IL-6 and IL-1β production (Shirai et al., 2016). However, lactic acid as glycolysis byproduct derived from pyruvate, can modulate the macrophage phenotype from a pro-towards an anti-inflammatory M2 direction by inducing the lactylation of PKM2 (Wang et al., 2022). Mechanistically, PKM2 lactylation alters its conformation to a preferential tetrameric over its otherwise dimeric/monomeric form, and thereby reduces PKM2 nuclear accumulation, where it would otherwise drive the expression of proglycolytic genes in interaction with HIF1α (Wang et al., 2022) as well as pro-inflammatory genes (Shirai et al., 2016). Also, in the late phase of M1 macrophage polarization, lactate-derived lactylation of histone lysine residues as observed in conditions of hypoxia and bacterial challenges served as an epigenetic modification that directly stimulated the expression of homeostatic genes that have been traditionally associated with M2-like macrophages (Zhang et al., 2019). Furthermore, through its receptor GPR81, lactic acid can decrease the pro-inflammatory response of LPS-stimulated macrophages by reducing Yes associated protein (YAP), thereby blocking also downstream NF-κB activation and the macrophage pro-inflammatory response signaling (Yang et al., 2020).

#### 4.1.3 The PPP triggers an M1 macrophage phenotype

The activated M1 macrophage phenotype is controlled by the PPP. The PPP is activated in response to LPS and IFN-γ, which increases the production of NADPH in macrophages (Umar et al., 2021). On the one hand, this NADPH production is important for maintaining the redox balance in the cells by producing antioxidants that protect immune cells from ROS. But on the other hand, NADPH supports ROS production via NOX2 and thereby, increased PPP and NADPH contribute to triggering ROS-dependent pro-inflammatory signaling cascades such as NF-κB and MAPK (Dionisio et al., 2023). Furthermore, activated macrophages have increased phagocytic activity, which is supported by their increased PPP activity. Also, PPP-derived NADPH is especially important for FAS and cholesterol metabolism, two processes that are critical to macrophage activity (Koelwyn et al., 2018; Batista-Gonzalez et al., 2019). For example, fatty acids and derived complex lipids are important for membrane remodeling as well as for the production of pro-inflammatory lipid mediators in M1 macrophages (Koelwyn et al., 2018; Batista-Gonzalez et al., 2019). Finally, metabolic reprogramming required for proper M1-vs. M2-like macrophage polarization was also shown to be controlled by CARKL, a sedoheptulose kinase of the PPP: CARKL represses the M1 phenotype and its pro-inflammatory gene expression and ROS production, whereas sensitizing macrophages towards M2 polarization (Haschemi et al., 2012).

### 4.2 Tricarboxylic acid cycle (TCA)

Whereas M2-type macrophages exhibit an intact TCA cycle along with elevated ATP and OXPHOS levels, M1-type macrophages experience disruptions in the TCA cycle at multiple nodes, with trained innate monocytes/macrophages harboring an increased accumulation of TCA cycle intermediates (e.g., succinate, citrate) and byproducts (e.g., itaconate) (Rhoads et al., 2017; Koelwyn et al., 2018; Xu et al., 2024). An accumulation of TCA intermediates may subsequently have an impact on the macrophage phenotype, e.g., by contributing to a pro-inflammatory phenotype [e.g., as shown for succinate (Tannahill et al., 2013) and oxaloacetate (Di Gioia et al., 2020)], fatty acid synthesis for membrane production or pro-inflammatory fatty acid-derivatives as prostaglandin [for citrate (Infantino et al., 2011)] or by triggering innate immune memory [for fumarate (Arts et al., 2016)].

#### 4.2.1 TCA intermediate accumulation in M1 and trained macrophages

More specifically, increased glutamine uptake in LPS-stimulated macrophages triggered the accumulation of succinate, which stabilized HIF1α and thereby increased the expression of the inflammatory mediator IL-1β (Tannahill et al., 2013). Also, mitochondrial oxidation of accumulated succinate triggered ROS production (Mills et al., 2016). Citrate not only provides a bridge between carbohydrate and fatty acid metabolism, but it can also be used to increase fatty acid biosynthesis, resulting in an increased generation of inflammatory prostaglandins (Williams and O’Neill, 2018). In this context, citrate is transported out of the mitochondria into the cytosol via the mitochondrial citrate carrier SLC25a1 and - with the help of ATP citrate lyase – functions as an acetyl donor to support the generation of acetyl CoA and fatty acid-derived derivatives. Citrate-derived acetyl-CoA can also support histone acetylation, with for the latter both pro- as well as anti-inflammatory effects described in – for example - LPS-stimulated macrophages (Ryan and O’Neill, 2020; Lauterbach et al., 2019). While macrophage-specific knockout of ATP citrate lyase stabilized murine plaques and showed dysregulated cholesterol and fatty acid metabolism, main effects seemed to be exerted via changes in apoptosis (Baardman et al., 2020).

Fumarate accumulation in β-glucan trained monocytes increases histone methylation associated with an increased pro-inflammatory gene transcription of TNF and IL-6 (Arts et al., 2016). On the other hand, increased levels of α-KG are used as a cofactor by the histone demethylase lysine demethylase 6B (KDM6B, also known as JMJD3) to promote anti-inflammatory gene expression reminiscent of the macrophage M2 phenotype (Liu et al., 2017). Also, itaconate is increasingly produced by macrophages with a disrupted TCA cycle - as shown upon LPS stimulation (Strelko et al., 2011) - but triggers subsequently protective effects, e.g., by the inhibition of succinate dehydrogenase (which controls levels of pro-inflammatory succinate), glycolysis and pro-inflammatory NLRP3 inflammasome activation (Strelko et al., 2011), whereas activating the anti-inflammatory transcription factors NRF2 and ATF3 (Peace and O’Neill, 2022). *In vivo*, itaconate could suppress atherogenesis by inducing NRF2-dependent inhibition of pro-inflammatory responses in macrophages (Song et al., 2023).

### 4.3 Lipid metabolism

#### 4.3.1 Fatty acid oxidation

FAO supplies FADH_2_ and NADH molecules required for the OXPHOS process, which is considered to be an important process supporting M2 polarization (Van den Bossche et al., 2017). Analyzing the mechanisms of fatty acid metabolism in IL-4-induced murine macrophage polarization, Huang et al. revealed that OXPHOS stimulates the production of genes essential for M2 polarization and raises the spare respiratory capacity of mitochondria (Huang et al., 2014). Besides, IL-4 actives peroxisome proliferator-activated receptor-γ (PPARγ) coactivator-1β (PGC-1β) and STAT6, which results in macrophage mitochondrial biogenesis and FAO (Vats et al., 2006). However, opposite results proposed that FAO is not required for M2 polarization (Namgaladze and Brüne, 2014; Nomura et al., 2016), and others also showed a role of glycolysis in fueling the TCA cycle for mitochondrial respiration in M2-polarized macrophages dependent on the mTORC2 and STAT6 pathways towards IRF4 activation (Huang et al., 2016).

#### 4.3.2 Fatty acid synthesis

FAS has been identified to be necessary for M1 induction (Batista-Gonzalez et al., 2019). Mechanistically, FAS is required for macrophage membrane remodeling: a loss of FAS resulted in modifications in the plasma membrane composition and Rho GTPase trafficking, which attenuated macrophage inflammatory signaling (Wei et al., 2016). Furthermore, the pro-inflammatory macrophage response of NLRP3 inflammasome induction and subsequent release of IL-1β and IL-18 in response to an LPS challenge is also mediated by the activation of FAS, driven by mitochondrial uncoupling protein 2 (UCP2) and FASN triggering AKT signaling required for NLRP3 expression (Moon et al., 2023).

#### 4.3.3 Cellular lipotoxicity and fatty acids

One of the main initiators of macrophage foam cell development and atherosclerosis is the disturbance of regular lipid metabolism in macrophages. Excessive lipid uptake leads to the formation of foamy macrophages, inhibits their migratory capacity and traps them in the intima, ultimately leading to the induction of macrophage apoptosis and amplification of chronic inflammation (Moore et al., 2013). Via the CD36 receptor, oxLDL triggered the cellular accumulation of long-chain fatty acids by increased cellular uptake and mitochondrial import vs. a reduced mitochondrial FAO. This was associated with mitochondrial dysfunction and ROS production as well as pro-inflammatory NF-κB activation and increased cytokine production (Chen et al., 2019).

Lysosomes break down internalized lipoproteins, releasing a lot of free cholesterol and fatty acids (Moore et al., 2013). Transcriptional regulation of cholesterol homeostasis is governed by liver X receptors (LXRα and β) and sterol regulatory element-binding transcription factor 2 (SREBP2). The activation of LXRs, triggered by the accumulation of cholesterol in cells, results in enhanced cholesterol efflux and diminished cholesterol import (Luo et al., 2020). Furthermore, LXR activation raises the generation of lactate and acetyl-CoA, upregulates the expression of SREBP1 and stimulates the mevalonate pathway and IL-1β signaling in human monocytes, thereby contributing to a pro-inflammatory trained immunity phenotype (Sohrabi et al., 2020).

Fatty acids can be taken up by cells and transported to the mitochondria for FAO and ATP production. However, if saturated fatty acids accumulate in excess in cells, they become toxic and induce the generation of ROS, endoplasmic reticulum stress and NLRP3 inflammasome activation, which are all associated with a pro-inflammatory macrophage phenotype (Legrand-Poels et al., 2014). Furthermore, the saturated fatty acid palmitate is highly pro-inflammatory by triggering TLR2- and TLR4 signaling and activating NF-κB, in contrast to the long-chain omega-3 polyunsaturated fatty acid (PUFA) docosahexaenoic acid, which was able to counteract this effect (Huang et al., 2012). Overall, saturated fatty acids have been shown to be more pro-inflammatory and cytotoxic compared to unsaturated fatty acids, caused by their lower efficiency of being esterified in triglycerides for storage in lipid droplets (Legrand-Poels et al., 2014; Soppert et al., 2020; Noels et al., 2021). Nonetheless, also unsaturated fatty acids have been associated with pro-inflammatory responses in macrophages: atherosclerotic lesions from hyperlipidemic mice were shown to be highly abundant in the mono-unsaturated fatty acid oleic acid and the omega-6 PUFAs linoleic acid and arachidonic acid, which – in contrast to the saturated fatty acids palmitic acid or stearic acid - could trigger inflammasome-independent IL-1α production in macrophages. Oleic acid triggered foam cell formation and macrophage IL-1α secretion *in vitro* and the formation of atherosclerosis in mice. Mechanistically, a role of intracellular calcium release and uncoupling of mitochondrial respiration in oleic acid-induced IL-1α secretion was revealed (Freigang et al., 2013).

### 4.4 Amino acid metabolism

In macrophages, amino acid catabolism plays a crucial role in controlling multiple macrophage response pathways - including mTOR signaling and NO generation – and immune characteristics (Kieler et al., 2021).

#### 4.4.1 Glutamine

Glutamine metabolism not only promotes the synthesis of succinic acid by fueling the TCA cycle in M1-type macrophages, but also drives M2 polarization via the conversion to glutamate and then α-KG, with high α-KG levels and a high α-KG/succinate ratio facilitating macrophage reprogramming towards the M2 phenotype (Liu et al., 2017). Mechanistically, α-KG destabilized HIF1α and downregulated inflammation-induced IL-1β production (Tannahill et al., 2013). Also, α-KG downregulated the mTORC1 pathway and instead induced nuclear PPARγ as driver of FAO gene expression (Liu et al., 2017). Furthermore, α-KG triggered metabolic reprogramming to an M2 gene expression profile by promoting JMJD3-dependent histone demethylation in the promoter of M2 marker genes (Liu et al., 2017). Moreover, glutamate can be metabolized to citrate in the TCA cycle. This supports the production of acetyl-CoA, which is then used for the production of cholesterol and fatty acids. Glutamine and glutamate can trigger triglyceride accumulation in macrophages, whereas glycine, alanine, leucine, and cysteine can downregulate triglyceride levels (Rom et al., 2017). Macrophages deficient for glutamate 1 synthase (GLS1) did not show major changes in cell proliferation or cell death, but – surprisingly - induced an M2-like reparative macrophage phenotype. However, the capacity of GLS1-deficient macrophages to clear apoptotic cells (efferocytosis) was dampened, leading to exaggerated atherosclerosis and necrotic core formation (Merlin et al., 2021). Unexpectedly, this was attributed to a non-canonical transaminase pathway, independent from canonical α-KG-dependent immunometabolism (Merlin et al., 2021).

#### 4.4.2 Arginine

Arginine is transformed into citrulline and NO by the M1 marker inducible nitric oxide synthase (iNOS), with NO inhibiting the electron transport chain and thereby mitochondrial respiration (Kieler et al., 2021). Inversely, arginase 1 (ARG1) is constantly expressed by anti-inflammatory M2 macrophages. ARG1 can convert arginine to ornithine, which can enhance macrophage efferocytosis and thereby counteract atherosclerosis (Yurdagul et al., 2020), a process that also involves macrophage autophagy (Liao et al., 2012). Therefore, the M1 vs. M2 phenotype of macrophages is co-determined by the equilibrium in the arginine metabolism by ARG1 vs. iNOS. Of note, this pathway of arginine-induced efferocytosis (with arginine derived from engulfed apoptotic cells) is complemented by efferocytosis-induced glycolysis dependent on activation of the enzyme 6-phosphofructo-2-kinase/fructose-2,6-bisphosphatase 2 (PFKFB2), triggering enhanced binding of apoptotic cells to the macrophage cell surface via glycolysis-derived lactate (Schilperoort et al., 2023).

#### 4.4.3 Tryptophan

Tryptophan was shown to promote macrophage M1 polarization *in vitro*, with breast cancer macrophages revealing a high tryptophan metabolism along with a high M1 gene expression score (Xue L. et al., 2023). Besides, the tryptophan metabolites 3-hydroxy-kynurenine, 3-hydroxyanthranilic acid and quinolinic acid regulate oxidative stress processes, such as lipid peroxidation, and may act both in a pro-oxidative and anti-oxidative way (Nitz et al., 2019).

#### 4.4.4 Methionine

Methionine can induce M1 macrophage polarization, which was linked to a pro-inflammatory state of macrophages with increased TNF production and iNOS activity, alterations in extracellular nucleotide metabolism, and an increased hydrolysis of ATP and ADP (Dos Santos et al., 2017; Franceschi et al., 2020).

#### 4.4.5 Phenylalanine

Phenylalanine has been proposed to attenuate the inflammatory profile of M1 macrophages by reprogramming the transcriptomic and metabolic profiles, thereby augmenting oxidative phosphorylation in M1 macrophages. This reprogramming subsequently mitigated the activation of caspase-1, consequently hindering the production of IL-1β and TNF by M1 macrophages (Zhang et al., 2023).

#### 4.4.6 Serine

Serine metabolism is important for LPS-mediated induction of IL-1β mRNA expression and glutathione synthesis in macrophages (Rodriguez et al., 2019). On the other hand, suppressing serine metabolism increased IGF1 expression, which subsequently triggered the activation of the p38-dependent JAK-STAT1 pathway, promoting macrophage M1 polarization while concurrently inhibiting STAT6-mediated macrophage M2 activation (Shan et al., 2022).

#### 4.4.7 Aspartate

Aspartate can enhance the activation of the inflammasome and HIF1α and promote macrophage M1 polarization (Wang et al., 2021).

### 4.5 Associated effects of atherosclerosis comorbidities on macrophage metabolism

Macrophage polarization changes during many disorders such as obesity, T2DM and hypertension, which are important comorbidities and risk factors for atherosclerosis.

#### 4.5.1 Obesity

Obesity is defined by adipose tissue accumulation by an energy overload (Yao et al., 2022). Numerous M1 macrophages are recruited into the adipose tissue (Yao et al., 2022). Obesity increases adipocyte development and cell hypoxia, which stimulates inflammatory chemokines and cytokines via HIF1α in adipose tissue macrophages (ATMs), inducing their polarization into the M1 phenotype (Fujisaka et al., 2013). Furthermore, increased circulating levels of free fatty acids – as observed in obesity – trigger M1 polarization of ATMs via TLR4 signaling activation (Shi et al., 2006). Increased consumption of free fatty acids by monocytes/macrophages induces a change in fatty acid metabolism from OXPHOS to triglyceride, phospholipid and ceramide production, which in turn enhances lipotoxicity and an M1 phenotype (Namgaladze and Brüne, 2016).

#### 4.5.2 T2DM

In long-term hyperglycemic conditions, macrophages undergo a state of heightened stress, rendering them susceptible to excessive reactivity to external stimuli. This triggers the secretion of excessive inflammatory factors like TNF, IL-6, and CCL2, which are more likely to promote M1 polarization (Wu H. et al., 2021). Matsuura et al. demonstrated in mice that diabetes diminishes the expression of GLUT1 and GAPDH, thereby reducing glucose uptake and glycolysis in peritoneal macrophages (Matsuura et al., 2022). Furthermore, glutamine metabolism is changed in T2DM patients, characterized by a decrease in glutamine and α-KG vs. an increase in succinate, favoring macrophage polarization into the M1 phenotype (Ren et al., 2019). The other way around, M1 macrophages secrete a lot of inflammatory cytokines, such as IL-1β, which lead to insulin resistance in the liver, adipose and musculoskeletal tissues, as well as pancreatic β-cell malfunction (Eguchi and Manabe, 2013).

#### 4.5.3 Hypertension

The activation of the RAAS and the SNS - which are both involved in regulating the salt-water balance and cardiovascular function - is increased in hypertension. Both stimulation of the RAAS (Barhoumi and Todryk, 2023) as well as the SNS (Harwani, 2018) mediates the polarization of macrophages towards an inflammatory phenotype. The other way around, renal denervation - which decreases the activation of SNS and RAAS and thereby attenuates blood pressure - leads to a substantial reduction in M1 macrophages into the medulla of the kidney and an increase in the production of kruppel-like factor-4, which drives macrophage M2 polarization (Liao et al., 2011; Xiao et al., 2015).

## 5 Neutrophil metabolic alterations in the context of inflammation and atherosclerosis

Neutrophils contribute to the development of atherosclerosis and the destabilization of plaques (Soehnlein, 2012). Specifically, the role of neutrophil extracellular traps (NETs) and ROS in causing tissue damage and fostering atherosclerosis and thrombosis has gained a lot of attention over the last years (Josefs et al., 2020).

Neutrophils can influence macrophage behavior, exhibit innate immune memory, and contribute significantly to the progression of atherosclerosis (Sreejit et al., 2022). Proteins released from distinct neutrophil granule subsets (e.g., IFN-γ, S100A9 and HNP1-3) provide guidance for the recruitment and activation of various other immune cells, including monocytes, macrophages and dendritic cell subsets (Soehnlein et al., 2009). Additionally, neutrophils produce lipid mediators through the oxygenation of arachidonic acid, leading to leukotriene A4 and its further processing into the potent chemoattractant leukotriene B4 (Soehnlein, 2012). Recent findings from mouse models of atherosclerosis reveal that neutrophils accumulate in large arteries shortly after the initiation of a high-fat diet (van Leeuwen et al., 2008; Drechsler et al., 2010). The size of the lesions exhibits a positive correlation with the counts of circulating neutrophils and the depletion of neutrophils significantly decreases atherosclerotic lesion size, supporting a causal involvement of neutrophils in the initial stages of atherosclerosis (Drechsler et al., 2010).

**FIGURE 2:**
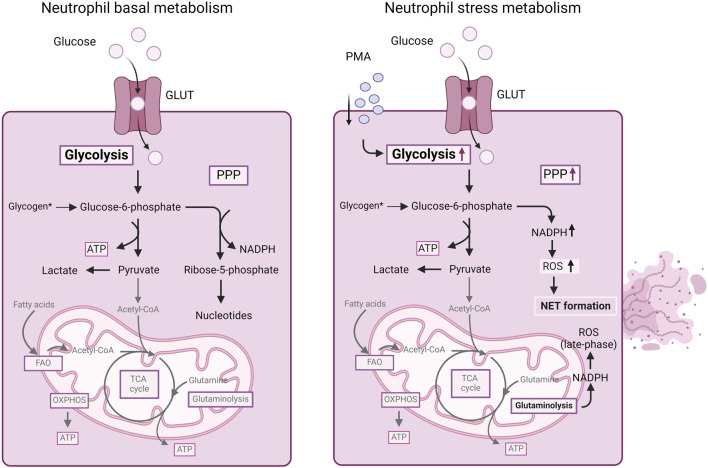
Summary of cellular metabolism in neutrophils. Mature neutrophils mainly depend on glycolysis for energy production. Glucose uptake and glycolysis increases in neutrophils stimulated with PMA. Furthermore, in stressed neutrophils, during an oxidative burst, the PPP is upregulated, allowing the neutrophils to generate NAPDH for redox signaling and ROS production as well as NET formation. Also, NADPH obtained from glutaminolysis can contribute to ROS generation in neutrophils. For more details, we refer to the text. ATP, adenosine triphosphate; FAO, fatty acid oxidation; GLUT, glucose transporter; NADPH, nicotinamide adenine dinucleotide phosphate (reduced form); OXPHOS, oxidative phosphorylation; PMA, phorbol myristate acetate; PPP, pentose-phosphate pathway; ROS, reactive oxygen species; TCA, tricarboxylic acid cycle.

Although in the classical view, neutrophils mainly depend on glycolysis, recent progress in the field of immunometabolism has unveiled that neutrophils engage in diverse metabolic pathways during inflammatory processes (Kumar and Dikshit, 2019). In contrast to other immune cells, where metabolic reprogramming typically leads to differentiation into distinct subtypes, neutrophils adjust their metabolic pathways to carry out various effector functions (Figure 2). These functions include chemotaxis, ROS generation, NET formation and degranulation.

### 5.1 Neutrophil basal metabolism

#### 5.1.1 Glycolysis

Neutrophils primarily rely on glycolysis as the dominant metabolic pathway for generating energy in the form of ATP (Jeon et al., 2020) (Figure 2). Resting neutrophils express glucose transporter proteins (e.g., GLUT1, GLUT3 and GLUT4). Upon activation, there is a notable elevation in the surface expression of these glucose transporters concurrent with an increase in glucose uptake (Rodríguez-Espinosa et al., 2015). Depleting glucose significantly impairs most neutrophil functions (Azevedo et al., 2015; Rodríguez-Espinosa et al., 2015). In contrast, neutrophils, which contain fewer mitochondria than other immune cells, do not heavily rely on the OXPHOS cycle for energy production (Chacko et al., 2013). In the absence of glucose, neutrophils turn to glycogenolysis as an alternative energy source, particularly for phagocytosis (Weisdorf et al., 1982). In inflammatory conditions, neutrophils exhibit heightened glycogen accumulation (Robinson et al., 1982), emphasizing the dominance of glycolysis as an essential metabolic pathway.

#### 5.1.2 Fatty acid oxidation

FAO is mostly used by immature/differentiating neutrophils (Rodríguez-Espinosa et al., 2015). Autophagy - a crucial cellular degradation and recycling process that provides free fatty acids - was demonstrated to support mitochondrial respiration and energy production during neutrophil differentiation in the bone marrow (Riffelmacher et al., 2017). Deficiency of Atg7 (Autophagy related 7 protein) resulted in heightened glycolytic activity but impaired mitochondrial respiration, reduced ATP production as well as lipid droplet accumulation in neutrophil progenitor cells, indicating that during neutrophil development autophagy supports a metabolic shift from glycolysis to FAO and mitochondrial respiration (Riffelmacher et al., 2017). Furthermore, inhibition of FAO resulted not only in reduced neutrophil maturation, but also in the accumulation of lipid droplets (Riffelmacher et al., 2017). In contrast, mature neutrophils primarily utilize glycolysis for energy production, making FAO dispensable. Nonetheless, FAO can mediate neutrophil effector functions. For example, tumor infiltrating neutrophils utilize mitochondrial FAO for ROS generation (Rice et al., 2018). In addition, neutrophils from diabetic rats with impaired glucose and glutamine metabolism presented increased FAO (analyzed as palmitic acid oxidation) in a compensatory mechanism of ATP generation (Alba-Loureiro et al., 2006).

#### 5.1.3 Pentose phosphate pathway

Utilizing both oxidative and non-oxidative phases of the PPP, neutrophils utilize glucose-6-phosphate (an intermediate of the glycolytic pathway) as a precursor. The PPP generates NADPH and ribose- 5-phosphate, which are then utilized for the production of nucleotides (Stanton, 2012). In stressed neutrophils, during an oxidative burst, glycolysis is shifted to the PPP, allowing the neutrophils to promptly mount a first line of defense against pathogens, using the generated NADPH for redox signaling (Britt et al., 2022).

#### 5.1.4 Oxidative phosphorylation

Despite possessing intact mitochondria and a functional TCA cycle, mature neutrophils exhibit minimal reliance on mitochondrial respiration for their energy production (Rodríguez-Espinosa et al., 2015), since the inhibition of mitochondrial respiration had no impact on ATP generation in mature neutrophils (Maianski et al., 2004). Instead, immature neutrophils rely on OXPHOS and FAO, with their energy production depending on degradation of lipid droplets through lipophagy with the resultant fatty acids directed to the TCA and OXPHOS (Riffelmacher et al., 2017).

### 5.2 Neutrophil extracellular trap (NET) formation

#### 5.2.1 NET formation in atherosclerosis and thrombosis

NETs serve a crucial role in the immune system, neutralizing pathogens (Brinkmann et al., 2004). Apart from their function as a host defense mechanism, NETs also play a crucial role in non-infectious contexts, including in CVD [e.g., atherosclerosis (Warnatsch et al., 2015)] and thrombosis (Fuchs et al., 2010) as well as in pathologies enhancing CVD risk, such as diabetes (Wong et al., 2015). In this context, neutrophils expel nuclear DNA associated with histones and proteins, which organize in extracellular web-like structures called traps, in a process called NET formation (Brinkmann et al., 2004). NET-associated granule proteins include MPO (myeloperoxidase), NE (neutrophil elastase), defensins, calprotectin, cathelicidins, cathepsin G, lactoferrin, matrix metalloproteinase-9, peptidoglycan recognition proteins, pentraxin and LL-37. Incorporated histones include H1, H2A, H2B, H3 and H4 (Brinkmann et al., 2004; Hirose et al., 2014; Lim et al., 2018). During NET formation, calcium fluxes activate the enzyme peptidyl arginine deaminase 4 (PAD4), which converts the positively charged amino acid arginine to the neutral citrulline on histones. This impacts the interaction of histones with the negatively charged DNA and triggers DNA unwinding, nuclear expansion and chromatin condensation. In this context, pro-inflammatory cytokines (e.g., TNF, IL-1β, IL-8) can activate NET formation (Brinkmann et al., 2004).

In the context of CVD, NETs not only exert cytotoxic and prothrombotic effects (Warnatsch et al., 2015), but also contribute to plaque formation (Silvestre-Roig et al., 2019). First, NETs have been identified in the luminal area of murine and human atherosclerotic lesions (Megens et al., 2012; Silvestre-Roig et al., 2019). NETs damage the endothelium and induce its activation, leading to an increased expression of adhesion molecules and secretion of tissue factor (TF), which aggravates thrombosis risk. Additionally, NETs interact with von Willebrand Factor (vWF) and factor XII (FXII), promoting thrombus formation and initiating the intrinsic coagulation pathway, respectively (Grässle et al., 2014; Badimon and Vilahur, 2015; Folco et al., 2018).

Mergens et al. reported adhered neutrophils excreting NETs in 57% of the atherosclerotic lesions of mice fed with a high-fat diet vs. 0% of control mice (Megens et al., 2012). In addition, Pertriwi et al. reported an increased presence of neutrophils and NETs particularly in autopsy-derived plaques exhibiting ruptures, superficial plaque erosions or intraplaque hemorrhages (Pertiwi et al., 2018). Furthermore, intimal-delivered NET-associated histones induce necrosis of SMCs and damage to vascular tissues, ultimately contributing to plaque destabilization (Silvestre-Roig et al., 2019). Moreover, NETs trigger the AIM (absent in melanoma 2) inflammasome in macrophages, facilitating the release of the pro-atherogenic cytokines IL-1β and IL-18, which contributes to the development of unstable atherosclerotic lesions (Paulin et al., 2018). Not only was the depletion of neutrophils shown to attenuate lesion formation (Zernecke et al., 2008), but also the inhibition of NET formation was able to preserve plaque stability in mouse models (Silvestre-Roig et al., 2019).

When observing CAD patients, markers of NET formation (e.g., double stranded DNA (dsDNA), nucleosomes, and MPO–DNA complexes) were increased in plasma (Borissoff et al., 2013). Borissoff et al. observed that nucleosomes can potentially predict severe coronary stenosis; that dsDNA was increased in patients with severe CAD or coronary artery calcification; that circulating dsDNA, nucleosomes and MPO–DNA complexes were positively associated with luminal stenosis, and that these markers of NET formation were predictive indicators for major adverse cardiovascular events (MACE) (Borissoff et al., 2013).

#### 5.2.2 Neutrophil metabolism during NET formation

Glycolysis serves as the primary energy source for neutrophils during NET formation (Rodríguez-Espinosa et al., 2015; Behnen et al., 2017). Rodríguez-Espinosa et al., 2015. observed that the metabolic progression of NET formation could be delineated into two stages: the initial phase, characterized by chromatin decondensation, remains unaffected by exogenous glucose; and the later phase involving NET release is strictly contingent upon exogenous glucose availability and glycolysis (Rodríguez-Espinosa et al., 2015). Increased GLUT-1 surface expression and glucose uptake were detected in healthy human neutrophils stimulated with PMA. Glucose deprivation together with glycolysis inhibition (using 2-deoxy-glucose, 2-DG) resulted in the inhibition of NET formation (compared to untreated PMA-stimulated neutrophils), while the addition of glucose alone maintained NET formation in the same conditions (Rodríguez-Espinosa et al., 2015) (Table 2).

**TABLE 2:** Metabolism of neutrophils during NET formation.

References	Cells	Treatment/condition	Effect on metabolism	Associated effect	NET formation	Readouts	PMID
Rodríguez-Espinosa et al. (2015)	Human healthy neutrophils	PMA	↑ Glycolysis	↑ GLUT-1 ↑ Glucose uptake	↑	NET formation is dependent on glucose. Glucose-free medium inhibited NETs	25545227
		Glucose deprivation, glycolysis inhibitor	↓ Glycolysis		↓*		
Behnen et al. (2017)	Human healthy neutrophils	PMA	↑ Glycolysis		↑	NET formation depends on glycolysis	28293240
Azevedo et al. (2015)	Human healthy neutrophils	PMA	↑ PPP	PPP provides NADPH for ROS generation	↑	Shift to PPP is necessary for NETs release	26198639
		Glucose deprivation, PPP inhibitor	↓ PPP	↓ NOX activity	↓*		
Awasthi et al. (2019)	Human healthy neutrophils	PMA, calcium ionophore, lactate	↑ Glycolysis	↑ Lactate	↑	Lactate induced NET formation, while inhibition of LDH activity significantly reduced NET formation by NOX-dependent and -independent mechanisms	31473341
		Lactate dehydrogenase (LDH) inhibitor		↓ Lactate	↓*		
Awasthi et al. (2023)	Human healthy neutrophils	PMA	↑ PPP; ↑ Glutathione metabolism; ↑ Glutamic acid metabolism	ROS generation and apoptosis	↑	Metabolic reprogramming from aerobic glycolysis to PPP in NETting cells (stimulated with PMA)	36265832
Tambralli et al. (2024)	Human healthy neutrophils	PMA		↑ ECAR and OCR (peak)	↑	Inhibiting either glycolysis or the PPP tempered PMA and APS IgG-induced NET formation, but not NET formation triggered by Ca ionophore	38869951
		Ca ionophore A23187 (Ca iono)		Only direct, short peak in ECAR and OCR	↑		
		Antiphospholipid syndrome (APS) IgG *(*vs. *control IgG)*	↑ Glycolysis and PPP	Slow, persistent ↑ in ECAR/OCR; ↑ Lactate, G6P (after 1 h), NAPDH (after 1 and 2 h) and intracellular glycogen	↑		
		2-DG (glycolysis inhibitor)	↓ Glycolysis ↓ PPP	↓ Lactate	↓ (for PMA and APS IgG induced); no difference for Ca iono iono		
		G6PDi-1 (G6PD inhibitor)	↓ PPP	↓ G6PD activity			
		DPI (NOX inhibitor)		↓ Total ROS			
		GPI (Glycogenolysis inhibitor); *in glucose-free media*					

* = vs. stimulated cells without inhibition of the metabolic pathway. G6PD, glucose-6-phosphate dehydrogenase; GLUT-1, glucose transporter 1; NET, neutrophil extracellular traps; NOX, NADPH oxidase; PMA, phorbol myristate acetate; PPP, pentose phosphate pathway; ROS, reactive oxygen species.

In addition, Azevedo et al. unveiled a metabolic shift towards the PPP during NET formation (Azevedo et al., 2015). When inhibiting the PPP with 6-aminonicotinamide (6-AN), a glucose-6-phosphate dehydrogenase (G6PD) inhibitor or when performing glucose deprivation, PMA-induced NET formation was blocked, mainly by the reduction of NADPH oxidase activity (Azevedo et al., 2015). Furthermore, Awasthi et al. observed that human neutrophils exposed to lactate presented increased NET formation and that inhibition of lactate dehydrogenase (LDH) inhibited lactate-induced NET formation (Awasthi et al., 2019) (Table 2). More recently, the same group observed a metabolic reprogramming from aerobic glycolysis to PPP in netting neutrophils (when stimulated with PMA) (Awasthi et al., 2023). Fueled by metabolomics data, numerous metabolic pathways were observed to undergo alterations, encompassing intermediates in carbohydrate metabolism, redox-related metabolites, nucleic acid metabolism, and amino acid metabolism. Enrichment analysis of the detected metabolites revealed increased significance of the PPP and glutathione metabolism in PMA-induced netting neutrophils (Awasthi et al., 2023) (Table 2). Overall, mostly PMA-induced metabolic changes have been studied in relation to NET formation, whereas the impact of physiological stimuli on neutrophil metabolism remains mostly uninvestigated. A recent study by Tambralli et al. revealed that antibodies associated with the thromboinflammatory autoimmune disease antiphospholipid syndrome also triggered NET formation via glycolysis, the PPP and NADPH-dependent ROS production (Table 2) (Tambralli et al., 2024). However, further insights into the relation neutrophil metabolism-NET formation upon physiological stimuli relevant for atherosclerosis remain to be revealed.

### 5.3 ROS production

#### 5.3.1 Neutrophil-derived ROS in atherosclerosis

Within neutrophils, three protein components (MPO, NADPH oxidase, and lactoferrin) participate in redox reactions, capable of generating ROS. The respiratory burst in neutrophils is characterized by an elevated production of the superoxide anion radical (O_2_^•−^), a principal ROS generated in response to external stimuli, e.g., inflammation (Chistiakov et al., 2015).

In the context of atherosclerosis, various factors can induce the respiratory burst, including pro-inflammatory cytokines, lipids such as cholesterol, oxLDL, acute phase proteins like CRP, growth factors such as granulocyte-macrophage colony-stimulating factor (GM-CSF), circulating immune complexes, and others (Ferrante et al., 1988; Kannan et al., 2007). Studies revealed that the O_2_^·−^ production by neutrophils (and other phagocytes) not only promotes oxidative stress in the phagocytes but also in adjacent cells in the vessel wall (Dinauer et al., 1990; Cahilly et al., 2000).

In addition, activated by atherosclerosis-relevant cytokines, MPO is released by neutrophils into the extracellular matrix (Singh et al., 2009). MPO converts chloride anions and hydrogen peroxide into hypochlorous acid, a potent oxidant and chlorinating species, which modifies plaque proteins, colocalizing with MPO (Hazell et al., 1996). Elevated serum levels of MPO predict future cardiovascular events in patients with acute coronary syndrome (Baldus et al., 2003) as well as development of CAD in healthy individuals (Meuwese et al., 2007).

Moreover, experimental findings suggest that neutrophil ROS and proteases contribute to superficial erosion in vulnerable plaques (Hosokawa et al., 2011). There is a detrimental impact of neutrophil-released ROS on ECs, which contributes to plaque formation and inflammation, inducing e.g., vascular hyperpermeability (Meegan et al., 2017). This mechanism could potentially lead to erosion and the disruption of the endothelial cell layer in the advanced stage of atherosclerosis.

#### 5.3.2 Neutrophil metabolism during ROS production

Glycolysis supplies ATP, while the PPP pathway provides NADPH, essential for ROS generation in neutrophils (Petty et al., 2005; Baillet et al., 2017). Additionally, NADPH generated via mitochondrial glutaminolysis contributes to ROS production (Furukawa et al., 2000). Baillet et al. observed that neutrophils activated by PMA exhibit heightened phosphorylation of PFK-2, a pivotal enzyme in glycolysis. Inhibiting this enzyme resulted in reduced glycolysis rates and diminished NADPH oxidase activity in neutrophils (Baillet et al., 2017) (Table 3).

**TABLE 3:** Metabolism of neutrophils during ROS production.

References	Cells	Treatment/condition	Effect on metabolism	Associated effect	ROS	Readouts	PMID
Baillet et al. (2017)	Human healthy neutrophils and PLB985 cells	PMA, fMLP	↑ Glycolysis	↑ pPFK-2	↑	Stimulation leads to an increase of the glycolysis rate and PFK-2 inhibition prevents both hyperglycolysis, leading to a decrease in ATP concentration and NADPH oxidase activation	27799347
		PFK-2 inhibitor	↓ Glycolysis	↓ NADPH oxidase	↓*		
Petty et al. (2005)	Human healthy neutrophils (pregnant or not)	Glucose	↑ PPP		↑ (and ↑ NO)	Neutrophils mainly use the PPP for ROS generation in high-glucose conditions. The inhibition of PPP and NOX attenuates high glucose-induced ROS generation in neutrophils	16390806
		PPP and NAD(P)H oxidase (NOX) inhibitor	↓ PPP (by PPP inhibitor)		↓*		
Britt et al. (2022)	Human healthy neutrophils and HL-60 cells	Zymozan, TNFα, fMLP and PMA	↑ PPP and glycolysis mediators	↑ NOX-dependent OCR	↑	Neutrophil stimulation causes rapid (10 or 30 min) metabolic changes. Activated neutrophils shift to PPP to increase NADPH production for oxidative burst and other effector functions	35347316
		PPP and NOX inhibitors	↓ PPP (by PPP inhibitor) no effect on glycolysis	↓ NOX-dependent OCR	↓*		
Furukawa et al. (2000)	Neutrophils from patients undergoing major gastrointestinal surgery	Basal	↓ Glutaminolysis	↓ Phagocytosis	↓	Glutamine supplementation enhances phagocytosis and production of reactive oxygen intermediates in patients that underwent major gastrointestinal surgery	10793298
		Glutamine	↑ Glutaminolysis	↑ Phagocytosis	↑		
Rice et al. (2018)	Mouse bone marrow-derived neutrophils	PMA		↑ OCR	↑ (↑ H_2_O_2_)	ROS production requires two distinct metabolic pathways, with glucose metabolism required for early phase ROS and mitochondrial function only facilitating the late phase. Neutrophils adapt to glucose-limited environments by using mitochondrial FAO for ROS production via NADPH oxidase	30504842
		Glycolysis inhibitor		↓ early-phase OCR	↓ (↓ H_2_O_2_)*		
		Mitochondrial respiration inhibition		↓ late-phase OCR	↓ (↓ H_2_O_2_) in late phase*		
		FAO inhibition or mitochondrial respiration inhibition in conditions of glucose usage blockade		↓ OCR	↓ (↓ H_2_O_2_)*		
Tambralli et al. (2024)	Human healthy neutrophils	PMA			↑	Inhibiting either glycolysis or the PPP tempered PMA and APS IgG-induced ROS production	38869951
		Ca ionophore A23187 (Ca iono)			No effect		
		Antiphospholipid syndrome (APS) IgG *(*vs. *control IgG)*	↑ Glycolysis and PPP	Slow and persistent ↑ in ECAR/OCR; ↑ Lactate, G6P (after 1 h), NAPDH (after 1 and 2 h) and intracellular glycogen	↑		
		2-DG (glycolysis inhibitor)	↓ Glycolysis ↓ PPP	↓ Lactate	↓ H_2_O_2_ (for PMA and APS IgG induced)		
		G6PDi-1 (G6PD inhibitor)	↓ PPP	↓ G6PD activity			
		DPI (NOX inhibitor)					
		GPI (Glycogenolysis inhibitor)*; in glucose-free media*					

* = vs. stimulated cells without inhibition of the metabolic pathway. FAO, fatty acid oxidation; fMLP, N-Formyl-methionyl-leucyl-phenylalanine; G6PD, glucose-6-phosphate dehydrogenase; OCR, oxygen consumption rate; PFK-2, phosphofructokinase-2; PMA, phorbol myristate acetate; PPP, pentose phosphate pathway; ROS, reactive oxygen species; NOX, NADPH oxidase; TNF, tumor necrosis factor.

Moreover, Petty et al., observed that neutrophils under high glucose stimulation mainly use the PPP for ROS generation, with the inhibition of PPP or NOX attenuating high glucose-induced ROS generation in neutrophils (Petty et al., 2005) (Table 3). Similar effects were observed by Britt et al. when cells were stimulated with zymozan, TNF, fMLP or PMA. The treatments increased PPP and glycolysis mediators, and when the PPP pathway and NOX were inhibited, the neutrophils’ oxygen consumption rate as readout of the neutrophil oxidative burst was reduced (Britt et al., 2022). A role for the PPP and NOX in PMA-induced ROS formation was confirmed by Tambralli et al., with a similar role for these pathways revealed in neutrophil ROS production triggered by antiphospholipid syndrome-associated antibodies (Tambralli et al., 2024) (Table 3).

Although the PPP provides the majority of cellular NADPH, a substantial amount of NADPH can also be obtained by glutaminolysis, and Furukawa et al. revealed that glutamine supplementation boosted ROS generation in neutrophils from patients that had undergone a major gastrointestinal surgery (Furukawa et al., 2000). Despite being dispensable for energy production, mitochondria in neutrophils remain functionally intact, suggesting that neutrophils might opt for the mitochondrial glutaminolysis pathway as an alternative means of supplementing NADPH during ROS generation (Furukawa et al., 2000) (Table 3).

Rice et al. demonstrated that the inhibition of glycolysis strongly reduced the early phase of the oxygen consumption rate (as readout of the oxidative burst) and ROS release in mouse bone marrow-derived neutrophils stimulated with PMA, while inhibition of mitochondrial respiration impacted to a small degree late-phase ROS release (Furukawa et al., 2000; Rice et al., 2018) (Table 3).

### 5.4 Associated effects of atherosclerosis comorbidities on neutrophil metabolism

Neutrophils exhibit metabolic reprogramming during metabolic disorders such as obesity and T2DM as important comorbidities and risk factors of atherosclerosis and CVD.

#### 5.4.1 Obesity

Cichon et al. demonstrated that glycolysis and/or the PPP play a role in NETs release by neutrophils from mice on normal diet in both physiological and inflammatory conditions. In contrast, neutrophils from septic mice on high-fat diet utilize these pathways primarily for spontaneous NET release, whereas upon secondary *ex vivo* activation, they exhibit an “exhausted phenotype,” characterized by diminished NET release, despite maintaining a high glycolytic potential and flexibility to switch to oxidize fatty acids in specific context (Table 4).

**TABLE 4:** Impact of obesity and diabetes on neutrophil metabolism and function.

References	AS comorbidity	Cells	Cells origin	Treatment/condition	Effect on neutrophil metabolism	Associated effect on neutrophil function	PMID
Cichon et al. (2021)	Obesity	Mouse primary neutrophils	Obese mice	HFD	↓ GLUT1 expression ↓ Glycolysis (tendency)	↓ NET formation after 1 h LPS stimulation ↑ NET formation after 6 h LPS stimulation	34299338
				HFD and sepsis	↑ Glycolysis (tendency)	↑ Spontaneous NET formation ↓ NET formation after LPS stimulation	
Joshi et al. (2020)	Diabetes	Human primary neutrophils	Healthy donors	High glucose	↑ Polyol pathway intermediates (NADPH-dependent formation of 1-anhydrosorbitol via aldose reductase) ↓ Glutathione metabolism (with NADPH required for glutathione synthesis)	↑ Cytosolic ROS ↑ Neutrophil elastase secretion ↑ spontaneous NADPH oxidase-dependent NET formation but ↓ LPS-induced NET formation	32827651
				High glucose + Aldose reductase inhibitor		No high glucose-induced increase in cytosolic ROS, neutrophil elastase secretion or spontaneous NET formation, but restored responsiveness to LPS-stimulated NET formation	
Alba-Loureiro et al. (2006)	Diabetes	Rat primary neutrophils	STZ-treated rats		↓ Metabolism of glucose and glutamine ↓ Lactate production and PPP activity	↓ Phagocytosis ↑ Production of H_2_O_2_	16461555

AS, atherosclerosis; GLUT-1, glucose transporter 1; HFD, high-fat diet; LPS, lipopolysaccharide; PPP, pentose phosphate pathway; STZ, streptozotocin; ROS, reactive oxygen species.

#### 5.4.2 T2DM

Many functions of neutrophils, particularly ROS generation, NET formation, bactericidal activity, and chemotaxis, are dysregulated in patients with T2DM (Delamaire et al., 1997; Omori et al., 2008; Wong et al., 2015). Joshi et al., observed that in patients with T2DM, as well as in healthy donor neutrophils exposed to high glucose levels, neutrophil functions compete for NADPH. This resulted in increased cytosolic ROS production upon high glucose treatment, but insufficient NADPH availability for NET production in response to LPS. Notably, supplementing NADPH and using a pharmacological inhibitor of aldose reductase – an enzyme using NADPH oxidase for the formation of sorbitol in high-glucose conditions - successfully restored the sensitivity to LPS-induced NET formation in high glucose conditions, while reducing high glucose-induced cytosolic ROS production, neutrophil elastase secretion and spontaneous NET release (Joshi et al., 2020) (Table 4). Furthermore, in the context of hyperglycemia, Alba-Loureiro, et al. proposed that neutrophils may initiate compensatory FAO due to impaired glucose and glutamine metabolism as well as reduced lactate production and diminished PPP activity, which overall was associated with impaired phagocytosis and H_2_O_2_ production (Alba-Loureiro et al., 2006).

## 6 Metabolic alterations in endothelial cells in the context of inflammation and atherosclerosis

**FIGURE 3:**
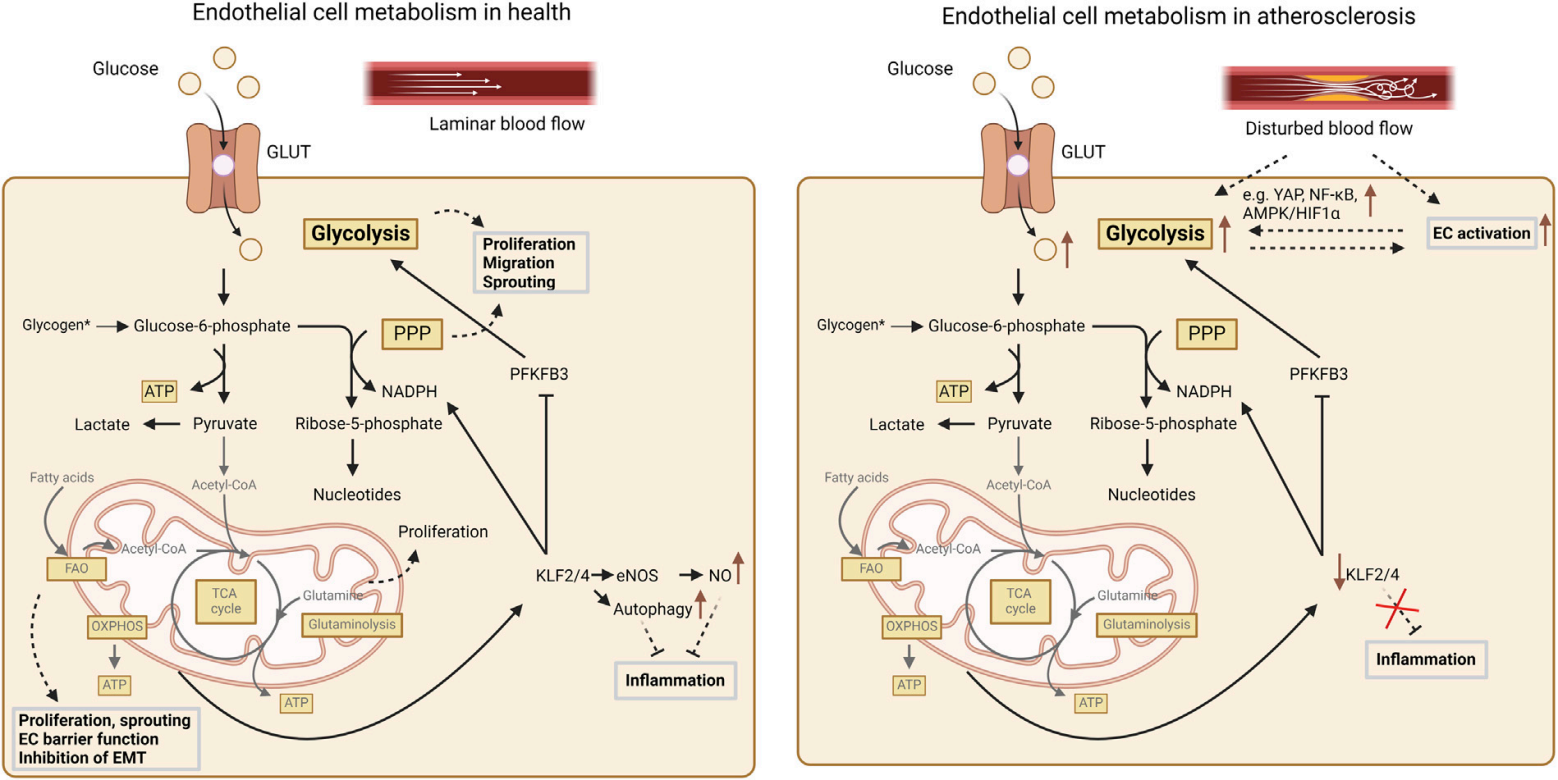
Summary of cellular metabolism in endothelial cells. In healthy conditions glycolysis accounts for ∼80% of the total energy production in endothelial cells. Furthermore, glycolysis as well as the PPP support EC proliferation, migration and sprouting. Mitochondrial oxidative pathways and FAO are less important for energy production but contribute to biomass synthesis. FAO also supports endothelial proliferation, maintenance of the EC barrier function and EC sprouting and furthermore counteracts endothelial-to-mesenchymal transition (EMT). Additionally, FAO also stimulates KLF2/4 expression, which not only promotes NO production but also NADPH production, while it inhibits PFKFB3. Finally, glutamine contributes to EC proliferation, whereas glutamine starvation triggers endoplasmic reticulum stress and inflammation. Endothelial activation and disturbed flow – as in the context of atherosclerosis – are associated with reduced KLF2/4 expression, which coincides with increased glycolysis, which on its turn further contributes to endothelial inflammation. For more details, we refer to the text. AMPK, AMP-activated protein kinase; ATP, adenosine triphosphate; CoA, acetyl-coenzyme A; EC, endothelial cell; eNOS, endothelial nitric oxide synthase; FAO, fatty acid oxidation; GLUT, glucose transporter; HIF, hypoxia inducible factor; KLF, kruppel-like factor; NADPH, nicotinamide adenine dinucleotide phosphate; NO, nitric oxide; OXPHOS, oxidative phosphorylation; PFKFB3, 6-phosphofructo-2-kinase/fructose-2,6-bisphosphatase-3; PPP, pentose-phosphate pathway; TCA, tricarboxylic acid cycle; YAP, yes associated protein.

ECs are the lining between the circulation and the vessel wall and thereby play a crucial role in the regulation of leukocyte recruitment. Activation and dysfunction of ECs is one of the first signs of initial atherosclerosis formation, occurring particularly at bends and branch points in the vasculature (Hajra et al., 2000; Tabas et al., 2015). These dysfunctional ECs are characterized by a pro-inflammatory phenotype with reduced barrier function, leading to the infiltration of LDL into the vessel wall and further activation of the endothelium. Although the endothelium was originally believed to be an inert barrier, it is now viewed as a metabolically active organ playing a crucial role in vascular homeostasis and pathologies (Theodorou and Boon, 2018; Stroope et al., 2024) (Figure 3).

### 6.1 Endothelial cell metabolism in health

#### 6.1.1 Glycolysis

The main energy source accounting for around 80% of the total ATP production in ECs is glycolysis (De Bock et al., 2013) (Figure 3). Such predominant anaerobic metabolism may safeguard the ECs against ROS production (Li et al., 2019). Further indicating the essential need for glycolysis is the notion that inhibition of glycolysis by 2-deoxy-D-glucose induces cytotoxicity in ECs (Merchan et al., 2010). Slight reductions of glycolysis in ECs have also been shown to reduce its proliferation, migration and sprouting capacity (De Bock et al., 2013; Schoors et al., 2014; Yu et al., 2017), while glycolysis is increased by EC stimulation with vascular endothelial growth factor (VEGF) and fibroblast growth factor 2 (FGF2) inducing cellular proliferation and migration (De Bock et al., 2013; Yu et al., 2017). It could be shown that glycolysis in ECs is mainly regulated by PFKFB3 (De Bock et al., 2013). This could also be validated *in vivo* in an EC-specific *Pfkfb3*-deficient mouse model, demonstrating that the lack of *Pfkfb3* results in decreased branch points and sprouts in a postnatal retina model (De Bock et al., 2013). Additionally, PKM2 seems to play an important role in EC function and angiogenesis as silencing of PKM2 resulted in decreased sprouting (Gómez-Escudero et al., 2019), but also in reduced VE-cadherin expression resulting in destabilization of the cellular junctions (Gómez-Escudero et al., 2019).

#### 6.1.2 Pentose phosphate pathway

Besides the use of glucose as energy source, it can also be phosphorylated by hexokinase 2 to generate glucose-6-phosphate, which subsequently can be stored as glycogen or further processed in the PPP to yield NADPH (Mann et al., 2003; Lunt and Vander Heiden, 2011). Also, this pathway has cellular effects as silencing of the G6PD enzyme–the first and rate-limiting enzyme of the PPP – reduces EC proliferation and migration while increasing intracellular ROS (Leopold et al., 2003). It remains however to be determined what the effect of the glycogen metabolism is on EC function.

#### 6.1.3 Fatty acid metabolism

ECs only have a relatively low mitochondrial content (<2–12% of cellular volume) compared to other more oxidative cell types and therefore also exert a lower mitochondrial respiration (Barth et al., 1992; De Bock et al., 2013). However, ECs have a relatively large spare respiratory capacity, which might enable them to utilize fatty acids and glutamine for example, as alternative energy source in stress conditions (Krützfeldt et al., 1990; Wilhelm et al., 2016). Since ECs only derive around 15% of their ATP via oxidative pathways, the mitochondria in ECs seem to play a more important role in biomass synthesis rather than energy production (Schoors et al., 2015). For example, fatty acids are used for the generation of the amino acid aspartate, which is a nucleotide precursor as well as for deoxynucleotides, required for DNA synthesis (Schoors et al., 2015). Additionally, fatty acid metabolism also plays a role in EC functionality as *Cpt1* deficiency resulted in defects in vascular sprouting due to reduced EC proliferation (Schoors et al., 2015) as well as to increased endothelial permeability (Patella et al., 2015). FAO also plays an important role in maintaining the endothelial identity by maintaining the pool of acetyl-CoA and therefore reducing transforming growth factor β (TGF-β)-induced endothelial-to-mesenchymal transition (EndMT) (Xiong et al., 2018).

In addition to these intracellular effects, ECs also regulate the transport of fatty acids toward metabolically active tissues (Mehrotra et al., 2014). Circulating fatty acids can enter the ECs either by passive diffusion or by transporter proteins and subsequently either be stored in lipid droplets to protect against ER stress or released again to the underlying tissues (Kuo et al., 2017).

#### 6.1.4 Amino acid metabolism

The most highly consumed amino acid in ECs is glutamine, which plays a crucial role in angiogenesis (Huang et al., 2017). The lack of glutamine or inhibition of glutaminase 1, the rate-limiting enzyme in glutaminolysis, reduces EC proliferation, while its role in migration remains controversial (Huang et al., 2017; Kim et al., 2017). Methionine and cysteine have also been shown to be involved in angiogenesis as restrictions of these amino acids promoted VEGF production thereby triggering angiogenesis (Longchamp et al., 2018). Furthermore, arginine can be converted to citrulline and NO, which has a wide range of effects on ECs and plays a key role in maintaining vascular homeostasis, by for example suppressing thrombosis, inflammation, and oxidative stress (Tousoulis et al., 2012; Theodorou and Boon, 2018). Valine is another amino acid that influences ECs as it can be converted into 3-hydroisobutyrate (3-HIB), thereby promoting transendothelial fatty acid transport (Jang et al., 2016).

### 6.2 Endothelial cell metabolism in atherosclerosis

In physiological conditions, ECs remain rather quiescent (Gimbrone and Garcia-Cardena, 2016). At sites of undisturbed flow, Kruppel-like factor (KLF) 2 and 4 are expressed driven by FAO, driving the expression of endothelial nitric oxide synthase (eNOS) to generate NO (Atkins and Jain, 2007). This in turn suppresses inflammatory pathways intracellularly (Atkins and Jain, 2007) (Figure 3). Furthermore, KLF2 drives autophagy (Dabravolski et al., 2022), including in endothelial cells (Guixé-Muntet et al., 2017) in which autophagy counteracts endothelial apoptosis and inflammation (Vion et al., 2017). Also, KLF2/4 expression induce the production of NADPH, while reducing the expression of PFKFB3 (Doddaballapur et al., 2015).

However, due to stimuli like for example disturbed blood flow dynamics resulting in laminar shear stress, ECs can become activated (Gimbrone and Garcia-Cardena, 2016) (Figure 3). ECs at atheroprone regions in the vessels experiencing this disturbed shear stress demonstrate an activation of pro-inflammatory pathways and increased expression of glycolytic enzymes (Feng et al., 2017). Additionally, disturbed blood flow reduces KLF2/4 expression and thus NO and NADPH production, while PFKFB3 expression increases which is at least partly responsible for the increased glycolysis (Stroope et al., 2024). Furthermore, expressed pro-inflammatory cytokines in ECs increase the glucose uptake and glycolysis in these cells, via NF-κB activation (Cantelmo et al., 2016). Disturbed shear stress as well as pro-inflammatory cytokines also activate YAP, which not only stimulates EC activation and atherosclerosis development, but also EC glycolysis (Wang et al., 2016a). Additionally, disturbed shear stress activates AMPK and thereby induces HIF1α-mediated upregulation of glycolysis-related genes (Wu et al., 2017; Yang et al., 2018). Thereby, EC inflammation enhances glycolysis in these cells but also initiates a vicious cycle as this increased glycolysis again drives inflammation.

Interestingly, YAP also enhances EC glutaminolysis, suggesting a role for glutamine in atherogenesis (Bertero et al., 2016). Glutamine starvation of ECs reduces protein synthesis and thus results in ER stress and increased cellular inflammation (Tabas, 2010; Huang et al., 2017). However, the exact effects on atherosclerosis development remain to be investigated.

While the role of FAO in the inflammatory responses of ECs remains rather elusive, it could already be shown that FAO is important for maintaining the EC barrier function (Patella et al., 2015; Xiong et al., 2018). Furthermore, FAO inhibits EndMT which has a plaque destabilizing outcome (Chen et al., 2015; Xiong et al., 2018; Libby et al., 2019). Thereby endothelial FAO seems to reduce atherosclerosis development, although clear causal evaluations on this aspect are currently still missing.

### 6.3 Associated effects of atherosclerosis comorbidities on endothelial cell metabolism

Several comorbidities, such as T2DM and obesity, are hallmarked by endothelial dysfunction and a dysregulated EC metabolism, resulting in an accelerated development of atherosclerosis.

#### 6.3.1 T2DM

The high circulating glucose levels caused by T2DM increase endothelial ROS production, leading to DNA damage and subsequent activation of poly (ADP-ribose) polymerase 1 (PARP1) (Du et al., 2003; Forrester et al., 2018). This PARP1 activation causes an inhibition of the glycolytic enzyme GAPDH, resulting in an accumulation of intermediate products that are upstream of this enzyme (Du et al., 2003). This accumulation and further redistribution of glycolysis intermediates results in the generation of AGEs and PKC activation (Du et al., 2003; Shah and Brownlee, 2016). These AGEs induce EC dysfunction and activate pro-inflammatory signaling cascades together with ROS formation (Shah and Brownlee, 2016). Thereby, T2DM influences ECs in a manner that generates a more atherosclerosis-prone cellular phenotype.

#### 6.3.2 Obesity

One of the characteristics of obesity is the elevated circulating saturated fatty acids and triglyceride-rich lipoproteins, which are a major source of fatty acids (Goldberg and Bornfeldt, 2013; Nordestgaard, 2016). These fatty acids can induce EC dysfunction by impairing vasodilatation and increasing vascular permeability, oxidative stress, inflammatory signaling as well as EC apoptosis (Inoguchi et al., 2000; Wang et al., 2016b). This is further supported by the notion that overexpression of PPAR-γ coactivator 1-α, resulting in increased FAO and thus reduced intracellular lipid levels, reduces again the FA-induced EC dysfunction and apoptosis (Won et al., 2010). Intriguingly, it remains to be determined why ECs do not spontaneously increase FAO to compensate for the lipid overload, particularly considering their large spare respiratory capacity.

## 7 Metabolic alterations in vascular smooth muscle cells in the context of inflammation and atherosclerosis

VSMCs play a key role in the process of atherosclerosis (Grootaert and Bennett, 2021). Although VSMCs have previously mainly been appreciated for their role in fibrous cap formation and thus atheroprotective function, multiple VSMC phenotypes and detrimental VSMC functions have now been identified in the context of atherosclerosis (Grootaert and Bennett, 2021; Chen et al., 2023; Elmarasi et al., 2024). Contractile VSMCs populate the medial layer of the healthy vessel. In the pre-atherosclerotic stage, characterized by a diffuse intimal thickening, an increase in synthetic VSMCs can be detected. With the progression of atherosclerotic lesions, medial VSMCs can migrate from the media to the intima and generate extracellular matrix molecules (including interstitial collagen and elastin, as well as proteoglycans and glycosaminoglycans), forming a protective fibrous cap over the developing lesion (Bennett et al., 2016; Basatemur et al., 2019). On the other hand, synthetic VSMCs can differentiate into cells resembling macrophages (Basatemur et al., 2019; Elmarasi et al., 2024). Increased lipid buildup, inflammation and VSMC death, as well as VSMC differentiation into osteochondrogenic cells (contributing to plaque calcification) are observed in later stages of atherosclerosis progression (Basatemur et al., 2019; Elmarasi et al., 2024). Over the past few decades, a growing body of research has revealed a role of cellular metabolism – particularly glucose uptake, glycolysis, and amino acid metabolism – in the regulation of VSMC phenotype (Stroope et al., 2024).

### 7.1 VSMC metabolism in health and atherosclerosis

#### 7.1.1 Glycolysis

In healthy VSMCs, cytoplasmic or aerobic glycolysis is central for ATP production and triggers high rates of lactate production under fully oxygenated conditions, rather than relying on glucose oxidation in the mitochondria (Lynch and Paul, 1983; Butler and Siegman, 1985). Overall, 90% of the glucose taken up by VSMCs is converted to lactate (Michelakis and Weir, 2008).

**FIGURE 4:**
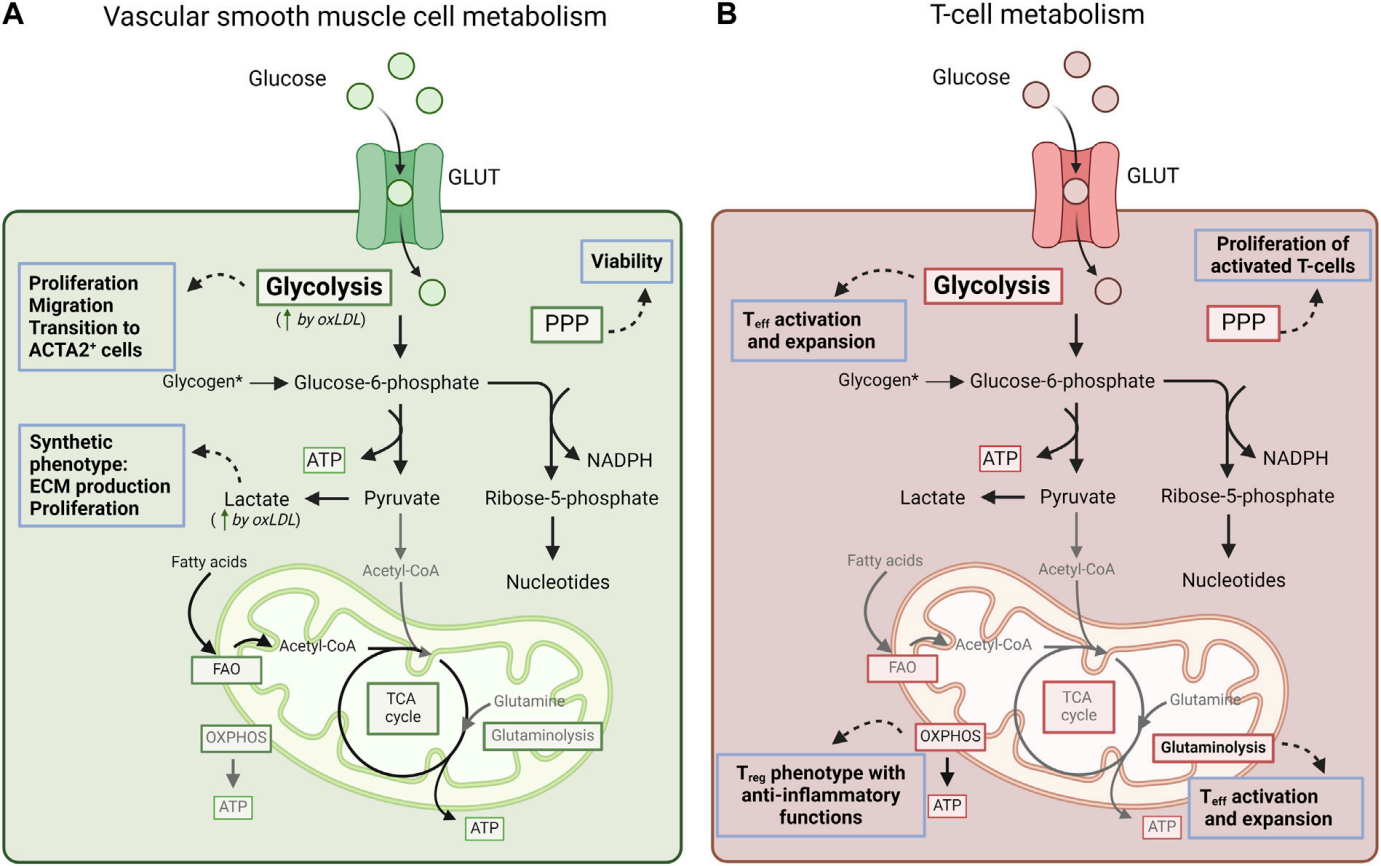
Summary of cellular metabolism in vascular smooth muscle cells and T-lymphocytes. **(A)** Overall, 90% of the glucose taken up by VSMCs is converted to lactate by aerobic glycolysis. Glycolysis supports VSMC proliferation and migration, and triggers the transition of VSMCs to ACTA2^+^ myofibroblast-like cells. Increased lactate levels support a more synthetic phenotype of VSMCs with increased extracellular matrix production and VSMC proliferation. OxLDL increases glycolysis rates and lactate production. Furthermore, PPP activation has been linked with increased VSMC viability. VSMCs can also use fatty acids for ATP production as shown in glucose-free medium. Overall, the impact of metabolic pathway alterations in VSMCs on atherosclerosis outcome remains to be further investigated. **(B)** Activation of effector T-cells (T_eff_) increases glycolysis and glutaminolysis, which both support lipid biosynthesis (and thus the possibility of cell expansion) via the production of citrate. Also, the PPP supports the proliferation of activated T-cells by providing precursors and NADPH for the biosynthesis of nucleotides, amino acids and fatty acids. Instead, regulatory T-cells (T_reg_) depend on OXPHOS-dependent metabolism to exert their anti-inflammatory function. ACTA2, Actin alpha 2, smooth muscle; ATP, adenosine triphosphate; NADPH, nicotinamide adenine dinucleotide phosphate; OXPHOS, oxidative phosphorylation; PPP, pentose-phosphate pathway; VSMC, vascular smooth muscle cells.

*In vitro*, platelet-derived growth factor (PDGF)-BB-induced proliferation of VSMCs markedly increased glucose flow and glycolysis (Werle et al., 2005). Blocking glycolysis with 2-deoxy-D-glucose or blocking phosphoinositide 3-kinase (PI3K) prevented both PDGF-stimulated VSMCs proliferation (Figure 4A) as well as the increase in glycolysis and mitochondrial reserve capacity (Perez et al., 2010). In line, deletion of PKM2 – which catalyzes the last step of glycolysis – in VSMCs attenuated injury-induced neointimal hyperplasia by hindering VSMC proliferation and migration, suppressing the synthetic phenotype and diminishing aerobic glycolysis by decreasing signaling through extracellular signal-regulated kinase (ERK), mTOR and STAT3 pathways (Jain et al., 2021). Furthermore, single-cell and bulk RNA-seq analyses of mouse brachiocephalic arteries and *in vitro* models showed that aerobic glycolysis is important for the transition of VSMCs to ACTA2^+^ myofibroblast-like cells, which play an important role in the formation of the protective fibrous cap in atherosclerotic lesions (Newman et al., 2021) (Figure 4A). Furthermore, increased lactate levels - as observed in anaerobic conditions as ischemia injury (Yang et al., 2017) as well as in the necrotic core of atherosclerotic lesions (Seeley et al., 2023) - supported a more synthetic phenotype of VSMCs, with a proteomics and pathway profile suggestive of increased extracellular matrix production and VSMC proliferation (Yang et al., 2017) (Figure 4A). Also network analyses based on gene expression studies revealed dysregulations in glycolysis metabolism during human aortic SMCs phenotype plasticity (Perry et al., 2023).

OxLDL as important contributor to atherosclerosis induces the generation of ROS and oxidative stress in VSMCs (Locher et al., 2002). High concentrations of OxLDL can also exacerbate apoptosis in VSMCs, which could support the destabilization of atherosclerotic plaques (Kataoka et al., 2001). In terms of cellular metabolism, oxLDL significantly enhanced the PKM2-dependent glycolytic rate and established glycolysis as the primary energy source in oxLDL-treated VSMCs (Zhao et al., 2020). This metabolic shift was evidenced by increased lactate and ATP production, while mitochondrial OXPHOS remained unchanged (Zhao et al., 2020).

#### 7.1.2 Pentose phosphate pathway

The PPP plays a critical role in maintaining the redox balance and supporting biosynthetic processes through the generation of NADPH, essential for glutathione reduction and counteracting oxidative stress. Accordingly, PPP activation with increased G6PD activity and NADPH production has been linked with increased VSMC viability (Dong et al., 2015) (Figure 4A).

#### 7.1.3 Fatty acid metabolism

Saturated free fatty acids, such as palmitic acid, have been shown to trigger pro-inflammatory responses in a multitude of cell types (Soppert et al., 2020; Noels et al., 2021), including VSMCs (Ma et al., 2011; Quan et al., 2014). Furthermore, administering the monounsaturated free fatty acid oleic acid to VSMCs *in vitro* or elevating its plasma levels upon feeding mice with a diet enriched in oleic acid stimulated VSMC foam cell formation through CD36 and the development of atherosclerotic lesions (Ma et al., 2011). Oleic acid could also trigger SMC proliferation via the free fatty acid receptor 1 (FFAR1) and signaling through PI3K/AKT and MEK/ERK (Matoba et al., 2018).

In relation to energy production, VSMCs can use fatty acid for ATP production, as shown by incubating VSMCs with octanoate in glucose-free medium, which increased oxygen consumption and could cover for the reduction in glucose-dependent ATP production (Barron et al., 1994). FAO was also reported to be increased upon PDGF-BB treatment of VSMCs with a concomitant downregulation of glycolysis (Salabei and Hill, 2013), the latter however in contrast to other studies (Werle et al., 2005).

#### 7.1.4 Amino acid metabolism

Arginine, homocysteine, glutamine, and tryptophan contribute to phenotypic changes of VSMCs.

##### 7.1.4.1 Arginine

Plaque stability and VSMC phenotypic regulation are critically dependent on arginine metabolism. NO can be produced by arginine metabolism (Zhao et al., 2015). While EC-derived NO stimulates vasodilation through cyclic GMP-mediated activation of soluble guanylate cyclase in VSMCs, NO produced by VSMCs functions as a free radical that inhibits proliferation and migration, and induces apoptosis (Ignarro et al., 2001; Tzeng et al., 2017). Furthermore, arginine is converted by ARG1 to ornithine, which is subsequently converted to the polyamines putrescine, spermidine and spermine. Inhibiting ARG1 impeded VSMC proliferation (Ignarro et al., 2001). In line, ARG1 stimulated VSMC proliferation, reduced the expression of inflammatory cytokines in macrophages and improved the stability of atherosclerotic plaque *in vivo* (Wang et al., 2014). Also, treatment with spermidine mitigated necrotic core expansion by stimulating VSMC autophagy and preventing lipid accumulation by triggering cholesterol efflux from VSMCs (Michiels et al., 2016).

##### 7.1.4.2 Homocysteine

Homocysteine triggers VSMC proliferation (Tsai et al., 1994) and VSMC phenotypic switching to a synthetic phenotype via endothelin-1 signaling (Chen et al., 2020). In line, treating patients with folate to reduce homocysteine levels dramatically lowered the rate of restenosis following coronary angioplasty (Schnyder et al., 2001), which is driven by injury-induced VSMC proliferation. Also, homocysteine triggered ROS production in VSMCs (Chang et al., 2004).

##### 7.1.4.3 Glutamine

Blocking glutamine transport and thereby glutamine metabolism inhibited serum- and PDGF-BB-induced VSMC proliferation and migration *in vitro*, as well as injury-induced neointima formation in mice (Park et al., 2021).

##### 7.1.4.4 Tryptophan

In VSMCs, the specific deletion of the tryptophan-catabolizing enzyme indoleamine 2,3-dioxygenase 1 (IDO1) increased the expression of runt-related transcription factor 2 (RUNX2) and exacerbated vascular calcification, which could be reversed by administering the IDO1 product kynurenine (Ouyang et al., 2022). Also, by activating the aryl hydrocarbon receptor, kynurenine limited the transition of VSMCs to chondrocytes and maintained the integrity of the fibrous cap in atherosclerotic lesions (Kim et al., 2020). The tryptophan metabolite 3-hydroxyanthranilic acid can activate NF-κB and increase MMP2 expression in VSMCs (Wang et al., 2017).

### 7.2 Associated effects of atherosclerosis comorbidities on VSMC metabolism

Comorbidities as hypertension and T2DM are also associated with an altered VSMC phenotype and/or function.

#### 7.2.1 Hypertension

Abnormal proliferation, migration and apoptosis of VSMCs can lead to vascular remodeling and increased peripheral resistance (Guarner-Lans et al., 2020). VSMCs of spontaneously hypertensive rats (SHR) displayed hypertrophy and produced more extracellular matrix while having reduced cell death and apoptosis rates compared to VSMCs from normotensive rats (Arribas et al., 2010). Furthermore, with endothelial NO production triggering vasodilation in VSMCs, endothelial dysfunction as observed in hypertension contributes to vasoconstriction via decreased NO production (Wu Y. et al., 2021). *In vitro*, VSMCs from SHR rats also showed increased ROS production and NADPH activity along with hyperproliferation compared with normotensive rats, which could be counteracted by increasing intracellular NO levels (Sarkar et al., 2017). In terms of metabolic pathways, reduced glucose uptake and metabolism in VSMCs due to reduced vascular expression of GLUT4 may contribute to the contractile dysfunction observed in hypertension (Atkins et al., 2001).

#### 7.2.2 T2DM

In conditions of high glucose, the pro-inflammatory cytokine IL-1β induced an increase in glucose uptake as well as glucose metabolism over the PPP via upregulated G6PD expression. This triggered enhanced NADPH oxidase activity, resulting in increased ROS production and pro-inflammatory signaling (Peiró et al., 2016). Along with ROS production and inflammatory signaling, VSMCs showed a marked increase in proliferation, migration and monocyte adhesion when exposed to high glucose levels, with a role for RAGE revealed herein (Su et al., 2019). In a mouse model of metabolic syndrome with *Ldlr* ^*−/−*^ mice on high-fat and high-sucrose diet, increased glycolysis through transgenic overexpression of GLUT1 in VSMCs – but not in myeloid cells - accelerated the development of atherosclerotic lesions and promoted monocytosis and the recruitment of pro-inflammatory Ly6C^high^ monocytes to lesions (Wall et al., 2018). Of note, this effect of VSMC-specific overexpression on atherosclerosis was not observed in mice without metabolic syndrome (Wall et al., 2018). *In vitro*, GLUT1 overexpression in VSMCs enhanced the TNFα-induced production of the pro-inflammatory cytokines TNFα and CCL2 (Wall et al., 2018).

In addition to a direct impact of high glucose levels, long-term hyperglycemia conditions can also lead to the accumulation of AGEs, which can stimulate the migration and proliferation of VSMCs as well as the production of extracellular matrix (Hwang et al., 2018). During VSMC calcification, AGEs reduced glycolysis and lactate production, the PPP as well as mitochondrial respiratory capacity, resulting in reduced glucose consumption. In parallel, AGEs increased vascular calcification via HIF-1α and pyruvate dehydrogenase kinase 4 (Zhu et al., 2018).

## 8 Metabolic alterations in lymphocytes in the context of inflammation and atherosclerosis

Also, lymphocytes impact on atherosclerosis. T helper 1 cells are pro-atherogenic, in contrast to regulatory T-cells (Tregs) exerting an atheroprotective function (Saigusa et al., 2020). B-cells can be either pro- or anti-atherosclerotic depending on the subset they belong to and their effector response involving antibody production (atheroprotective IgM vs. pro-atherogenic IgG and IgE) and cytokine production (Sage et al., 2019).

Cell metabolism of T- and B-lymphocytes has been studied intensively in the context of inflammation and auto-immune diseases (van der Windt and Pearce, 2012; Rhoads et al., 2017). Auto-immune responses also underlie lymphocyte responses in the context of atherosclerosis (Wolf et al., 2020; Wang et al., 2023) (Figure 4B). Lymphocyte activation triggers a shift to anabolic metabolism with increased glycolysis and glutaminolysis dependent on mTOR signaling, as shown for activated B-cells and effector T-cells (van der Windt and Pearce, 2012; Rhoads et al., 2017). Both metabolic pathways support lipid biosynthesis via the production of citrate, which in the cytosol can be converted to acetyl-coA as basis for lipid synthesis (van der Windt and Pearce, 2012). Also, the PPP supports the proliferation of activated T-cells by providing precursors for nucleotides and amino acids as well as generating NADPH to support lipid and nucleotide biosynthesis (van der Windt and Pearce, 2012). Memory lymphocytes convert back to catabolic cell metabolism with increased fatty acid and pyruvate oxidation via OXPHOS, although with enhanced metabolic flexibility to ensure fast secondary responses (Rhoads et al., 2017). Furthermore, Tregs depend on OXPHOS-dependent metabolism to exert their anti-inflammatory function (Rhoads et al., 2017). In line with these findings, nutrient alterations impact on lymphocyte differentiation and responses. For example, glucose restriction reduces effector T-cell responses and supports a differentiation towards Tregs (Kedia-Mehta and Finlay, 2019). Also, reduced glutamine availability reduces mTOR signaling and thereby shifts CD4^+^ T-cells towards a Treg phenotype (Klysz et al., 2015). For a more in-depth discussion of cell metabolism in T-lymphocytes in the context of atherosclerosis, we refer to another review (Stroope et al., 2024).

## 9 Conclusions

In summary, in the context of atherosclerosis, hypoxia and inflammatory triggers generally stimulate glycolysis and the PPP that govern the pro-inflammatory macrophage phenotype. Fatty acid synthesis and many amino acids generally also stimulate a pro-inflammatory phenotype. Macrophages stimulated with IL4/IL13 – acquiring an anti-inflammatory phenotype - downregulate glycolysis and the PPP, while upregulating OXPHOS and FAO. Immature neutrophils mostly rely on OXPHOS and FAO, while mature neutrophils and those forming NETs mainly depend on glycolysis and the PPP to generate NADPH and exert their function. ECs mainly depend on glycolysis and the PPP, both supporting EC proliferation and sprouting. However, ECs can also use FAO, which supports cell proliferation and counteracts EndMT. Endothelial activation and disturbed flow – as in the context of atherosclerosis – are associated with increased glycolysis, which on its turn further contributes to endothelial inflammation. In VSMCs, increased glycolysis supports cell proliferation as well as the transition to a synthetic phenotype, while the PPP has been linked with increased VSMC viability. Finally, B- and T-lymphocytes upregulate glycolysis and glutaminolysis upon activation to support cell proliferation and cellular effector functions, additionally supported by the PPP. Instead, Tregs depend on OXPHOS-dependent metabolism to exert their anti-inflammatory function.

Thus, in most cell types associated with atherosclerosis including macrophages, neutrophils, ECs and T-lymphocytes, pro-inflammatory conditions are associated with increased glycolysis and – for macrophages and neutrophils – an increase in the PPP. Instead, in VSMCs, additional studies directly addressing cellular metabolism alterations and the direct impact on atherosclerosis are required.

Cardiovascular risk factors as obesity, T2DM and hypertension may impact on cellular metabolism and thereby on inflammatory processes and cardiovascular risk. Obesity, T2DM and hypertension trigger a pro-inflammatory phenotype in macrophages as well as endothelial cell dysfunction, and also ROS generation and NET formation are dysregulated in obesity and T2DM. However, the relation with cellular metabolism changes in cardiovascular comorbidities and risk factors remains to be further elucidated.

Of note, most observations in this review have been derived from *in vitro* experiments. Instead, relatively few studies addressed the *in vivo* impact of altering cell metabolism, and it needs to be taken into account that cell-specific interventions *in vivo* did not always show similar changes in cell state or function. This could be the result of a change in cell metabolism of one cell type that will alter substrate availability for other cells, and hence affect the overall microenvironment. Also, metabolites of cellular metabolism contribute to cellular crosstalk, which impacts disease development and progression. Such cellular crosstalk further complicates unraveling the impact of cell metabolism changes on inflammation and atherosclerosis. Additional complexity is added by the observation that specific metabolic pathways can have both pro- and anti-inflammatory effects even in the same cell type (for example glycolysis driving the macrophage M1 phenotype but also being involved in efferocytosis) or may have differential effects in terms of inflammatory outcome in different cell types. Thus, although interventions in these pathways may seem an attractive therapeutic strategy, the risk of adverse effects of systemic and cell type-specific effects cannot be ignored. Therefore, the impact of cell type-specific changes in metabolism needs to be explored more widely *in vivo*, with special attention to systemic effects. By the time that cell type-specific treatments are achievable by delivering therapeutics in a directed way through nanoparticle-based strategies, this information will be crucial to decide on the most effective metabolic intervention to ameliorate atherosclerosis and its interplay with metabolic co-morbidities.

## References

[B1] Alabduladhem T. O. Bordoni B. (2024). “Physiology, krebs cycle,” in StatPearls (Treasure Island (FL): StatPearls Publishing). 32310492

[B2] Alba-Loureiro T. C. Hirabara S. M. Mendonça J. R. Curi R. Pithon-Curi T. C. (2006). Diabetes causes marked changes in function and metabolism of rat neutrophils. J. Endocrinol. 188 (2), 295–303. 10.1677/joe.1.06438 16461555

[B3] Arnold P. K. Finley L. W. S. (2023). Regulation and function of the mammalian tricarboxylic acid cycle. J. Biol. Chem. 299 (2), 102838. 10.1016/j.jbc.2022.102838 36581208 PMC9871338

[B4] Arribas S. M. Hermida C. González M. C. Wang Y. Hinek A. (2010). Enhanced survival of vascular smooth muscle cells accounts for heightened elastin deposition in arteries of neonatal spontaneously hypertensive rats. Exp. Physiol. 95 (4), 550–560. 10.1113/expphysiol.2009.050971 20008031

[B5] Arts R. J. Novakovic B. Ter Horst R. Carvalho A. Bekkering S. Lachmandas E. (2016). Glutaminolysis and fumarate accumulation integrate immunometabolic and epigenetic programs in trained immunity. Cell Metab. 24 (6), 807–819. 10.1016/j.cmet.2016.10.008 27866838 PMC5742541

[B6] Atkins G. B. Jain M. K. (2007). Role of Krüppel-like transcription factors in endothelial biology. Circ. Res. 100 (12), 1686–1695. 10.1161/01.RES.0000267856.00713.0a 17585076

[B7] Atkins K. B. Johns D. Watts S. Clinton Webb R. Brosius F. C. (2001). Decreased vascular glucose transporter expression and glucose uptake in DOCA-salt hypertension. J. Hypertens. 19 (9), 1581–1587. 10.1097/00004872-200109000-00009 11564977

[B8] Awasthi D. Nagarkoti S. Sadaf S. Aggarwal H. Gupta S. K. Chandra T. (2023). Modulations in human neutrophil metabolome and S-glutathionylation of glycolytic pathway enzymes during the course of extracellular trap formation. Biochim. Biophys. Acta Mol. Basis Dis. 1869 (1), 166581. 10.1016/j.bbadis.2022.166581 36265832

[B9] Awasthi D. Nagarkoti S. Sadaf S. Chandra T. Kumar S. Dikshit M. (2019). Glycolysis dependent lactate formation in neutrophils: a metabolic link between NOX-dependent and independent NETosis. Biochim. Biophys. Acta Mol. Basis Dis. 1865 (12), 165542. 10.1016/j.bbadis.2019.165542 31473341

[B10] Azevedo E. P. Rochael N. C. Guimarães-Costa A. B. de Souza-Vieira T. S. Ganilho J. Saraiva E. M. (2015). A metabolic shift toward pentose phosphate pathway is necessary for amyloid fibril- and phorbol 12-myristate 13-Acetate-induced neutrophil extracellular trap (NET) formation. J. Biol. Chem. 290 (36), 22174–22183. 10.1074/jbc.M115.640094 26198639 PMC4571968

[B11] Baardman J. Verberk S. G. S. van der Velden S. Gijbels M. J. J. van Roomen C. Sluimer J. C. (2020). Macrophage ATP citrate lyase deficiency stabilizes atherosclerotic plaques. Nat. Commun. 11 (1), 6296. 10.1038/s41467-020-20141-z 33293558 PMC7722882

[B12] Baaten C. Vondenhoff S. Noels H. (2023). Endothelial cell dysfunction and increased cardiovascular risk in patients with chronic kidney disease. Circ. Res. 132 (8), 970–992. 10.1161/CIRCRESAHA.123.321752 37053275 PMC10097498

[B13] Badimon L. Vilahur G. (2015). Neutrophil extracellular traps: a new source of tissue factor in atherothrombosis. Eur. Heart J. 36 (22), 1364–1366. 10.1093/eurheartj/ehv105 25845929

[B14] Baillet A. Hograindleur M. A. El Benna J. Grichine A. Berthier S. Morel F. (2017). Unexpected function of the phagocyte NADPH oxidase in supporting hyperglycolysis in stimulated neutrophils: key role of 6-phosphofructo-2-kinase. Faseb J. 31 (2), 663–673. 10.1096/fj.201600720R 27799347

[B15] Baldus S. Heeschen C. Meinertz T. Zeiher A. M. Eiserich J. P. Münzel T. (2003). Myeloperoxidase serum levels predict risk in patients with acute coronary syndromes. Circulation 108 (12), 1440–1445. 10.1161/01.CIR.0000090690.67322.51 12952835

[B16] Barhoumi T. Todryk S. (2023). Role of monocytes/macrophages in renin-angiotensin system-induced hypertension and end organ damage. Front. Physiol. 14, 1199934. 10.3389/fphys.2023.1199934 37854465 PMC10579565

[B17] Barron J. T. Kopp S. J. Tow J. Parrillo J. E. (1994). Fatty acid, tricarboxylic acid cycle metabolites, and energy metabolism in vascular smooth muscle. Am. J. Physiol. 267 (2 Pt 2), H764–H769. 10.1152/ajpheart.1994.267.2.H764 8067432

[B18] Barth E. Stämmler G. Speiser B. Schaper J. (1992). Ultrastructural quantitation of mitochondria and myofilaments in cardiac muscle from 10 different animal species including man. J. Mol. Cell Cardiol. 24 (7), 669–681. 10.1016/0022-2828(92)93381-s 1404407

[B19] Basatemur G. L. Jørgensen H. F. Clarke M. C. H. Bennett M. R. Mallat Z. (2019). Vascular smooth muscle cells in atherosclerosis. Nat. Rev. Cardiol. 16 (12), 727–744. 10.1038/s41569-019-0227-9 31243391

[B20] Batista-Gonzalez A. Vidal R. Criollo A. Carreno L. J. (2019). New insights on the role of lipid metabolism in the metabolic reprogramming of macrophages. Front. Immunol. 10, 2993. 10.3389/fimmu.2019.02993 31998297 PMC6966486

[B21] Behnen M. Möller S. Brozek A. Klinger M. Laskay T. (2017). Extracellular acidification inhibits the ROS-dependent formation of neutrophil extracellular traps. Front. Immunol. 8, 184. 10.3389/fimmu.2017.00184 28293240 PMC5329032

[B22] Bekkering S. Quintin J. Joosten L. A. van der Meer J. W. Netea M. G. Riksen N. P. (2014). Oxidized low-density lipoprotein induces long-term proinflammatory cytokine production and foam cell formation via epigenetic reprogramming of monocytes. Arteriosclerosis, thrombosis, Vasc. Biol. 34 (8), 1731–1738. 10.1161/ATVBAHA.114.303887 24903093

[B23] Bennett M. R. Sinha S. Owens G. K. (2016). Vascular smooth muscle cells in atherosclerosis. Circ. Res. 118 (4), 692–702. 10.1161/CIRCRESAHA.115.306361 26892967 PMC4762053

[B24] Bertero T. Oldham W. M. Cottrill K. A. Pisano S. Vanderpool R. R. Yu Q. (2016). Vascular stiffness mechanoactivates YAP/TAZ-dependent glutaminolysis to drive pulmonary hypertension. J. Clin. Invest. 126 (9), 3313–3335. 10.1172/JCI86387 27548520 PMC5004943

[B25] Borissoff J. I. Joosen I. A. Versteylen M. O. Brill A. Fuchs T. A. Savchenko A. S. (2013). Elevated levels of circulating DNA and chromatin are independently associated with severe coronary atherosclerosis and a prothrombotic state. Arteriosclerosis, thrombosis, Vasc. Biol. 33 (8), 2032–2040. 10.1161/ATVBAHA.113.301627 PMC3806482 23818485

[B26] Brinkmann V. Reichard U. Goosmann C. Fauler B. Uhlemann Y. Weiss D. S. (2004). Neutrophil extracellular traps kill bacteria. Science 303 (5663), 1532–1535. 10.1126/science.1092385 15001782

[B27] Britt E. C. Lika J. Giese M. A. Schoen T. J. Seim G. L. Huang Z. (2022). Switching to the cyclic pentose phosphate pathway powers the oxidative burst in activated neutrophils. Nat. Metab. 4 (3), 389–403. 10.1038/s42255-022-00550-8 35347316 PMC8964420

[B28] Butler T. M. Siegman M. J. (1985). High-energy phosphate metabolism in vascular smooth muscle. Annu. Rev. Physiol. 47, 629–643. 10.1146/annurev.ph.47.030185.003213 3158271

[B29] Cahilly C. Ballantyne C. M. Lim D. S. Gotto A. Marian A. J. (2000). A variant of p22(phox), involved in generation of reactive oxygen species in the vessel wall, is associated with progression of coronary atherosclerosis. Circ. Res. 86 (4), 391–395. 10.1161/01.res.86.4.391 10700443

[B30] Cantelmo A. R. Conradi L. C. Brajic A. Goveia J. Kalucka J. Pircher A. (2016). Inhibition of the glycolytic activator PFKFB3 in endothelium induces tumor vessel normalization, impairs metastasis, and improves chemotherapy. Cancer Cell 30 (6), 968–985. 10.1016/j.ccell.2016.10.006 27866851 PMC5675554

[B31] Chacko B. K. Kramer P. A. Ravi S. Johnson M. S. Hardy R. W. Ballinger S. W. (2013). Methods for defining distinct bioenergetic profiles in platelets, lymphocytes, monocytes, and neutrophils, and the oxidative burst from human blood. Lab. Invest. 93 (6), 690–700. 10.1038/labinvest.2013.53 23528848 PMC3674307

[B32] Chandel N. S. (2021a). Metabolism of proliferating cells. Cold Spring Harb. Perspect. Biol. 13 (5), a040618. 10.1101/cshperspect.a040618 34598925 PMC8485748

[B33] Chandel N. S. (2021b). Metabolism of proliferating cells. Cold Spring Harb. Perspect. Biol. 13 (4), a040618. 10.1101/cshperspect.a040618 34598925 PMC8485748

[B34] Chang L. Xu J. X. Zhao J. Pang Y. Z. Tang C. S. Qi Y. F. (2004). Taurine antagonized oxidative stress injury induced by homocysteine in rat vascular smooth muscle cells. Acta Pharmacol. Sin. 25 (3), 341–346. 10.1172/jci82719 15000888

[B35] Chen P. Y. Qin L. Baeyens N. Li G. Afolabi T. Budatha M. (2015). Endothelial-to-mesenchymal transition drives atherosclerosis progression. J. Clin. Invest. 125 (12), 4514–4528. 10.1172/JCI82719 26517696 PMC4665771

[B36] Chen R. McVey D. G. Shen D. Huang X. Ye S. (2023). Phenotypic switching of vascular smooth muscle cells in atherosclerosis. J. Am. Heart Assoc. 12 (20), e031121. 10.1161/JAHA.123.031121 37815057 PMC10757534

[B37] Chen Y. Su X. Qin Q. Yu Y. Jia M. Zhang H. (2020). New insights into phenotypic switching of VSMCs induced by hyperhomocysteinemia: role of endothelin-1 signaling. Biomed. Pharmacother. 123, 109758. 10.1016/j.biopha.2019.109758 31864211

[B38] Chen Y. Yang M. Huang W. Chen W. Zhao Y. Schulte M. L. (2019). Mitochondrial metabolic reprogramming by CD36 signaling drives macrophage inflammatory responses. Circ. Res. 125 (12), 1087–1102. 10.1161/CIRCRESAHA.119.315833 31625810 PMC6921463

[B39] Cheng S. C. Quintin J. Cramer R. A. Shepardson K. M. Saeed S. Kumar V. (2014). mTOR- and HIF-1α-mediated aerobic glycolysis as metabolic basis for trained immunity. Science 345 (6204), 1250684. 10.1126/science.1250684 25258083 PMC4226238

[B40] Chinetti-Gbaguidi G. Baron M. Bouhlel M. A. Vanhoutte J. Copin C. Sebti Y. (2011). Human atherosclerotic plaque alternative macrophages display low cholesterol handling but high phagocytosis because of distinct activities of the PPARγ and LXRα pathways. Circ. Res. 108 (8), 985–995. 10.1161/CIRCRESAHA.110.233775 21350215 PMC3319502

[B41] Chistiakov D. A. Bobryshev Y. V. Orekhov A. N. (2015). Neutrophil's weapons in atherosclerosis. Exp. Mol. Pathol. 99 (3), 663–671. 10.1016/j.yexmp.2015.11.011 26551083

[B291] Cichon I. Ortmann W. Kolaczkowska E. (2021). Metabolic pathways involved in formation of spontaneous and lipopolysaccharide-induced neutrophil extracellular traps (NETs) differ in obesity and systemic inflammation. Int. J. Mol. Sci. 22 (14), 13730. 10.3390/ijms22147718IF PMC8303382 34299338

[B42] Dabravolski S. A. Sukhorukov V. N. Kalmykov V. A. Grechko A. V. Shakhpazyan N. K. Orekhov A. N. (2022). The role of KLF2 in the regulation of atherosclerosis development and potential use of KLF2-targeted therapy. Biomedicines 10 (2), 254. 10.3390/biomedicines10020254 35203463 PMC8869605

[B43] De Bock K. Georgiadou M. Schoors S. Kuchnio A. Wong B. W. Cantelmo A. R. (2013). Role of PFKFB3-driven glycolysis in vessel sprouting. Cell 154 (3), 651–663. 10.1016/j.cell.2013.06.037 23911327

[B44] Delamaire M. Maugendre D. Moreno M. Le Goff M. C. Allannic H. Genetet B. (1997). Impaired leucocyte functions in diabetic patients. Diabet. Med. 14 (1), 29–34. 10.1002/(SICI)1096-9136(199701)14:1<29::AID-DIA300>3.0.CO;2-V 9017350

[B45] de Winther M. P. J. Bäck M. Evans P. Gomez D. Goncalves I. Jørgensen H. F. (2023). Translational opportunities of single-cell biology in atherosclerosis. Eur. Heart J. 44 (14), 1216–1230. 10.1093/eurheartj/ehac686 36478058 PMC10120164

[B46] Dib L. Koneva L. A. Edsfeldt A. Zurke Y. X. Sun J. Nitulescu M. (2023). Lipid-associated macrophages transition to an inflammatory state in human atherosclerosis increasing the risk of cerebrovascular complications. Nat. Cardiovasc Res. 2 (7), 656–672. 10.1038/s44161-023-00295-x 38362263 PMC7615632

[B47] Di Gioia M. Spreafico R. Springstead J. R. Mendelson M. M. Joehanes R. Levy D. (2020). Endogenous oxidized phospholipids reprogram cellular metabolism and boost hyperinflammation. Nat. Immunol. 21 (1), 42–53. 10.1038/s41590-019-0539-2 31768073 PMC6923570

[B48] Dinauer M. C. Pierce E. A. Bruns G. A. Curnutte J. T. Orkin S. H. (1990). Human neutrophil cytochrome b light chain (p22-phox). Gene structure, chromosomal location, and mutations in cytochrome-negative autosomal recessive chronic granulomatous disease. J. Clin. Invest. 86 (5), 1729–1737. 10.1172/JCI114898 2243141 PMC296926

[B49] Dionisio F. Tomas L. Schulz C. (2023). Glycolytic side pathways regulating macrophage inflammatory phenotypes and functions. Am. J. Physiol. Cell Physiol. 324 (2), C558–C564. 10.1152/ajpcell.00276.2022 36645667

[B50] Doddaballapur A. Michalik K. M. Manavski Y. Lucas T. Houtkooper R. H. You X. (2015). Laminar shear stress inhibits endothelial cell metabolism via KLF2-mediated repression of PFKFB3. Arteriosclerosis, thrombosis, Vasc. Biol. 35 (1), 137–145. 10.1161/ATVBAHA.114.304277 25359860

[B51] Dong L. H. Li L. Song Y. Duan Z. L. Sun S. G. Lin Y. L. (2015). TRAF6-Mediated SM22α K21 ubiquitination promotes G6PD activation and NADPH production, contributing to GSH homeostasis and VSMC survival *in vitro* and *in vivo* . Circ. Res. 117 (8), 684–694. 10.1161/CIRCRESAHA.115.306233 26291555

[B52] Dos Santos L. M. da Silva T. M. Azambuja J. H. Ramos P. T. Oliveira P. S. da Silveira E. F. (2017). Methionine and methionine sulfoxide treatment induces M1/classical macrophage polarization and modulates oxidative stress and purinergic signaling parameters. Mol. Cell Biochem. 424 (1-2), 69–78. 10.1007/s11010-016-2843-6 27752805

[B53] Drechsler M. Megens R. T. van Zandvoort M. Weber C. Soehnlein O. (2010). Hyperlipidemia-triggered neutrophilia promotes early atherosclerosis. Circulation 122 (18), 1837–1845. 10.1161/CIRCULATIONAHA.110.961714 20956207

[B54] Du X. Matsumura T. Edelstein D. Rossetti L. Zsengellér Z. Szabó C. (2003). Inhibition of GAPDH activity by poly(ADP-ribose) polymerase activates three major pathways of hyperglycemic damage in endothelial cells. J. Clin. Invest. 112 (7), 1049–1057. 10.1172/JCI18127 14523042 PMC198524

[B55] Eguchi K. Manabe I. (2013). Macrophages and islet inflammation in type 2 diabetes. Diabetes Obes. Metab. 15 (Suppl. 3), 152–158. 10.1111/dom.12168 24003932

[B56] Elmarasi M. Elmakaty I. Elsayed B. Elsayed A. Zein J. A. Boudaka A. (2024). Phenotypic switching of vascular smooth muscle cells in atherosclerosis, hypertension, and aortic dissection. J. Cell Physiol. 239 (4), e31200. 10.1002/jcp.31200 38291732

[B57] Feng S. Bowden N. Fragiadaki M. Souilhol C. Hsiao S. Mahmoud M. (2017). Mechanical activation of hypoxia-inducible factor 1α drives endothelial dysfunction at atheroprone sites. Arteriosclerosis, thrombosis, Vasc. Biol. 37 (11), 2087–2101. 10.1161/ATVBAHA.117.309249 PMC5659306 28882872

[B58] Ferrante A. Nandoskar M. Walz A. Goh D. H. Kowanko I. C. (1988). Effects of tumour necrosis factor alpha and interleukin-1 alpha and beta on human neutrophil migration, respiratory burst and degranulation. Int. Arch. Allergy Appl. Immunol. 86 (1), 82–91. 10.1159/000234610 3286522

[B59] Folco E. J. Mawson T. L. Vromman A. Bernardes-Souza B. Franck G. Persson O. (2018). Neutrophil extracellular traps induce endothelial cell activation and tissue factor production through interleukin-1α and cathepsin G. Arteriosclerosis, thrombosis, Vasc. Biol. 38 (8), 1901–1912. 10.1161/ATVBAHA.118.311150 PMC6202190 29976772

[B60] Folco E. J. Sheikine Y. Rocha V. Z. Christen T. Shvartz E. Sukhova G. K. (2011). Hypoxia but not inflammation augments glucose uptake in human macrophages: implications for imaging atherosclerosis with 18fluorine-labeled 2-deoxy-D-glucose positron emission tomography. J. Am. Coll. Cardiol. 58 (6), 603–614. 10.1016/j.jacc.2011.03.044 21798423

[B61] Folco E. J. Sukhova G. K. Quillard T. Libby P. (2014). Moderate hypoxia potentiates interleukin-1β production in activated human macrophages. Circ. Res. 115 (10), 875–883. 10.1161/CIRCRESAHA.115.304437 25185259 PMC4209192

[B62] Forrester S. J. Kikuchi D. S. Hernandes M. S. Xu Q. Griendling K. K. (2018). Reactive oxygen species in metabolic and inflammatory signaling. Circ. Res. 122 (6), 877–902. 10.1161/CIRCRESAHA.117.311401 29700084 PMC5926825

[B63] Franceschi T. S. Soares M. S. P. Pedra N. S. Bona N. P. Spohr L. Teixeira F. C. (2020). Characterization of macrophage phenotype, redox, and purinergic response upon chronic treatment with methionine and methionine sulfoxide in mice. Amino Acids 52 (4), 629–638. 10.1007/s00726-020-02841-4 32246211

[B64] Franck G. Mawson T. Sausen G. Salinas M. Masson G. S. Cole A. (2017). Flow perturbation mediates neutrophil recruitment and potentiates endothelial injury via TLR2 in mice: implications for superficial erosion. Circ. Res. 121 (1), 31–42. 10.1161/CIRCRESAHA.117.310694 28428204 PMC5488735

[B65] Freigang S. Ampenberger F. Weiss A. Kanneganti T. D. Iwakura Y. Hersberger M. (2013). Fatty acid-induced mitochondrial uncoupling elicits inflammasome-independent IL-1α and sterile vascular inflammation in atherosclerosis. Nat. Immunol. 14 (10), 1045–1053. 10.1038/ni.2704 23995233

[B66] Fuchs T. A. Brill A. Duerschmied D. Schatzberg D. Monestier M. Myers D. D. Jr. (2010). Extracellular DNA traps promote thrombosis. Proc. Natl. Acad. Sci. U. S. A. 107 (36), 15880–15885. 10.1073/pnas.1005743107 20798043 PMC2936604

[B67] Fujisaka S. Usui I. Ikutani M. Aminuddin A. Takikawa A. Tsuneyama K. (2013). Adipose tissue hypoxia induces inflammatory M1 polarity of macrophages in an HIF-1α-dependent and HIF-1α-independent manner in obese mice. Diabetologia 56 (6), 1403–1412. 10.1007/s00125-013-2885-1 23494472

[B68] Furukawa S. Saito H. Inoue T. Matsuda T. Fukatsu K. Han I. (2000). Supplemental glutamine augments phagocytosis and reactive oxygen intermediate production by neutrophils and monocytes from postoperative patients *in vitro* . Nutrition 16 (5), 323–329. 10.1016/s0899-9007(00)00228-8 10793298

[B69] Gimbrone M. A. Jr. Garcia-Cardena G. (2016). Endothelial cell dysfunction and the pathobiology of atherosclerosis. Circ. Res. 118 (4), 620–636. 10.1161/CIRCRESAHA.115.306301 26892962 PMC4762052

[B70] Goldberg I. J. Bornfeldt K. E. (2013). Lipids and the endothelium: bidirectional interactions. Curr. Atheroscler. Rep. 15 (11), 365. 10.1007/s11883-013-0365-1 24037142 PMC3825167

[B71] Gómez-Escudero J. Clemente C. García-Weber D. Acín-Pérez R. Millán J. Enríquez J. A. (2019). PKM2 regulates endothelial cell junction dynamics and angiogenesis via ATP production. Sci. Rep. 9 (1), 15022. 10.1038/s41598-019-50866-x 31636306 PMC6803685

[B72] Grässle S. Huck V. Pappelbaum K. I. Gorzelanny C. Aponte-Santamaría C. Baldauf C. (2014). von Willebrand factor directly interacts with DNA from neutrophil extracellular traps. Arteriosclerosis, thrombosis, Vasc. Biol. 34 (7), 1382–1389. 10.1161/ATVBAHA.113.303016 24790143

[B73] Groh L. A. Ferreira A. V. Helder L. van der Heijden C. Novakovic B. van de Westerlo E. (2021). oxLDL-induced trained immunity is dependent on mitochondrial metabolic reprogramming. Immunometabolism 3 (3), e210025. 10.20900/immunometab20210025 34267957 PMC7611242

[B74] Grootaert M. O. J. Bennett M. R. (2021). Vascular smooth muscle cells in atherosclerosis: time for a re-assessment. Cardiovasc Res. 117 (11), 2326–2339. 10.1093/cvr/cvab046 33576407 PMC8479803

[B75] Guarner-Lans V. Ramírez-Higuera A. Rubio-Ruiz M. E. Castrejón-Téllez V. Soto M. E. Pérez-Torres I. (2020). Early programming of adult systemic essential hypertension. Int. J. Mol. Sci. 21 (4), 1203. 10.3390/ijms21041203 32054074 PMC7072742

[B76] Guixé-Muntet S. de Mesquita F. C. Vila S. Hernández-Gea V. Peralta C. García-Pagán J. C. (2017). Cross-talk between autophagy and KLF2 determines endothelial cell phenotype and microvascular function in acute liver injury. J. Hepatol. 66 (1), 86–94. 10.1016/j.jhep.2016.07.051 27545498

[B77] Hajra L. Evans A. I. Chen M. Hyduk S. J. Collins T. Cybulsky M. I. (2000). The NF-kappa B signal transduction pathway in aortic endothelial cells is primed for activation in regions predisposed to atherosclerotic lesion formation. Proc. Natl. Acad. Sci. U. S. A. 97 (16), 9052–9057. 10.1073/pnas.97.16.9052 10922059 PMC16820

[B78] Harlacher E. Wollenhaupt J. Baaten C. Noels H. (2022). Impact of uremic toxins on endothelial dysfunction in chronic kidney disease: a systematic review. Int. J. Mol. Sci. 23 (1), 531. 10.3390/ijms23010531 35008960 PMC8745705

[B79] Harwani S. C. (2018). Macrophages under pressure: the role of macrophage polarization in hypertension. Transl. Res. 191, 45–63. 10.1016/j.trsl.2017.10.011 29172035 PMC5733698

[B80] Haschemi A. Kosma P. Gille L. Evans C. R. Burant C. F. Starkl P. (2012). The sedoheptulose kinase CARKL directs macrophage polarization through control of glucose metabolism. Cell Metab. 15 (6), 813–826. 10.1016/j.cmet.2012.04.023 22682222 PMC3370649

[B81] Hazell L. J. Arnold L. Flowers D. Waeg G. Malle E. Stocker R. (1996). Presence of hypochlorite-modified proteins in human atherosclerotic lesions. J. Clin. Invest. 97 (6), 1535–1544. 10.1172/JCI118576 8617887 PMC507214

[B82] He Y. Liu T. (2023). Oxidized low-density lipoprotein regulates macrophage polarization in atherosclerosis. Int. Immunopharmacol. 120, 110338. 10.1016/j.intimp.2023.110338 37210916

[B83] Hirose T. Hamaguchi S. Matsumoto N. Irisawa T. Seki M. Tasaki O. (2014). Presence of neutrophil extracellular traps and citrullinated histone H3 in the bloodstream of critically ill patients. PLoS One 9 (11), e111755. 10.1371/journal.pone.0111755 25392950 PMC4230949

[B84] Hosokawa T. Kumon Y. Kobayashi T. Enzan H. Nishioka Y. Yuri K. (2011). Neutrophil infiltration and oxidant-production in human atherosclerotic carotid plaques. Histol. Histopathol. 26 (1), 1–11. 10.14670/HH-26.1 21117022

[B85] Huang H. Vandekeere S. Kalucka J. Bierhansl L. Zecchin A. Brüning U. (2017). Role of glutamine and interlinked asparagine metabolism in vessel formation. Embo J. 36 (16), 2334–2352. 10.15252/embj.201695518 28659375 PMC5556263

[B86] Huang S. Rutkowsky J. M. Snodgrass R. G. Ono-Moore K. D. Schneider D. A. Newman J. W. (2012). Saturated fatty acids activate TLR-mediated proinflammatory signaling pathways. J. Lipid Res. 53 (9), 2002–2013. 10.1194/jlr.D029546 22766885 PMC3413240

[B87] Huang S. C. Everts B. Ivanova Y. O'Sullivan D. Nascimento M. Smith A. M. (2014). Cell-intrinsic lysosomal lipolysis is essential for alternative activation of macrophages. Nat. Immunol. 15 (9), 846–855. 10.1038/ni.2956 25086775 PMC4139419

[B88] Huang S. C. Smith A. M. Everts B. Colonna M. Pearce E. L. Schilling J. D. (2016). Metabolic reprogramming mediated by the mTORC2-IRF4 signaling Axis is essential for macrophage alternative activation. Immunity 45 (4), 817–830. 10.1016/j.immuni.2016.09.016 27760338 PMC5535820

[B89] Hurtubise J. McLellan K. Durr K. Onasanya O. Nwabuko D. Ndisang J. F. (2016). The different facets of dyslipidemia and hypertension in atherosclerosis. Curr. Atheroscler. Rep. 18 (12), 82. 10.1007/s11883-016-0632-z 27822682

[B90] Hwang A. R. Nam J. O. Kang Y. J. (2018). Fluvastatin inhibits advanced glycation end products-induced proliferation, migration, and extracellular matrix accumulation in vascular smooth muscle cells by targeting connective tissue growth factor. Korean J. Physiol. Pharmacol. 22 (2), 193–201. 10.4196/kjpp.2018.22.2.193 29520172 PMC5840078

[B91] Ignarro L. J. Buga G. M. Wei L. H. Bauer P. M. Wu G. del Soldato P. (2001). Role of the arginine-nitric oxide pathway in the regulation of vascular smooth muscle cell proliferation. Proc. Natl. Acad. Sci. U. S. A. 98 (7), 4202–4208. 10.1073/pnas.071054698 11259671 PMC31203

[B92] Infantino V. Convertini P. Cucci L. Panaro M. A. Di Noia M. A. Calvello R. (2011). The mitochondrial citrate carrier: a new player in inflammation. Biochem. J. 438 (3), 433–436. 10.1042/BJ20111275 21787310

[B93] Inoguchi T. Li P. Umeda F. Yu H. Y. Kakimoto M. Imamura M. (2000). High glucose level and free fatty acid stimulate reactive oxygen species production through protein kinase C--dependent activation of NAD(P)H oxidase in cultured vascular cells. Diabetes 49 (11), 1939–1945. 10.2337/diabetes.49.11.1939 11078463

[B94] Jain M. Dhanesha N. Doddapattar P. Nayak M. K. Guo L. Cornelissen A. (2021). Smooth muscle cell-specific PKM2 (pyruvate kinase muscle 2) promotes smooth muscle cell phenotypic switching and neointimal hyperplasia. Arteriosclerosis, thrombosis, Vasc. Biol. 41 (5), 1724–1737. 10.1161/ATVBAHA.121.316021 PMC8062279 33691477

[B95] Jang C. Oh S. F. Wada S. Rowe G. C. Liu L. Chan M. C. (2016). A branched-chain amino acid metabolite drives vascular fatty acid transport and causes insulin resistance. Nat. Med. 22 (4), 421–426. 10.1038/nm.4057 26950361 PMC4949205

[B96] Jeon J. H. Hong C. W. Kim E. Y. Lee J. M. (2020). Current understanding on the metabolism of neutrophils. Immune Netw. 20 (6), e46. 10.4110/in.2020.20.e46 33425431 PMC7779868

[B97] Jha A. K. Huang S. C. Sergushichev A. Lampropoulou V. Ivanova Y. Loginicheva E. (2015). Network integration of parallel metabolic and transcriptional data reveals metabolic modules that regulate macrophage polarization. Immunity 42 (3), 419–430. 10.1016/j.immuni.2015.02.005 25786174

[B98] Jones R. G. Pearce E. J. (2017). MenTORing immunity: mTOR signaling in the development and function of tissue-resident immune cells. Immunity 46 (5), 730–742. 10.1016/j.immuni.2017.04.028 28514674 PMC5695239

[B99] Josefs T. Barrett T. J. Brown E. J. Quezada A. Wu X. Voisin M. (2020). Neutrophil extracellular traps promote macrophage inflammation and impair atherosclerosis resolution in diabetic mice. JCI Insight 5 (7), e134796. 10.1172/jci.insight.134796 32191637 PMC7205252

[B100] Joshi M. B. Ahamed R. Hegde M. Nair A. S. Ramachandra L. Satyamoorthy K. (2020). Glucose induces metabolic reprogramming in neutrophils during type 2 diabetes to form constitutive extracellular traps and decreased responsiveness to lipopolysaccharides. Biochim. Biophys. Acta Mol. Basis Dis. 1866 (12), 165940. 10.1016/j.bbadis.2020.165940 32827651

[B101] Judge A. Dodd M. S. (2020). Metabolism. Essays Biochem. 64 (4), 607–647. 10.1042/EBC20190041 32830223 PMC7545035

[B102] Kannan K. B. Barlos D. Hauser C. J. (2007). Free cholesterol alters lipid raft structure and function regulating neutrophil Ca2+ entry and respiratory burst: correlations with calcium channel raft trafficking. J. Immunol. 178 (8), 5253–5261. 10.4049/jimmunol.178.8.5253 17404309

[B103] Kataoka H. Kume N. Miyamoto S. Minami M. Morimoto M. Hayashida K. (2001). Oxidized LDL modulates Bax/Bcl-2 through the lectinlike Ox-LDL receptor-1 in vascular smooth muscle cells. Arteriosclerosis, thrombosis, Vasc. Biol. 21 (6), 955–960. 10.1161/01.atv.21.6.955 11397703

[B104] Keating S. T. Groh L. Thiem K. Bekkering S. Li Y. Matzaraki V. (2020). Rewiring of glucose metabolism defines trained immunity induced by oxidized low-density lipoprotein. J. Mol. Med. Berl. 98 (6), 819–831. 10.1007/s00109-020-01915-w 32350546 PMC7297856

[B105] Kedia-Mehta N. Finlay D. K. (2019). Competition for nutrients and its role in controlling immune responses. Nat. Commun. 10 (1), 2123. 10.1038/s41467-019-10015-4 31073180 PMC6509329

[B106] Khallou-Laschet J. Varthaman A. Fornasa G. Compain C. Gaston A. T. Clement M. (2010). Macrophage plasticity in experimental atherosclerosis. PLoS One 5 (1), e8852. 10.1371/journal.pone.0008852 20111605 PMC2810335

[B107] Kieler M. Hofmann M. Schabbauer G. (2021). More than just protein building blocks: how amino acids and related metabolic pathways fuel macrophage polarization. Febs J. 288 (12), 3694–3714. 10.1111/febs.15715 33460504 PMC8359336

[B108] Kim J. Kim Y. H. Kim J. Park D. Y. Bae H. Lee D. H. (2017). YAP/TAZ regulates sprouting angiogenesis and vascular barrier maturation. J. Clin. Invest. 127 (9), 3441–3461. 10.1172/JCI93825 28805663 PMC5669570

[B109] Kim J. B. Zhao Q. Nguyen T. Pjanic M. Cheng P. Wirka R. (2020). Environment-sensing aryl hydrocarbon receptor inhibits the chondrogenic fate of modulated smooth muscle cells in atherosclerotic lesions. Circulation 142 (6), 575–590. 10.1161/CIRCULATIONAHA.120.045981 32441123 PMC8066499

[B110] Klysz D. Tai X. Robert P. A. Craveiro M. Cretenet G. Oburoglu L. (2015). Glutamine-dependent α-ketoglutarate production regulates the balance between T helper 1 cell and regulatory T cell generation. Sci. Signal 8 (396), ra97. 10.1126/scisignal.aab2610 26420908

[B111] Koelwyn G. J. Corr E. M. Erbay E. Moore K. J. (2018). Regulation of macrophage immunometabolism in atherosclerosis. Nat. Immunol. 19 (6), 526–537. 10.1038/s41590-018-0113-3 29777212 PMC6314674

[B112] Kopin L. Lowenstein C. (2017). Dyslipidemia. Ann. Intern Med. 167 (11), Itc81–itc96. 10.7326/AITC201712050 29204622

[B113] Krützfeldt A. Spahr R. Mertens S. Siegmund B. Piper H. M. (1990). Metabolism of exogenous substrates by coronary endothelial cells in culture. J. Mol. Cell Cardiol. 22 (12), 1393–1404. 10.1016/0022-2828(90)90984-a 2089157

[B114] Kumar S. Dikshit M. (2019). Metabolic insight of neutrophils in health and disease. Front. Immunol. 10, 2099. 10.3389/fimmu.2019.02099 31616403 PMC6764236

[B115] Kuo A. Lee M. Y. Sessa W. C. (2017). Lipid droplet biogenesis and function in the endothelium. Circ. Res. 120 (8), 1289–1297. 10.1161/CIRCRESAHA.116.310498 28119423 PMC5392152

[B116] Lauterbach M. A. Hanke J. E. Serefidou M. Mangan M. S. J. Kolbe C. C. Hess T. (2019). Toll-like receptor signaling rewires macrophage metabolism and promotes histone acetylation via ATP-citrate lyase. Immunity 51 (6), 997–1011. 10.1016/j.immuni.2019.11.009 31851905

[B117] Lee S. J. Thien Quach C. H. Jung K. H. Paik J. Y. Lee J. H. Park J. W. (2014). Oxidized low-density lipoprotein stimulates macrophage 18F-FDG uptake via hypoxia-inducible factor-1α activation through Nox2-dependent reactive oxygen species generation. J. Nucl. Med. 55 (10), 1699–1705. 10.2967/jnumed.114.139428 25214643

[B118] Legrand-Poels S. Esser N. L'Homme L. Scheen A. Paquot N. Piette J. (2014). Free fatty acids as modulators of the NLRP3 inflammasome in obesity/type 2 diabetes. Biochem. Pharmacol. 92 (1), 131–141. 10.1016/j.bcp.2014.08.013 25175736

[B119] Lehn-Stefan A. Peter A. Machann J. Schick F. Randrianarisoa E. Heni M. (2021). Elevated circulating glutamate is associated with subclinical atherosclerosis independently of established risk markers: a cross-sectional study. J. Clin. Endocrinol. Metab. 106 (2), e982–e989. 10.1210/clinem/dgaa898 33277657

[B120] Leopold J. A. Walker J. Scribner A. W. Voetsch B. Zhang Y. Y. Loscalzo A. J. (2003). Glucose-6-phosphate dehydrogenase modulates vascular endothelial growth factor-mediated angiogenesis. J. Biol. Chem. 278 (34), 32100–32106. 10.1074/jbc.M301293200 12777375

[B121] Li X. Sun X. Carmeliet P. (2019). Hallmarks of endothelial cell metabolism in health and disease. Cell Metab. 30 (3), 414–433. 10.1016/j.cmet.2019.08.011 31484054

[B122] Liao X. Sharma N. Kapadia F. Zhou G. Lu Y. Hong H. (2011). Krüppel-like factor 4 regulates macrophage polarization. J. Clin. Invest. 121 (7), 2736–2749. 10.1172/JCI45444 21670502 PMC3223832

[B123] Liao X. Sluimer J. C. Wang Y. Subramanian M. Brown K. Pattison J. S. (2012). Macrophage autophagy plays a protective role in advanced atherosclerosis. Cell Metab. 15 (4), 545–553. 10.1016/j.cmet.2012.01.022 22445600 PMC3322248

[B124] Libby P. (2021). The changing landscape of atherosclerosis. Nature 592 (7855), 524–533. 10.1038/s41586-021-03392-8 33883728

[B125] Libby P. Pasterkamp G. Crea F. Jang I. K. (2019). Reassessing the mechanisms of acute coronary syndromes. Circ. Res. 124 (1), 150–160. 10.1161/CIRCRESAHA.118.311098 30605419 PMC6447371

[B126] Lim C. H. Adav S. S. Sze S. K. Choong Y. K. Saravanan R. Schmidtchen A. (2018). Thrombin and plasmin alter the proteome of neutrophil extracellular traps. Front. Immunol. 9, 1554. 10.3389/fimmu.2018.01554 30038618 PMC6046383

[B127] Liu P. S. Wang H. Li X. Chao T. Teav T. Christen S. (2017). α-ketoglutarate orchestrates macrophage activation through metabolic and epigenetic reprogramming. Nat. Immunol. 18 (9), 985–994. 10.1038/ni.3796 28714978

[B128] Locher R. Brandes R. P. Vetter W. Barton M. (2002). Native LDL induces proliferation of human vascular smooth muscle cells via redox-mediated activation of ERK 1/2 mitogen-activated protein kinases. Hypertension 39 (2 Pt 2), 645–650. 10.1161/hy0202.103473 11882624

[B129] Longchamp A. Mirabella T. Arduini A. MacArthur M. R. Das A. Treviño-Villarreal J. H. (2018). Amino acid restriction triggers angiogenesis via GCN2/ATF4 regulation of VEGF and H(2)S production. Cell 173 (1), 117–129. 10.1016/j.cell.2018.03.001 29570992 PMC5901681

[B130] Lunt S. Y. Vander Heiden M. G. (2011). Aerobic glycolysis: meeting the metabolic requirements of cell proliferation. Annu. Rev. Cell Dev. Biol. 27, 441–464. 10.1146/annurev-cellbio-092910-154237 21985671

[B131] Luo J. Yang H. Song B. L. (2020). Mechanisms and regulation of cholesterol homeostasis. Nat. Rev. Mol. Cell Biol. 21 (4), 225–245. 10.1038/s41580-019-0190-7 31848472

[B132] Lynch R. M. Paul R. J. (1983). Compartmentation of glycolytic and glycogenolytic metabolism in vascular smooth muscle. Science 222 (4630), 1344–1346. 10.1126/science.6658455 6658455

[B133] Ma S. Yang D. Li D. Tang B. Yang Y. (2011). Oleic acid induces smooth muscle foam cell formation and enhances atherosclerotic lesion development via CD36. Lipids Health Dis. 10, 53. 10.1186/1476-511X-10-53 21486455 PMC3083368

[B134] Maianski N. A. Geissler J. Srinivasula S. M. Alnemri E. S. Roos D. Kuijpers T. W. (2004). Functional characterization of mitochondria in neutrophils: a role restricted to apoptosis. Cell Death Differ. 11 (2), 143–153. 10.1038/sj.cdd.4401320 14576767

[B135] Mann G. E. Yudilevich D. L. Sobrevia L. (2003). Regulation of amino acid and glucose transporters in endothelial and smooth muscle cells. Physiol. Rev. 83 (1), 183–252. 10.1152/physrev.00022.2002 12506130

[B136] Marsch E. Theelen T. L. Demandt J. A. Jeurissen M. van Gink M. Verjans R. (2014). Reversal of hypoxia in murine atherosclerosis prevents necrotic core expansion by enhancing efferocytosis. Arteriosclerosis, thrombosis, Vasc. Biol. 34 (12), 2545–2553. 10.1161/ATVBAHA.114.304023 25256233

[B137] Martínez-Reyes I. Chandel N. S. (2020). Mitochondrial TCA cycle metabolites control physiology and disease. Nat. Commun. 11 (1), 102. 10.1038/s41467-019-13668-3 31900386 PMC6941980

[B138] Matoba A. Matsuyama N. Shibata S. Masaki E. Emala C. W. Mizuta K. (2018). The free fatty acid receptor 1 promotes airway smooth muscle cell proliferation through MEK/ERK and PI3K/Akt signaling pathways. Am. J. Physiol. Lung Cell Mol. Physiol. 314 (3), L333–L348. 10.1152/ajplung.00129.2017 29097424 PMC5900353

[B139] Matsuura Y. Shimizu-Albergine M. Barnhart S. Kramer F. Hsu C. C. Kothari V. (2022). Diabetes suppresses glucose uptake and glycolysis in macrophages. Circ. Res. 130 (5), 779–781. 10.1161/CIRCRESAHA.121.320060 35170337 PMC8897241

[B140] Meegan J. E. Yang X. Coleman D. C. Jannaway M. Yuan S. Y. (2017). Neutrophil-mediated vascular barrier injury: role of neutrophil extracellular traps. Microcirc. (New York, N. Y. 1994) 24 (3). 10.1111/micc.12352 PMC5404986 28120468

[B141] Megens R. T. Vijayan S. Lievens D. Döring Y. van Zandvoort M. A. Grommes J. (2012). Presence of luminal neutrophil extracellular traps in atherosclerosis. Thromb. Haemost. 107 (3), 597–598. 10.1160/TH11-09-0650 22318427

[B142] Mehrotra D. Wu J. Papangeli I. Chun H. J. (2014). Endothelium as a gatekeeper of fatty acid transport. Trends Endocrinol. Metab. 25 (2), 99–106. 10.1016/j.tem.2013.11.001 24315207 PMC3946743

[B143] Mehta M. M. Weinberg S. E. Chandel N. S. (2017). Mitochondrial control of immunity: beyond ATP. Nat. Rev. Immunol. 17 (10), 608–620. 10.1038/nri.2017.66 28669986

[B144] Merchan J. R. Kovács K. Railsback J. W. Kurtoglu M. Jing Y. Piña Y. (2010). Antiangiogenic activity of 2-deoxy-D-glucose. PLoS One 5 (10), e13699. 10.1371/journal.pone.0013699 21060881 PMC2965179

[B145] Merlin J. Ivanov S. Dumont A. Sergushichev A. Gall J. Stunault M. (2021). Non-canonical glutamine transamination sustains efferocytosis by coupling redox buffering to oxidative phosphorylation. Nat. Metab. 3 (10), 1313–1326. 10.1038/s42255-021-00471-y 34650273 PMC7611882

[B146] Meuwese M. C. Stroes E. S. Hazen S. L. van Miert J. N. Kuivenhoven J. A. Schaub R. G. (2007). Serum myeloperoxidase levels are associated with the future risk of coronary artery disease in apparently healthy individuals: the EPIC-Norfolk Prospective Population Study. J. Am. Coll. Cardiol. 50 (2), 159–165. 10.1016/j.jacc.2007.03.033 17616301

[B147] Michelakis E. D. Weir E. K. (2008). The metabolic basis of vascular oxygen sensing: diversity, compartmentalization, and lessons from cancer. Am. J. physiology Heart circulatory physiology 295 (3), H928–H30. 10.1152/ajpheart.00697.2008 PMC2544482 18621852

[B148] Michiels C. F. Kurdi A. Timmermans J. P. De Meyer G. R. Y. Martinet W. (2016). Spermidine reduces lipid accumulation and necrotic core formation in atherosclerotic plaques via induction of autophagy. Atherosclerosis 251, 319–327. 10.1016/j.atherosclerosis.2016.07.899 27450786

[B149] Mills E. L. Kelly B. Logan A. Costa A. S. H. Varma M. Bryant C. E. (2016). Succinate dehydrogenase supports metabolic repurposing of mitochondria to drive inflammatory macrophages. Cell 167 (2), 457–470. 10.1016/j.cell.2016.08.064 27667687 PMC5863951

[B150] Moon J. S. Lee S. Park M. A. Siempos I. I. Haslip M. Lee P. J. (2023). UCP2-induced fatty acid synthase promotes NLRP3 inflammasome activation during sepsis. J. Clin. Invest. 133 (6), e169986. 10.1172/JCI169986 36919700 PMC10014094

[B151] Moore K. J. Sheedy F. J. Fisher E. A. (2013). Macrophages in atherosclerosis: a dynamic balance. Nat. Rev. Immunol. 13 (10), 709–721. 10.1038/nri3520 23995626 PMC4357520

[B152] Namgaladze D. Brüne B. (2014). Fatty acid oxidation is dispensable for human macrophage IL-4-induced polarization. Biochimica biophysica acta 1841 (9), 1329–1335. 10.1016/j.bbalip.2014.06.007 24960101

[B153] Namgaladze D. Brüne B. (2016). Macrophage fatty acid oxidation and its roles in macrophage polarization and fatty acid-induced inflammation. Biochim. Biophys. Acta 1861 (11), 1796–1807. 10.1016/j.bbalip.2016.09.002 27614008

[B154] Newman A. A. C. Serbulea V. Baylis R. A. Shankman L. S. Bradley X. Alencar G. F. (2021). Multiple cell types contribute to the atherosclerotic lesion fibrous cap by PDGFRβ and bioenergetic mechanisms. Nat. Metab. 3 (2), 166–181. 10.1038/s42255-020-00338-8 33619382 PMC7905710

[B155] Nitz K. Lacy M. Atzler D. (2019). Amino acids and their metabolism in atherosclerosis. Arteriosclerosis, thrombosis, Vasc. Biol. 39 (3), 319–330. 10.1161/ATVBAHA.118.311572 30650999

[B156] Noels H. Jankowski J. (2023). Increased risk of cardiovascular complications in chronic kidney disease: introduction to a compendium. Circ. Res. 132 (8), 899–901. 10.1161/CIRCRESAHA.123.322806 37053281

[B157] Noels H. Lehrke M. Vanholder R. Jankowski J. (2021). Lipoproteins and fatty acids in chronic kidney disease: molecular and metabolic alterations. Nat. Rev. Nephrol. 17 (8), 528–542. 10.1038/s41581-021-00423-5 33972752

[B158] Nomura M. Liu J. Rovira I. I. Gonzalez-Hurtado E. Lee J. Wolfgang M. J. (2016). Fatty acid oxidation in macrophage polarization. Nat. Immunol. 17 (3), 216–217. 10.1038/ni.3366 26882249 PMC6033271

[B159] Nordestgaard B. G. (2016). Triglyceride-rich lipoproteins and atherosclerotic cardiovascular disease: new insights from epidemiology, genetics, and biology. Circ. Res. 118 (4), 547–563. 10.1161/CIRCRESAHA.115.306249 26892957

[B160] Nus M. Mallat Z. (2016). Immune-mediated mechanisms of atherosclerosis and implications for the clinic. Expert Rev. Clin. Immunol. 12 (11), 1217–1237. 10.1080/1744666X.2016.1195686 27253721

[B161] Omori K. Ohira T. Uchida Y. Ayilavarapu S. Batista E. L. Jr Yagi M. (2008). Priming of neutrophil oxidative burst in diabetes requires preassembly of the NADPH oxidase. J. Leukoc. Biol. 84 (1), 292–301. 10.1189/jlb.1207832 18390927 PMC2774791

[B162] O’Neill L. A. Hardie D. G. (2013). Metabolism of inflammation limited by AMPK and pseudo-starvation. Nature 493 (7432), 346–355. 10.1038/nature11862 23325217

[B163] O’Neill L. A. Kishton R. J. Rathmell J. (2016). A guide to immunometabolism for immunologists. Nat. Rev. Immunol. 16 (9), 553–565. 10.1038/nri.2016.70 27396447 PMC5001910

[B164] O’Rourke S. A. Neto N. G. B. Devilly E. Shanley L. C. Fitzgerald H. K. Monaghan M. G. (2022). Cholesterol crystals drive metabolic reprogramming and M1 macrophage polarisation in primary human macrophages. Atherosclerosis 352, 35–45. 10.1016/j.atherosclerosis.2022.05.015 35667162

[B165] Ouyang L. Yu C. Xie Z. Su X. Xu Z. Song P. (2022). Indoleamine 2,3-dioxygenase 1 deletion-mediated kynurenine insufficiency in vascular smooth muscle cells exacerbates arterial calcification. Circulation 145 (24), 1784–1798. 10.1161/CIRCULATIONAHA.121.057868 35582948 PMC9197997

[B166] Papa S. Choy P. M. Bubici C. (2019). The ERK and JNK pathways in the regulation of metabolic reprogramming. Oncogene 38 (13), 2223–2240. 10.1038/s41388-018-0582-8 30487597 PMC6398583

[B167] Park H. Y. Kim M. J. Kim Y. J. Lee S. Jin J. Lee S. (2021). V-9302 inhibits proliferation and migration of VSMCs, and reduces neointima formation in mice after carotid artery ligation. Biochem. Biophys. Res. Commun. 560, 45–51. 10.1016/j.bbrc.2021.04.079 33965788

[B168] Patella F. Schug Z. T. Persi E. Neilson L. J. Erami Z. Avanzato D. (2015). Proteomics-based metabolic modeling reveals that fatty acid oxidation (FAO) controls endothelial cell (EC) permeability. Mol. Cell Proteomics 14 (3), 621–634. 10.1074/mcp.M114.045575 25573745 PMC4349982

[B169] Paulin N. Viola J. R. Maas S. L. de Jong R. Fernandes-Alnemri T. Weber C. (2018). Double-strand DNA sensing Aim2 inflammasome regulates atherosclerotic plaque vulnerability. Circulation 138 (3), 321–323. 10.1161/CIRCULATIONAHA.117.033098 30012706

[B170] Peace C. G. O’Neill L. A. (2022). The role of itaconate in host defense and inflammation. J. Clin. Invest. 132 (2), e148548. 10.1172/JCI148548 35040439 PMC8759771

[B171] Peiró C. Romacho T. Azcutia V. Villalobos L. Fernández E. Bolaños J. P. (2016). Inflammation, glucose, and vascular cell damage: the role of the pentose phosphate pathway. Cardiovasc Diabetol. 15, 82. 10.1186/s12933-016-0397-2 27245224 PMC4888494

[B172] Perez J. Hill B. G. Benavides G. A. Dranka B. P. Darley-Usmar V. M. (2010). Role of cellular bioenergetics in smooth muscle cell proliferation induced by platelet-derived growth factor. Biochem. J. 428 (2), 255–267. 10.1042/BJ20100090 20331438 PMC3641000

[B173] Perry R. N. Albarracin D. Aherrahrou R. Civelek M. (2023). Network preservation analysis reveals dysregulated metabolic pathways in human vascular smooth muscle cell phenotypic switching. Circ. Genom Precis. Med. 16 (4), 372–381. 10.1161/CIRCGEN.122.003781 37387208 PMC10434832

[B174] Pertiwi K. R. van der Wal A. C. Pabittei D. R. Mackaaij C. van Leeuwen M. B. Li X. (2018). Neutrophil extracellular traps participate in all different types of thrombotic and haemorrhagic complications of coronary atherosclerosis. Thromb. Haemost. 118 (6), 1078–1087. 10.1055/s-0038-1641749 29672788

[B175] Petty H. R. Kindzelskii A. L. Chaiworapongsa T. Petty A. R. Romero R. (2005). Oxidant release is dramatically increased by elevated glucose concentrations in neutrophils from pregnant women. J. Matern. Fetal Neonatal Med. 18 (6), 397–404. 10.1080/14767050500361679 16390806

[B176] Poznyak A. Grechko A. V. Poggio P. Myasoedova V. A. Alfieri V. Orekhov A. N. (2020). The diabetes mellitus-atherosclerosis connection: the role of lipid and glucose metabolism and chronic inflammation. Int. J. Mol. Sci. 21 (5), 1835. 10.3390/ijms21051835 32155866 PMC7084712

[B177] Quan J. Liu J. Gao X. Liu J. Yang H. Chen W. (2014). Palmitate induces interleukin-8 expression in human aortic vascular smooth muscle cells via Toll-like receptor 4/nuclear factor-κB pathway (TLR4/NF-κB-8). J. Diabetes 6 (1), 33–41. 10.1111/1753-0407.12073 23826669

[B178] Ren W. Xia Y. Chen S. Wu G. Bazer F. W. Zhou B. (2019). Glutamine metabolism in macrophages: a novel target for obesity/type 2 diabetes. Adv. Nutr. 10 (2), 321–330. 10.1093/advances/nmy084 30753258 PMC6416106

[B179] Rhoads J. P. Major A. S. Rathmell J. C. (2017). Fine tuning of immunometabolism for the treatment of rheumatic diseases. Nat. Rev. Rheumatol. 13 (5), 313–320. 10.1038/nrrheum.2017.54 28381829 PMC5502208

[B180] Rice C. M. Davies L. C. Subleski J. J. Maio N. Gonzalez-Cotto M. Andrews C. (2018). Tumour-elicited neutrophils engage mitochondrial metabolism to circumvent nutrient limitations and maintain immune suppression. Nat. Commun. 9 (1), 5099. 10.1038/s41467-018-07505-2 30504842 PMC6269473

[B181] Ridker P. M. Everett B. M. Thuren T. MacFadyen J. G. Chang W. H. Ballantyne C. (2017). Antiinflammatory therapy with canakinumab for atherosclerotic disease. N. Engl. J. Med. 377 (12), 1119–1131. 10.1056/nejmoa1707914 28845751

[B182] Riffelmacher T. Clarke A. Richter F. C. Stranks A. Pandey S. Danielli S. (2017). Autophagy-dependent generation of free fatty acids is critical for normal neutrophil differentiation. Immunity 47 (3), 466–480. 10.1016/j.immuni.2017.08.005 28916263 PMC5610174

[B183] Robinson J. M. Karnovsky M. L. Karnovsky M. J. (1982). Glycogen accumulation in polymorphonuclear leukocytes, and other intracellular alterations that occur during inflammation. J. Cell Biol. 95 (3), 933–942. 10.1083/jcb.95.3.933 7153252 PMC2112917

[B184] Rodriguez A. E. Ducker G. S. Billingham L. K. Martinez C. A. Mainolfi N. Suri V. (2019). Serine metabolism supports macrophage IL-1β production. Cell Metab. 29 (4), 1003–1011. 10.1016/j.cmet.2019.01.014 30773464 PMC6447453

[B185] Rodríguez-Espinosa O. Rojas-Espinosa O. Moreno-Altamirano M. M. López-Villegas E. O. Sánchez-García F. J. (2015). Metabolic requirements for neutrophil extracellular traps formation. Immunology 145 (2), 213–224. 10.1111/imm.12437 25545227 PMC4427386

[B186] Rodríguez-Prados J. C. Través P. G. Cuenca J. Rico D. Aragonés J. Martín-Sanz P. (2010). Substrate fate in activated macrophages: a comparison between innate, classic, and alternative activation. J. Immunol. 185 (1), 605–614. 10.4049/jimmunol.0901698 20498354

[B187] Rom O. Grajeda-Iglesias C. Najjar M. Abu-Saleh N. Volkova N. Dar D. E. (2017). Atherogenicity of amino acids in the lipid-laden macrophage model system *in vitro* and in atherosclerotic mice: a key role for triglyceride metabolism. J. Nutr. Biochem. 45, 24–38. 10.1016/j.jnutbio.2017.02.023 28431321

[B188] Rom O. Liu Y. Finney A. C. Ghrayeb A. Zhao Y. Shukha Y. (2022). Induction of glutathione biosynthesis by glycine-based treatment mitigates atherosclerosis. Redox Biol. 52, 102313. 10.1016/j.redox.2022.102313 35447412 PMC9044008

[B189] Roth G. A. Mensah G. A. Johnson C. O. Addolorato G. Ammirati E. Baddour L. M. (2020). Global burden of cardiovascular diseases and risk factors, 1990-2019: update from the GBD 2019 study. J. Am. Coll. Cardiol. 76 (25), 2982–3021. 10.1016/j.jacc.2020.11.010 33309175 PMC7755038

[B190] Roy P. Orecchioni M. Ley K. (2022). How the immune system shapes atherosclerosis: roles of innate and adaptive immunity. Nat. Rev. Immunol. 22 (4), 251–265. 10.1038/s41577-021-00584-1 34389841 PMC10111155

[B191] Rudd J. H. Warburton E. A. Fryer T. D. Jones H. A. Clark J. C. Antoun N. (2002). Imaging atherosclerotic plaque inflammation with [18F]-fluorodeoxyglucose positron emission tomography. Circulation 105 (23), 2708–2711. 10.1161/01.cir.0000020548.60110.76 12057982

[B192] Ryan D. G. O’Neill L. A. J. (2020). Krebs cycle reborn in macrophage immunometabolism. Annu. Rev. Immunol. 38, 289–313. 10.1146/annurev-immunol-081619-104850 31986069

[B193] Sage A. P. Tsiantoulas D. Binder C. J. Mallat Z. (2019). The role of B cells in atherosclerosis. Nat. Rev. Cardiol. 16 (3), 180–196. 10.1038/s41569-018-0106-9 30410107

[B194] Saigusa R. Winkels H. Ley K. (2020). T cell subsets and functions in atherosclerosis. Nat. Rev. Cardiol. 17 (7), 387–401. 10.1038/s41569-020-0352-5 32203286 PMC7872210

[B195] Salabei J. K. Hill B. G. (2013). Mitochondrial fission induced by platelet-derived growth factor regulates vascular smooth muscle cell bioenergetics and cell proliferation. Redox Biol. 1 (1), 542–551. 10.1016/j.redox.2013.10.011 24273737 PMC3836280

[B196] Sano H. Sudo T. Yokode M. Murayama T. Kataoka H. Takakura N. (2001). Functional blockade of platelet-derived growth factor receptor-beta but not of receptor-alpha prevents vascular smooth muscle cell accumulation in fibrous cap lesions in apolipoprotein E-deficient mice. Circulation 103 (24), 2955–2960. 10.1161/01.cir.103.24.2955 11413086

[B197] Sarkar O. Li Y. Anand-Srivastava M. B. (2017). Nitric oxide attenuates overexpression of Giα proteins in vascular smooth muscle cells from SHR: role of ROS and ROS-mediated signaling. PLoS One 12 (7), e0179301. 10.1371/journal.pone.0179301 28692698 PMC5503203

[B198] Saxton R. A. Sabatini D. M. (2017). mTOR signaling in growth, metabolism, and disease. Cell 168 (6), 960–976. 10.1016/j.cell.2017.02.004 28283069 PMC5394987

[B199] Schilperoort M. Ngai D. Katerelos M. Power D. A. Tabas I. (2023). PFKFB2-mediated glycolysis promotes lactate-driven continual efferocytosis by macrophages. Nat. Metab. 5 (3), 431–444. 10.1038/s42255-023-00736-8 36797420 PMC10050103

[B200] Schnyder G. Roffi M. Pin R. Flammer Y. Lange H. Eberli F. R. (2001). Decreased rate of coronary restenosis after lowering of plasma homocysteine levels. N. Engl. J. Med. 345 (22), 1593–1600. 10.1056/NEJMoa011364 11757505

[B201] Schoors S. Bruning U. Missiaen R. Queiroz K. C. Borgers G. Elia I. (2015). Fatty acid carbon is essential for dNTP synthesis in endothelial cells. Nature 520 (7546), 192–197. 10.1038/nature14362 25830893 PMC4413024

[B202] Schoors S. De Bock K. Cantelmo A. R. Georgiadou M. Ghesquière B. Cauwenberghs S. (2014). Partial and transient reduction of glycolysis by PFKFB3 blockade reduces pathological angiogenesis. Cell Metab. 19 (1), 37–48. 10.1016/j.cmet.2013.11.008 24332967

[B203] Seeley E. H. Liu Z. Yuan S. Stroope C. Cockerham E. Rashdan N. A. (2023). Spatially resolved metabolites in stable and unstable human atherosclerotic plaques identified by mass spectrometry imaging. Arteriosclerosis, thrombosis, Vasc. Biol. 43 (9), 1626–1635. 10.1161/ATVBAHA.122.318684 PMC10527524 37381983

[B204] Selvin E. Coresh J. Shahar E. Zhang L. Steffes M. Sharrett A. R. (2005). Glycaemia (haemoglobin A1c) and incident ischaemic stroke: the atherosclerosis risk in communities (ARIC) study. Lancet Neurol. 4 (12), 821–826. 10.1016/S1474-4422(05)70227-1 16297840

[B205] Shah M. S. Brownlee M. (2016). Molecular and cellular mechanisms of cardiovascular disorders in diabetes. Circ. Res. 118 (11), 1808–1829. 10.1161/CIRCRESAHA.116.306923 27230643 PMC4888901

[B206] Shan X. Hu P. Ni L. Shen L. Zhang Y. Ji Z. (2022). Serine metabolism orchestrates macrophage polarization by regulating the IGF1-p38 axis. Cell Mol. Immunol. 19 (11), 1263–1278. 10.1038/s41423-022-00925-7 36180780 PMC9622887

[B207] Shi H. Kokoeva M. V. Inouye K. Tzameli I. Yin H. Flier J. S. (2006). TLR4 links innate immunity and fatty acid-induced insulin resistance. J. Clin. Invest. 116 (11), 3015–3025. 10.1172/JCI28898 17053832 PMC1616196

[B208] Shimabukuro M. (2022). Serotonin and atheroscelotic cardiovascular disease. J. Atheroscler. Thromb. 29 (3), 315–316. 10.5551/jat.ED182 34162787 PMC8894109

[B209] Shirai T. Nazarewicz R. R. Wallis B. B. Yanes R. E. Watanabe R. Hilhorst M. (2016). The glycolytic enzyme PKM2 bridges metabolic and inflammatory dysfunction in coronary artery disease. J. Exp. Med. 213 (3), 337–354. 10.1084/jem.20150900 26926996 PMC4813677

[B210] Silvestre-Roig C. Braster Q. Wichapong K. Lee E. Y. Teulon J. M. Berrebeh N. (2019). Externalized histone H4 orchestrates chronic inflammation by inducing lytic cell death. Nature 569 (7755), 236–240. 10.1038/s41586-019-1167-6 31043745 PMC6716525

[B211] Singh U. Devaraj S. Jialal I. (2009). C-reactive protein stimulates myeloperoxidase release from polymorphonuclear cells and monocytes: implications for acute coronary syndromes. Clin. Chem. 55 (2), 361–364. 10.1373/clinchem.2008.109207 19074520 PMC2662851

[B212] Škovierová H. Vidomanová E. Mahmood S. Sopková J. Drgová A. Červeňová T. (2016). The molecular and cellular effect of homocysteine metabolism imbalance on human health. Int. J. Mol. Sci. 17 (10), 1733. 10.3390/ijms17101733 27775595 PMC5085763

[B213] Soehnlein O. (2012). Multiple roles for neutrophils in atherosclerosis. Circ. Res. 110 (6), 875–888. 10.1161/CIRCRESAHA.111.257535 22427325

[B214] Soehnlein O. Weber C. Lindbom L. (2009). Neutrophil granule proteins tune monocytic cell function. Trends Immunol. 30 (11), 538–546. 10.1016/j.it.2009.06.006 19699683

[B215] Sohrabi Y. Lagache S. M. M. Schnack L. Godfrey R. Kahles F. Bruemmer D. (2018). mTOR-dependent oxidative stress regulates oxLDL-induced trained innate immunity in human monocytes. Front. Immunol. 9, 3155. 10.3389/fimmu.2018.03155 30723479 PMC6350618

[B216] Sohrabi Y. Sonntag G. V. H. Braun L. C. Lagache S. M. M. Liebmann M. Klotz L. (2020). LXR activation induces a proinflammatory trained innate immunity-phenotype in human monocytes. Front. Immunol. 11, 353. 10.3389/fimmu.2020.00353 32210962 PMC7077358

[B217] Song J. Zhang Y. Frieler R. A. Andren A. Wood S. Tyrrell D. J. (2023). Itaconate suppresses atherosclerosis by activating a Nrf2-dependent antiinflammatory response in macrophages in mice. J. Clin. Invest. 134 (3), e173034. 10.1172/JCI173034 38085578 PMC10849764

[B218] Soppert J. Lehrke M. Marx N. Jankowski J. Noels H. (2020). Lipoproteins and lipids in cardiovascular disease: from mechanistic insights to therapeutic targeting. Adv. Drug Deliv. Rev. 159, 4–33. 10.1016/j.addr.2020.07.019 32730849

[B219] Speer T. Dimmeler S. Schunk S. J. Fliser D. Ridker P. M. (2022). Targeting innate immunity-driven inflammation in CKD and cardiovascular disease. Nat. Rev. Nephrol. 18 (12), 762–778. 10.1038/s41581-022-00621-9 36064794

[B220] Sreejit G. Johnson J. Jaggers R. M. Dahdah A. Murphy A. J. Hanssen N. M. J. (2022). Neutrophils in cardiovascular disease: warmongers, peacemakers, or both? Cardiovasc Res. 118 (12), 2596–2609. 10.1093/cvr/cvab302 34534269 PMC9890471

[B221] Stanton R. C. (2012). Glucose-6-phosphate dehydrogenase, NADPH, and cell survival. IUBMB Life. 64 (5), 362–369. 10.1002/iub.1017 22431005 PMC3325335

[B222] Strelko C. L. Lu W. Dufort F. J. Seyfried T. N. Chiles T. C. Rabinowitz J. D. (2011). Itaconic acid is a mammalian metabolite induced during macrophage activation. J. Am. Chem. Soc. 133 (41), 16386–16389. 10.1021/ja2070889 21919507 PMC3216473

[B223] Stroope C. Nettersheim F. S. Coon B. Finney A. C. Schwartz M. A. Ley K. (2024). Dysregulated cellular metabolism in atherosclerosis: mediators and therapeutic opportunities. Nat. Metab. 6 (4), 617–638. 10.1038/s42255-024-01015-w 38532071 PMC11055680

[B224] Su S. C. Hung Y. J. Huang C. L. Shieh Y. S. Chien C. Y. Chiang C. F. (2019). Cilostazol inhibits hyperglucose-induced vascular smooth muscle cell dysfunction by modulating the RAGE/ERK/NF-κB signaling pathways. J. Biomed. Sci. 26 (1), 68. 10.1186/s12929-019-0550-9 31492153 PMC6731603

[B225] Tabas I. (2010). The role of endoplasmic reticulum stress in the progression of atherosclerosis. Circ. Res. 107 (7), 839–850. 10.1161/CIRCRESAHA.110.224766 20884885 PMC2951143

[B226] Tabas I. Bornfeldt K. E. (2020). Intracellular and intercellular aspects of macrophage immunometabolism in atherosclerosis. Circ. Res. 126 (9), 1209–1227. 10.1161/CIRCRESAHA.119.315939 32324504 PMC7392397

[B227] Tabas I. García-Cardeña G. Owens G. K. (2015). Recent insights into the cellular biology of atherosclerosis. J. Cell Biol. 209 (1), 13–22. 10.1083/jcb.201412052 25869663 PMC4395483

[B228] Tambralli A. Harbaugh A. NaveenKumar S. K. Radyk M. D. Rysenga C. E. Sabb K. (2024). Neutrophil glucose flux as a therapeutic target in antiphospholipid syndrome. J. Clin. Invest., e169893. 10.1172/JCI169893 38869951 PMC11290966

[B229] Tannahill G. M. Curtis A. M. Adamik J. Palsson-McDermott E. M. McGettrick A. F. Goel G. (2013). Succinate is an inflammatory signal that induces IL-1β through HIF-1α. Nature 496 (7444), 238–242. 10.1038/nature11986 23535595 PMC4031686

[B230] Tawakol A. Singh P. Mojena M. Pimentel-Santillana M. Emami H. MacNabb M. (2015). HIF-1α and PFKFB3 mediate a tight relationship between proinflammatory activation and anerobic metabolism in atherosclerotic macrophages. Arteriosclerosis, thrombosis, Vasc. Biol. 35 (6), 1463–1471. 10.1161/ATVBAHA.115.305551 PMC4441599 25882065

[B231] TeSlaa T. Ralser M. Fan J. Rabinowitz J. D. (2023). The pentose phosphate pathway in health and disease. Nat. Metab. 5 (8), 1275–1289. 10.1038/s42255-023-00863-2 37612403 PMC11251397

[B232] Theodorou K. Boon R. A. (2018). Endothelial cell metabolism in atherosclerosis. Front. Cell Dev. Biol. 6, 82. 10.3389/fcell.2018.00082 30131957 PMC6090045

[B233] Tillie R. De Bruijn J. Perales-Patón J. Temmerman L. Ghosheh Y. Van Kuijk K. (2021). Partial inhibition of the 6-phosphofructo-2-kinase/fructose-2,6-bisphosphatase-3 (PFKFB3) enzyme in myeloid cells does not affect atherosclerosis. Front. Cell Dev. Biol. 9, 695684. 10.3389/fcell.2021.695684 34458258 PMC8387953

[B234] Tomas L. Edsfeldt A. Mollet I. G. Perisic Matic L. Prehn C. Adamski J. (2018). Altered metabolism distinguishes high-risk from stable carotid atherosclerotic plaques. Eur. Heart J. 39 (24), 2301–2310. 10.1093/eurheartj/ehy124 29562241 PMC6012762

[B235] Tousoulis D. Kampoli A. M. Tentolouris C. Papageorgiou N. Stefanadis C. (2012). The role of nitric oxide on endothelial function. Curr. Vasc. Pharmacol. 10 (1), 4–18. 10.2174/157016112798829760 22112350

[B236] Townsend N. Kazakiewicz D. Lucy Wright F. Timmis A. Huculeci R. Torbica A. (2022). Epidemiology of cardiovascular disease in Europe. Nat. Rev. Cardiol. 19 (2), 133–143. 10.1038/s41569-021-00607-3 34497402

[B237] Tsai J. C. Perrella M. A. Yoshizumi M. Hsieh C. M. Haber E. Schlegel R. (1994). Promotion of vascular smooth muscle cell growth by homocysteine: a link to atherosclerosis. Proc. Natl. Acad. Sci. U. S. A. 91 (14), 6369–6373. 10.1073/pnas.91.14.6369 8022789 PMC44203

[B238] Tzeng H. P. Lan K. C. Yang T. H. Chung M. N. Liu S. H. (2017). Benzo[a]pyrene activates interleukin-6 induction and suppresses nitric oxide-induced apoptosis in rat vascular smooth muscle cells. PLoS One 12 (5), e0178063. 10.1371/journal.pone.0178063 28531207 PMC5439712

[B239] Umar S. Palasiewicz K. Volin M. V. Romay B. Rahat R. Tetali C. (2021). Metabolic regulation of RA macrophages is distinct from RA fibroblasts and blockade of glycolysis alleviates inflammatory phenotype in both cell types. Cell Mol. Life Sci. 78 (23), 7693–7707. 10.1007/s00018-021-03978-5 34705053 PMC8739866

[B240] Valdivielso J. M. Rodríguez-Puyol D. Pascual J. Barrios C. Bermúdez-López M. Sánchez-Niño M. D. (2019). Atherosclerosis in chronic kidney disease: more, less, or just different? Arteriosclerosis, thrombosis, Vasc. Biol. 39 (10), 1938–1966. 10.1161/ATVBAHA.119.312705 31412740

[B241] Van den Bossche J. O'Neill L. A. Menon D. (2017). Macrophage immunometabolism: where are we (going)? Trends Immunol. 38 (6), 395–406. 10.1016/j.it.2017.03.001 28396078

[B242] van der Valk F. M. Sluimer J. C. Vöö S. A. Verberne H. J. Nederveen A. J. Windhorst A. D. (2015). *In vivo* imaging of hypoxia in atherosclerotic plaques in humans. JACC Cardiovasc Imaging 8 (11), 1340–1341. 10.1016/j.jcmg.2014.12.015 25981502

[B243] van der Windt G. J. Pearce E. L. (2012). Metabolic switching and fuel choice during T-cell differentiation and memory development. Immunol. Rev. 249 (1), 27–42. 10.1111/j.1600-065X.2012.01150.x 22889213 PMC3645891

[B244] van Kuijk K. Demandt J. A. F. Perales-Patón J. Theelen T. L. Kuppe C. Marsch E. (2022). Deficiency of myeloid PHD proteins aggravates atherogenesis via macrophage apoptosis and paracrine fibrotic signalling. Cardiovasc Res. 118 (5), 1232–1246. 10.1093/cvr/cvab152 33913468 PMC8953448

[B245] van Leeuwen M. Gijbels M. J. Duijvestijn A. Smook M. van de Gaar M. J. Heeringa P. (2008). Accumulation of myeloperoxidase-positive neutrophils in atherosclerotic lesions in LDLR-/- mice. Arteriosclerosis, thrombosis, Vasc. Biol. 28 (1), 84–89. 10.1161/ATVBAHA.107.154807 17991873

[B246] Vassiliou E. Farias-Pereira R. (2023). Impact of lipid metabolism on macrophage polarization: implications for inflammation and tumor immunity. Int. J. Mol. Sci. 24 (15), 12032. 10.3390/ijms241512032 37569407 PMC10418847

[B247] Vats D. Mukundan L. Odegaard J. I. Zhang L. Smith K. L. Morel C. R. (2006). Oxidative metabolism and PGC-1beta attenuate macrophage-mediated inflammation. Cell Metab. 4 (1), 13–24. 10.1016/j.cmet.2006.05.011 16814729 PMC1904486

[B248] Vion A. C. Kheloufi M. Hammoutene A. Poisson J. Lasselin J. Devue C. (2017). Autophagy is required for endothelial cell alignment and atheroprotection under physiological blood flow. Proc. Natl. Acad. Sci. U. S. A. 114 (41), E8675–E8684. 10.1073/pnas.1702223114 28973855 PMC5642679

[B249] Vondenhoff S. Schunk S. J. Noels H. (2024). Increased cardiovascular risk in patients with chronic kidney disease. Herz 49 (2), 95–104. 10.1007/s00059-024-05235-4 38416185 PMC10917854

[B250] Voss K. Hong H. S. Bader J. E. Sugiura A. Lyssiotis C. A. Rathmell J. C. (2021). A guide to interrogating immunometabolism. Nat. Rev. Immunol. 21 (10), 637–652. 10.1038/s41577-021-00529-8 33859379 PMC8478710

[B251] Wall V. Z. Barnhart S. Kanter J. E. Kramer F. Shimizu-Albergine M. Adhikari N. (2018). Smooth muscle glucose metabolism promotes monocyte recruitment and atherosclerosis in a mouse model of metabolic syndrome. JCI Insight 3 (11), e96544. 10.1172/jci.insight.96544 29875324 PMC6124428

[B252] Wang H. Zheng X. Liu B. Xia Y. Xin Z. Deng B. (2021). Aspartate metabolism facilitates IL-1β production in inflammatory macrophages. Front. Immunol. 12, 753092. 10.3389/fimmu.2021.753092 34745126 PMC8567039

[B253] Wang J. Yang P. Yu T. Gao M. Liu D. Zhang J. (2022). Lactylation of PKM2 suppresses inflammatory metabolic adaptation in pro-inflammatory macrophages. Int. J. Biol. Sci. 18 (16), 6210–6225. 10.7150/ijbs.75434 36439872 PMC9682528

[B254] Wang L. Chen Y. Li X. Zhang Y. Gulbins E. Zhang Y. (2016b). Enhancement of endothelial permeability by free fatty acid through lysosomal cathepsin B-mediated Nlrp3 inflammasome activation. Oncotarget 7 (45), 73229–73241. 10.18632/oncotarget.12302 27689324 PMC5341975

[B255] Wang L. Luo J. Y. Li B. Tian X. Y. Chen L. J. Huang Y. (2016a). Integrin-YAP/TAZ-JNK cascade mediates atheroprotective effect of unidirectional shear flow. Nature 540 (7634), 579–582. 10.1038/nature20602 27926730

[B256] Wang Q. Ding Y. Song P. Zhu H. Okon I. Ding Y. N. (2017). Tryptophan-derived 3-hydroxyanthranilic acid contributes to angiotensin II-induced abdominal aortic aneurysm formation in mice *in vivo* . Circulation 136 (23), 2271–2283. 10.1161/CIRCULATIONAHA.117.030972 28978552 PMC5716872

[B257] Wang X. P. Zhang W. Liu X. Q. Wang W. K. Yan F. Dong W. Q. (2014). Arginase I enhances atherosclerotic plaque stabilization by inhibiting inflammation and promoting smooth muscle cell proliferation. Eur. Heart J. 35 (14), 911–919. 10.1093/eurheartj/eht329 23999450

[B258] Wang Z. Zhang X. Lu S. Zhang C. Ma Z. Su R. (2023). Pairing of single-cell RNA analysis and T cell antigen receptor profiling indicates breakdown of T cell tolerance checkpoints in atherosclerosis. Nat. Cardiovasc Res. 2 (3), 290–306. 10.1038/s44161-023-00218-w 37621765 PMC10448629

[B259] Warnatsch A. Ioannou M. Wang Q. Papayannopoulos V. (2015). Inflammation. Neutrophil extracellular traps license macrophages for cytokine production in atherosclerosis. Science 349 (6245), 316–320. 10.1126/science.aaa8064 26185250 PMC4854322

[B260] Wei X. Song H. Yin L. Rizzo M. G. Sidhu R. Covey D. F. (2016). Fatty acid synthesis configures the plasma membrane for inflammation in diabetes. Nature 539 (7628), 294–298. 10.1038/nature20117 27806377 PMC5671339

[B261] Weisdorf D. J. Craddock P. R. Jacob H. S. (1982). Granulocytes utilize different energy sources for movement and phagocytosis. Inflammation 6 (3), 245–256. 10.1007/BF00916406 6813260

[B262] Werle M. Kreuzer J. Höfele J. Elsässer A. Ackermann C. Katus H. A. (2005). Metabolic control analysis of the Warburg-effect in proliferating vascular smooth muscle cells. J. Biomed. Sci. 12 (5), 827–834. 10.1007/s11373-005-9010-5 16205843

[B263] Wilhelm K. Happel K. Eelen G. Schoors S. Oellerich M. F. Lim R. (2016). FOXO1 couples metabolic activity and growth state in the vascular endothelium. Nature 529 (7585), 216–220. 10.1038/nature16498 26735015 PMC5380221

[B264] Williams N. C. O’Neill L. A. J. (2018). A role for the krebs cycle intermediate citrate in metabolic reprogramming in innate immunity and inflammation. Front. Immunol. 9, 141. 10.3389/fimmu.2018.00141 29459863 PMC5807345

[B265] Wolf D. Gerhardt T. Winkels H. Michel N. A. Pramod A. B. Ghosheh Y. (2020). Pathogenic autoimmunity in atherosclerosis evolves from initially protective apolipoprotein B(100)-reactive CD4(+) T-regulatory cells. Circulation 142 (13), 1279–1293. 10.1161/CIRCULATIONAHA.119.042863 32703007 PMC7515473

[B266] Won J. C. Park J. Y. Kim Y. M. Koh E. H. Seol S. Jeon B. H. (2010). Peroxisome proliferator-activated receptor-gamma coactivator 1-alpha overexpression prevents endothelial apoptosis by increasing ATP/ADP translocase activity. Arteriosclerosis, thrombosis, Vasc. Biol. 30 (2), 290–297. 10.1161/ATVBAHA.109.198721 19965780

[B267] Wong S. L. Demers M. Martinod K. Gallant M. Wang Y. Goldfine A. B. (2015). Diabetes primes neutrophils to undergo NETosis, which impairs wound healing. Nat. Med. 21 (7), 815–819. 10.1038/nm.3887 26076037 PMC4631120

[B268] Wu D. Huang R. T. Hamanaka R. B. Krause M. Oh M. J. Kuo C. H. (2017). HIF-1α is required for disturbed flow-induced metabolic reprogramming in human and porcine vascular endothelium. Elife 6, e25217. 10.7554/eLife.25217 28556776 PMC5495571

[B269] Wu H. Wang M. Li X. Shao Y. (2021a). The metaflammatory and immunometabolic role of macrophages and microglia in diabetic retinopathy. Hum. Cell 34 (6), 1617–1628. 10.1007/s13577-021-00580-6 34324139

[B270] Wu Y. Ding Y. Ramprasath T. Zou M. H. (2021b). Oxidative stress, GTPCH1, and endothelial nitric oxide synthase uncoupling in hypertension. Antioxid. Redox Signal 34 (9), 750–764. 10.1089/ars.2020.8112 32363908 PMC7910417

[B271] Xiao L. Kirabo A. Wu J. Saleh M. A. Zhu L. Wang F. (2015). Renal denervation prevents immune cell activation and renal inflammation in angiotensin II-induced hypertension. Circ. Res. 117 (6), 547–557. 10.1161/CIRCRESAHA.115.306010 26156232 PMC4629828

[B272] Xiong J. Kawagishi H. Yan Y. Liu J. Wells Q. S. Edmunds L. R. (2018). A metabolic basis for endothelial-to-mesenchymal transition. Mol. Cell 69 (4), 689–698. 10.1016/j.molcel.2018.01.010 29429925 PMC5816688

[B273] Xu M. Cui Y. Wei S. Cong X. Chen Y. Tian S. (2024). Emerging nanomaterials targeting macrophage adapted to abnormal metabolism in cancer and atherosclerosis therapy (Review). Int. J. Mol. Med. 53 (2), 13. 10.3892/ijmm.2023.5337 38063240 PMC10760796

[B274] Xue L. Wang C. Qian Y. Zhu W. Liu L. Yang X. (2023b). Tryptophan metabolism regulates inflammatory macrophage polarization as a predictive factor for breast cancer immunotherapy. Int. Immunopharmacol. 125 (Pt B), 111196. 10.1016/j.intimp.2023.111196 37972471

[B275] Xue S. Su Z. Liu D. (2023a). Immunometabolism and immune response regulate macrophage function in atherosclerosis. Ageing Res. Rev. 90, 101993. 10.1016/j.arr.2023.101993 37379970

[B276] Yang K. Xu J. Fan M. Tu F. Wang X. Ha T. (2020). Lactate suppresses macrophage pro-inflammatory response to LPS stimulation by inhibition of YAP and NF-κB activation via GPR81-mediated signaling. Front. Immunol. 11, 587913. 10.3389/fimmu.2020.587913 33123172 PMC7573489

[B277] Yang L. Gao L. Nickel T. Yang J. Zhou J. Gilbertsen A. (2017). Lactate promotes synthetic phenotype in vascular smooth muscle cells. Circ. Res. 121 (11), 1251–1262. 10.1161/CIRCRESAHA.117.311819 29021296 PMC5681426

[B278] Yang Q. Xu J. Ma Q. Liu Z. Sudhahar V. Cao Y. (2018). PRKAA1/AMPKα1-driven glycolysis in endothelial cells exposed to disturbed flow protects against atherosclerosis. Nat. Commun. 9 (1), 4667. 10.1038/s41467-018-07132-x 30405100 PMC6220207

[B279] Yao J. Wu D. Qiu Y. (2022). Adipose tissue macrophage in obesity-associated metabolic diseases. Front. Immunol. 13, 977485. 10.3389/fimmu.2022.977485 36119080 PMC9478335

[B280] Yu P. Wilhelm K. Dubrac A. Tung J. K. Alves T. C. Fang J. S. (2017). FGF-dependent metabolic control of vascular development. Nature 545 (7653), 224–228. 10.1038/nature22322 28467822 PMC5427179

[B281] Yurdagul A. Jr. Subramanian M. Wang X. Crown S. B. Ilkayeva O. R. Darville L. (2020). Macrophage metabolism of apoptotic cell-derived arginine promotes continual efferocytosis and resolution of injury. Cell Metab. 31 (3), 518–533. 10.1016/j.cmet.2020.01.001 32004476 PMC7173557

[B282] Zaheer M. Chrysostomou P. Papademetriou V. (2016). “Hypertension and atherosclerosis: pathophysiology, mechanisms and benefits of BP control,” in Hypertension and cardiovascular disease. Editor Andreadis E. A. (Springer International Publishing), 201–216.

[B283] Zernecke A. Bot I. Djalali-Talab Y. Shagdarsuren E. Bidzhekov K. Meiler S. (2008). Protective role of CXC receptor 4/CXC ligand 12 unveils the importance of neutrophils in atherosclerosis. Circ. Res. 102 (2), 209–217. 10.1161/CIRCRESAHA.107.160697 17991882

[B284] Zhang D. Tang Z. Huang H. Zhou G. Cui C. Weng Y. (2019). Metabolic regulation of gene expression by histone lactylation. Nature 574 (7779), 575–580. 10.1038/s41586-019-1678-1 31645732 PMC6818755

[B285] Zhang Q. Chen S. Guo Y. He F. Fu J. Ren W. (2023). Phenylalanine diminishes M1 macrophage inflammation. Sci. China Life Sci. 66 (12), 2862–2876. 10.1007/s11427-022-2296-0 37243947

[B286] Zhao D. Liu J. Wang M. Zhang X. Zhou M. (2019). Epidemiology of cardiovascular disease in China: current features and implications. Nat. Rev. Cardiol. 16 (4), 203–212. 10.1038/s41569-018-0119-4 30467329

[B287] Zhao N. Yu X. Zhu X. Song Y. Gao F. Yu B. (2024). Diabetes mellitus to accelerated atherosclerosis: shared cellular and molecular mechanisms in glucose and lipid metabolism. J. Cardiovasc Transl. Res. 17 (1), 133–152. 10.1007/s12265-023-10470-x 38091232

[B288] Zhao X. Tan F. Cao X. Cao Z. Li B. Shen Z. (2020). PKM2-dependent glycolysis promotes the proliferation and migration of vascular smooth muscle cells during atherosclerosis. Acta Biochim. Biophys. Sin. (Shanghai) 52 (1), 9–17. 10.1093/abbs/gmz135 31867609

[B289] Zhao Y. Vanhoutte P. M. Leung S. W. (2015). Vascular nitric oxide: beyond eNOS. J. Pharmacol. Sci. 129 (2), 83–94. 10.1016/j.jphs.2015.09.002 26499181

[B290] Zhu Y. Ma W. Q. Han X. Q. Wang Y. Wang X. Liu N. F. (2018). Advanced glycation end products accelerate calcification in VSMCs through HIF-1α/PDK4 activation and suppress glucose metabolism. Sci. Rep. 8 (1), 13730. 10.1038/s41598-018-31877-6 30213959 PMC6137084

